# Switchable highly regioselective synthesis of 3,4-dihydroquinoxalin-2(1*H*)ones from *o*-phenylenediamines and aroylpyruvates

**DOI:** 10.3762/bjoc.13.132

**Published:** 2017-07-10

**Authors:** Juraj Dobiaš, Marek Ondruš, Gabriela Addová, Andrej Boháč

**Affiliations:** 1 Department of Organic Chemistry, Faculty of Natural Sciences, Comenius University in Bratislava, Mlynská dolina, Ilkovičova 6, 842 15 Bratislava, Slovakia; 2 Institute of Chemistry, Faculty of Natural Sciences, Comenius University in Bratislava, Mlynská dolina, Ilkovičova 6, 842 15 Bratislava, Slovakia; 3 Biomagi, Ltd., Mamateyova 26, 851 04 Bratislava, Slovakia

**Keywords:** controlled regioselectivity, cyclocondensation, 3,4-dihydroquinoxaline-2(1*H*)one, HOBt/DIC, mechanism

## Abstract

3-Acylmethylidene-3,4-dihydroquinoxalin-2(1*H*)-ones are compounds which possess a wide range of physical and pharmaceutical applications. These compounds can be easily prepared by cyclocondensation of *o*-phenylenediamines and aroylpyruvates. Unsymmetrically substituted *o*-phenylenediamines can be obtained form regioisomeric mixtures of 3,4-dihydroquinoxalin-2(1*H*)-ones. It is often quite difficult to get a pure regioisomer and determine its structure without controlling the reaction selectivity and exploitation of complex NMR techniques (HSQC, NOESY, HMBC). This article examines the regioselectivity of the cyclocondensation between six monosubstituted *o*-phenylenediamines (-OMe, -F, -Cl, -COOH, -CN, -NO_2_) and the derivatives of *p*-chlorobenzoylpyruvate (ester or acid) which we studied. Six regioisomeric 3,4-dihydroquinoxalin-2(1*H*)-one pairs were selectively prepared and characterised. Based on our experiences, a simplified methodology for determining the structure of the regioisomers was proposed. We developed two general and highly selective methodologies starting from the same *o*-phenylenediamines and activated 4-chlorobenzoylpyruvates (ester or acid) enabling switching of 3,4-dihydroquinoxalin-2(1*H*)-one regioselectivity in a predictable manner. For comparison, all regioselective cyclocondensations were performed with the same standardized conditions (DMF, rt, 3 days), differing only by the additives *p*-TsOH or HOBt/DIC (hydroxybenzotriazole/*N*,*N’*-diisopropylcarbodiimide). Both selected methods are simple, general and highly regioselective (72–97%). A mechanism for the regioselectivity was also proposed and discussed. This study can be used as a guide when choosing the most optimal reaction conditions for the synthesis of the desired 3,4-dihydroquinoxalin-2(1*H*)-one regioisomers with the best selectivity. The demonstrated methodologies in this article may also be applied to differently substituted 3,4-dihydroquinoxalin-2(1*H*)-ones in general, which could expand the synthetic impact of our results.

## Introduction

**Scheme 1:**
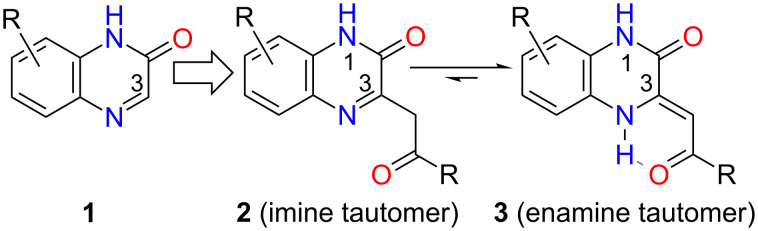
The structures of quinoxalin-2(1*H*)-ones **1**, **2** and 3,4-dihydroquinoxalin-2(1*H*)-ones **3**. An acylmethyl group in imines **2** shifts their tautomeric equilibrium to enamines **3**.

The quinoxalin-2(1*H*)-one moiety is frequently found in compounds that exhibit biological activity, particularly antimicrobial, anticancer, anti-HIV, antithrombotic, analgesic and antidiabetic [1–3]. Substitution on C(3) of quinoxalin-2(1*H*)-ones **1** by substituents that possess a carbonyl group in a β side chain position **2**, **3** significantly alters their chemical properties as suggested by ^1^H NMR, IR spectra and X-ray crystallography of the studied derivatives [4–5]. In this case, their tautomeric equilibrium is shifted to enamines **3** that have been stabilized by an intramolecular hydrogen bond (Scheme 1). A new pseudo-ring is formed via the hydrogen bond in **3**, which further spreads the planarity of the 3,4-dihydroquinoxalin-2(1*H*)-one system.

**Figure 1:**
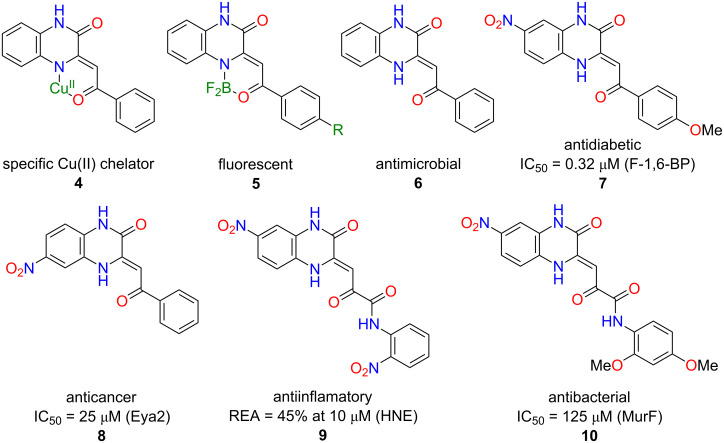
The structures including some of their physical and biological properties of 3,4-dihydroqunoxalin-2(1*H*)-ones **4–10**. Abbreviations used: F-1,6-BP = fructose 1,6-bisphosphatase; Eya2 = eyes absent homolog 2; HNE = human neutrophil elastase; MurF = muramyl ligase F; REA = remaining activity.

The keto–enamine arrangement in **3** is capable of specific binding of Cu(II) ions like in **4**, which was proved by red shifts in the UV–vis spectra [6–8]. The pseudo ring can also accommodate BF_2_ moiety, yielding compounds like **5** with interesting fluorescent properties [9–10]. Compounds **3** have described similar biological activities to the parent quinoxalin-2(1*H*)-ones **1**. Mashevskaya et al. reported about an antimicrobial activity for compound **6** at 1 mg/mL for *S. aureus* P-209 and *E. coli* M_17_ strains [11]. Several recent studies have identified 3,4-dihydroquinoxalin-2(1*H*)-ones **7–10** as hits for distinct biological targets [12–20], particularly antidiabetic **7** [20], anticancer **8** [18], anti-inflammatory **9** [16], and antibacterial **10** [19] (Figure 1).

The described compounds **4–6** were prepared from *o*-phenylenediamine. Compounds **7–10** may be prepared from monosubstituted *o*-phenylenediamines **11**. In this case the amino groups usually have different reactivity and thus produce a mixture of regioisomeric products. If both regioisomers are needed, one can separate them [21]. Separation can be difficult to such an extent that some of the Cu(II) chelators were characterised as regioisomeric mixtures [22]. Nevertheless, most of the nonsymmetrical 3,4-dihydroquinoxalin-2(1*H*)-ones are required in their pure form (Figure 1).

**Scheme 2:**
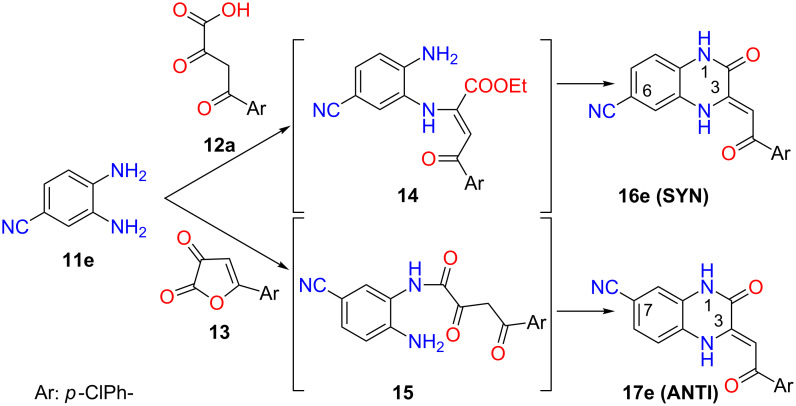
Selective synthesis of both 3,4-dihydroquinoxalin-2(1*H*)-one regioisomers **16e (SYN)** and **17e (ANTI)**.

To the best of our knowledge, there is only one paper which deals with the regioselective synthesis of 3-acylmethylidene-3,4-dihydroquinoxalin-2(1*H*)-ones **3**. Andreichikov et al. reported controlled synthesis of both SYN and ANTI regioisomers [23] (Scheme 2). For the sake of simplicity, we introduced SYN and ANTI descriptors for regioisomers according to the relative positions of two substituents on 3,4-dihydroquinoxalin-2(1*H*)-one skeleton (e.g., -CN and 4-chlorobenzoylmethylidene groups in **16e (SYN)** and **17e (ANTI)**). The reaction of 3,4-diaminobenzonitrile (**11e**) with α,γ-diketoester **12a** provided the product **16e (SYN)** through an enamine intermediate **14**, formed by the reaction of the most reactive α-keto group in **12a** with the more nucleophilic *m*-amino group of **11e**. The opposite regioisomer **17e (ANTI)** was selectively prepared by reaction of the more nucleophilic *m*-amino group of **11e** with the most reactive lactonic carbonyl group in 5-(4-chlorophenyl)furan-2,3-dione (**13**) through an amide intermediate **15** (Scheme 2).

**Scheme 3:**
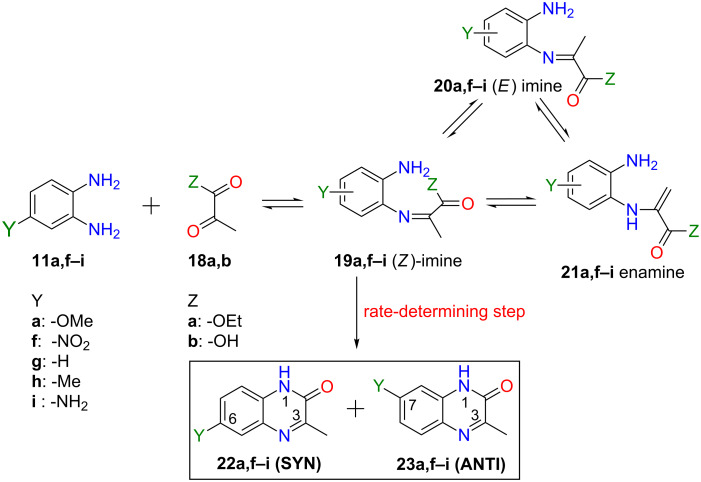
The proposed mechanism for the synthesis of 3-methylquinoxalin-2(1*H*)-one regioisomers **22** and **23**. In the case of starting **11g** (Y: -H), **22g** is the same as **23g** and no SYN/ANTI regioisomerism exists.

Abasolo et al. studied the reaction of monosubstituted *o*-phenylenediamines **11a,f–i** with pyruvic ethyl ester (**18a**) or its acid **18b** and postulated that an equilibrium between *Z E* imines **19**, **20** and enamine **21** is quickly reached and the rate-determining reaction step is a subsequent intramolecular cyclization yielding the regioisomeric products **22** and **23** [24] (Scheme 3). The proposed mechanism was based on the isolation of the 4-nitrodiaminobenzene imine intermediate which possessed reduced reactivity for intramolecular cyclisation. This type of reaction was exploited in the literature several times for the synthesis of quinoxalin-2(1*H*)-one regioisomers [10,25–26].

**Scheme 4:**
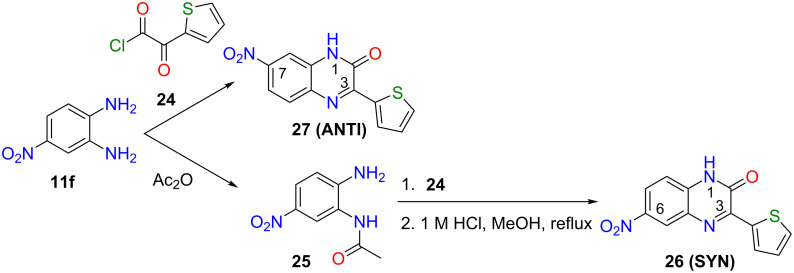
The regioselective syntheses of both quinoxalin-2(1*H*)-ones **27 (ANTI)** and **26 (SYN)**.

Cyclocondensation between **11f** and **24** provided mainly quinoxalin-2(1*H*)-one **27 (ANTI)**. Sherman et al. prepared the opposite regioisomer **26 (SYN)** by masking the more reactive amino group in **11f** through the acetamide intermediate **25** and its subsequent reaction with acid chloride **24**, forming a stable *N*-acetamide-*N’*-acylamide intermediate (not shown). The acetamide group was then selectively cleaved and the liberated amino group spontaneously cyclised to SYN quinoxalin-2(1*H*)-one **26** [27] (Scheme 4).

Complete regioselectivity can be obtained if one does not begin from the substituted *o*-phenylenediamine **11**. Sakata et al. reported an interesting one-pot procedure yielding 6-substituted SYN quinoxalin-2(1*H*)-ones from substituted *N*-(2-nitrophenyl)-3-oxobutanamides [28].

**Scheme 5:**
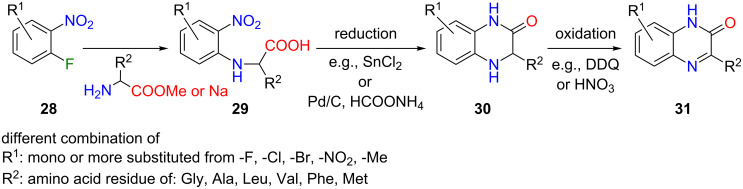
The selective synthesis of substituted quinoxalin-2(1*H*)-ones **31** from **28** via three reaction steps.

Another example for preparation of the desired regioisomer starts from the nucleophilic substitution of o-fluoronitrobenzenes **28** with the derivatives of α-amino acids [29–31]. The product of spontaneous cyclization of **30** was obtained after reduction of nitro derivative **29**. Through mild oxidation, compound **30** provides the required quinoxalin-2(1*H*)-one **31** (Scheme 5). The obvious disadvantage in this case is the initial aromatic substitution step from **28** to **29** and the additional two reactions to **31**, compared to the direct synthesis of both regioisomers depicted in Scheme 2.

## Results and Discussion

A study to control the regioselectivity in the synthesis of **17d (ANTI)** (Table 1) was motivated by our ongoing medicinal chemistry research. According to the literature, the synthesis of 3-acylmethylidene-3,4-dihydroquinoxalin-2(1*H*)-ones **3** can be performed by simple cyclocondensation of an appropriate *o*-phenylenediamine **11** with α,γ-diketoester **12a** (Scheme 2). In our case, starting from 3,4-diaminobenzoic acid (**11d**) (natively preferring SYN regioselectivity, see Table 1, entry 4, column 3) complicated the desired reaction. The other possibility was to perform the regioselective synthesis of **17d (ANTI)** from an appropriate furan-2,3-dione **13** (Scheme 2). The preparation of **13** failed in our hands. Therefore, we decided to perform the cyclocondensation of **11d** with ethyl 4-chlorobenzoylpyruvate (**12a**) and separate the less abundant **17d (ANTI)** from a mixture of regioisomers. The cyclocondensation of **11d** with **12a** in refluxing dioxane within 1 hour yielded a precipitate that consisted of a balanced (50/50) mixture of both SYN/ANTI regioisomers **16d** and **17d** (confirmed by ^1^H NMR). The low solubility of the crude products in DCM, THF, EtOAc or MeOH combined with an unfavourable ratio of regioisomers prevented their separation by FLC or crystallisation. Based on these results we realised that it would be difficult to obtain pure ANTI regioisomer **17d** without controlling the reaction selectivity. In order to influence the regioselectivity, cyclocondensation of **11d** with **12a** was performed in the presence of basic TEA (Et_3_N) in DMF at rt within 3 days. The reaction yielded a crude mixture with a predominant **17d (ANTI)** regioisomer (70/30, not shown elsewhere). For the same reaction, we also used less basic DMAP instead of TEA. This cyclocondensation formed a crude mixture with even better **17d (ANTI)** selectivity (88/12) (Table 1, entry 4, column 5). DMAP is also known as an acyl activation reagent (similar to: HOBt, HATU, T3P, etc.). Therefore, we concluded that switching in regioselectivity (from native SYN to ANTI) could be a matter of DMAP activation of the acyl group in **12a**.

**Table 1:** The regioisomeric ratios (**17 (ANTI)/16 (SYN)**) and conversions depending on used diamine **11a–f**, dicarbonyl compound **12a**,**b** and reaction conditions.

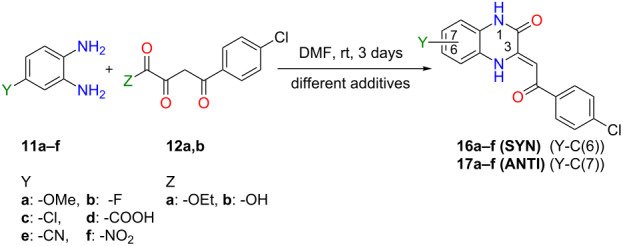						

Entry	Diamine **11a–f**	The below specified conditions dependent ratios^a^ and conversions.^b^				

		Ester **12a**^c^	Ester **12a**^c,d^	Acid **12b**^c,d^	Ester **12a**^c,e^	Acid **12b**^f^

**1**	**11a** (**-**OMe)	**85/15**^a^ (100%)^b^	**91/09** (100%)	67/33 (100%)	**14/86** (100%)	**15/85** (100%)
**2**	**11b** (**-**F)	80/20 (95%)	**89/11** (100%)	**85/15** (100%)	17/83 (80%)	**14/86** (100%)
**3**	**11c** (**-**Cl)	65/35 (100%)	72/28 (100%)	70/30 (100%)	28/72 (75%)	23/77 (100%)
**4**	**11d** (**-**COOH)	38/62 (100%)	**13/87** (100%)	**12/88** (100%)	**88/12** (50%)	**93/07** (100%)
**5**	**11e** (-CN)	17/83 (100%)	**15/85** (100%)	**11/89** (100%)	**100/0** (25%)	**93/07** (100%)
**6**	**11f** (**-**NO_2_)	18/82 (90%)	20/80 (100%)	**05/95** (100%)	68/32 (22%)	**97/03** (100%)

^a^The ratios for both regioisomers **16** and **17** (mol %) were determined by ^1^H NMR spectra from evaporated reaction mixtures (based on characteristic singlet signals of each regioisomer =CHCO– (6.8–6.9 ppm)) and are depicted here in the order **17 (ANTI)/16 (SYN)**. The ratios marked in bold represent the best selectivities with cut-off ≥85% for the main ANTI or SYN regioisomer. ^b^The % number in brackets describes conversion of the appropriate starting *o*-phenylenediamine **11a–f** obtained from the crude ^1^H NMR spectra. ^c^General procedure A was used. ^d^TsOH (1 mol equiv) was added. ^e^DMAP (1 mol equiv) was added. Reaction proceeds through in situ formed intermediate **12c** (Scheme 6). ^f^General procedure B (HOBt/DIC) was used. Reaction proceeds through in situ formed intermediate **12d** (Scheme 6).

**Scheme 6:**
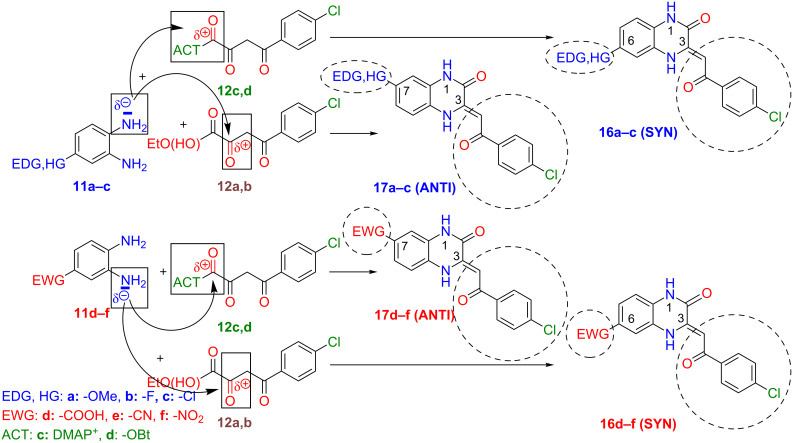
Regioselectivity switching based on carbonyl activation of 4-chlorobenzoylpyruvates **12a**,**b** by *p*-TsOH or acyl activation in **12c**,**d** (prepared in situ from ester **12a** by DMAP – **12c** or from acid **12b** and HOBt/DIC – **12d**).

**Figure 2:**
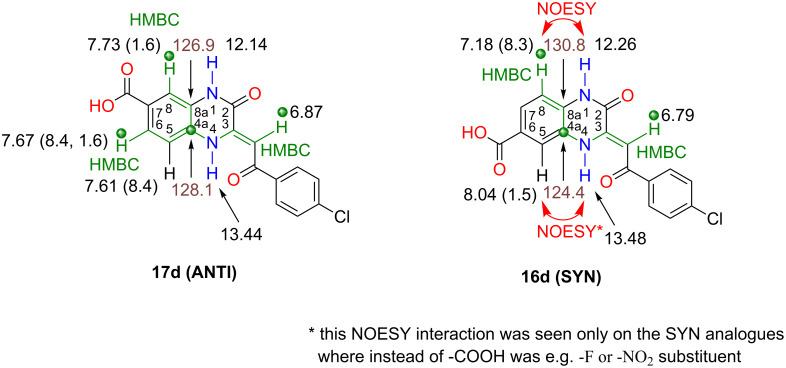
The interactions and assignments, obtained after analyses of NMR spectra, allowed us to distinguish between the **16d** and **17d** regioisomers. The important ^1^H and ^13^C NMR chemical shifts are depicted close to each appropriate atom in the structures accompanied with an coupling constant in brackets, if there was any. The double-headed red arrows represent important space interactions observed in NOESY spectra. The green-coloured bonds in the structures represent some of the most important HMBC interactions that helped us when assigning the right regioisomers. The HMBC signals between the skeletal proton and carbon are limited to 2, 3 and sometimes also to 4 bonds.

The above controlled ANTI cyclocondensation (88/12) performed in the presence of DMAP enabled us to obtain pure **17d (ANTI)** regioisomer in a 36% yield after crystallisation from DMSO. To prove the exact structure of **17d (ANTI)** was not easy and we had to combine the more complex 2D NMR techniques (HSQC, NOESY and HMBC). At first, we assigned hydrogens and carbons in the main regioisomer. Next, we proved for **17d** its ANTI isomerism by HMBC analysis, which showed an important four-bonded interaction between the hydrogen of the methylene C(3)=CH- group and the quaternary carbon C(4a) (Figure 2). The carbon C(4a) was interconnected in the HMBC with 2 other three-bonded interactions with hydrogen H-C(8) and H-C(6), whereas for the **16d (SYN)** isomer the H-C(6) interaction should be missing (see also the HMBC diagrams and spectra in Supporting Information File 1).

After we had proved the structure of the **17d (ANTI)** regioisomer, we decided to elucidate the structure for the **16d (SYN)** isomer as well, in order to complementary validate the above assignments. In regards to the absence of regioselectivity by heating of 3,4-diaminobenzoic acid (**11d**, -COOH) with **12a** in dioxane for 1 hour (50/50), a reaction under milder conditions (DMF, rt, 3 days) was performed resulting in ANTI/SYN selectivity (38/62, Table 1, entry 4, columns 3 and 4). In order to positively influence the regioselectivity, cyclocondensation was performed in the presence of *p*-TsOH (DMF, rt, 3 days). This reaction resulted in high SYN regioselectivity (13/87). The enhanced selectivity was explained by the elevation of electrophilicity of the α-ketone in **12a** by *p*-TsOH. After the crystallisation from DMSO, pure **16d (SYN)** was isolated. We elucidated its structure in similar manner as for the **17d (ANTI)** isomer (Figure 2) and confirmed that the ^1^H NMR (DMSO*-d*_6_) higher shifted signal for the H-N(4) group is the one that forms a intramolecular hydrogen bond with the carbonyl group from a side chain like in **3** (Scheme 1). This knowledge, in combination with a simple NOE experiment (interaction H-N(4) with a narrow doublet of H-C(5) (ca 1.5 Hz) in SYN or H-N(4) with a broad doublet of H-C(5) (8.4 Hz) in ANTI, allows for the determination of the regioisomerism more effectively (no need for HMBC), in the case that the NOE experiment was performing (which was not always the case). The same approach to distinguish regioisomers can be applied on space interactions between H-N(1) and H-C(8) signals (NOESY in Figure 2 or for NOE see the Supporting Information File 1).

Finally, all six regioisomeric 3,4-dihydroquinoxalin-2(1*H*)-one pairs were selectively prepared and characterised (Table 1 and Supporting Information File 1). Their condensations were investigated under five different reaction conditions:

a) Cyclocondensations of diamines **11a–f** and ethyl ester of **12a** were performed by our standardized conditions: DMF, rt, 3 days (General procedure A). In all cases, the most nucleophilic amine from diamine **11** predominantly reacts with the most reactive α-carbonyl group from ester **12a** to form an imine/enamine bond followed by amidic intramolecular cyclization to yield the main regioisomer of 3,4-dihydroquinoxalin-2(1*H*)-one. The regioselectivity was dependent on the character of substituent Y in diamines **11a–f** (Table 1). According to obtained selectivities (Table 1), the substituents on diamines **11** were divided into two clusters causing either the same or the contrary regioselectivity: (a) with electron-donating groups and halogens (EDG or HG) or (b) with electron-withdrawing (EWG) groups (Table 1, Scheme 6). Using the general procedure A without any additives, diamine **11** was always almost quantitatively consumed (90–100%), and for both clusters of substituents on **11**, the main regioisomer of 3,4-dihydroquinoxalin-2(1*H*)-one was formed with average to good selectivity (62–85%) (Table 1, column 3).

b) Utilizing the general procedure A with *p*-TsOH as an additive to diamine from **11a–f** and ester **12a** caused greater reactivity of the α-carbonyl group in **12a** (Scheme 6), quantitative consumption of diamine **11**, and production of the main regioisomer of 3,4-dihydroquinoxalin-2(1*H*)-one with good to excellent selectivity (72–91%) for both clusters of substituents (Table 1, column 4).

c) Ester **12a** to acid **12b** modification with other parameters as in (b) resulted in the preservation of complete conversions of **11**, a decrease of ANTI selectivity (67–85%) for EDG/HG substituents in **11a–c** (Table 1, entries 1–3, column 5), however, a SYN selectivity boost (88–95%) for EWG substituents in **11d–f** (Table 1, entries 4–6, column 5).

d) In order to investigate the contrary (switched) regioselectivity, DMAP was added to diamine from **11a–f** and ester **12a**, and standard general procedure A was used. In these cases, DMAP activated the acyl moiety in ester **12a** via **12c** (Scheme 6) and caused the formation of the predominant regioisomer of 3,4-dihydroquinoxalin-2(1*H*)-one with reversed selectivity as determined before (a)–(c). The consumption of **11a–c** (Table 1, entries 1–3, column 6) was good (75–100%), but insufficient for **11d–f** (22–50%, Table 1, entries 4–6, column 6). Our explanation for the low conversions is a diminished amine nucleophilicity in the EWG-substituted dianilines **11d–f** and reduced electrophilicity of the **12a** due to a partial formation of a stabilized enolate anion by DMAP deprotonation. Therefore, using DMAP activation is not recommended for EWG-substituted diamines.

e) The reaction of activated ester **12d** (obtained in situ after treatment of carboxylic acid **12b** with HOBt/DIC, Scheme 6) and a diamine **11a–f** (general procedure B) was found to be the most convenient method to produce contrary product regioselelectivity. In this case, the consumption of diamine **11** was always quantitative. The regioselectivity was opposite in comparison to the reactions from (a)–(c) and similar to the reaction exploiting DMAP activation of ester **12a** in (d). HOBt/DIC activation of acid **12b** resulted in the synthesis of the main regioisomers of 3,4-dihydroquinoxalin-2(1*H*)-ones with good to excellent SYN selectivity (77–86%) for EDG/HG cluster of substituents in **11a–c** (Table 1, entries 1–3, column 7) and excellent ANTI selectivity (93–97%) for EWG substituents in **11d–f** (Table 1, entries 4–6, column 7). The HOBt/DIC methodology seems to be better than the one described with DMAP due to its clean reaction course and high regioselectivity in general (Table 1, column 7). The HOBt methodology is complementary in regioselectivity to cycloadditions performed by *p*-TsOH (Table 1, column 4).

From the above experimental results, we have proved that the regioselectivity of the cyclocondensation depends on both (a) the different nucleophilicity of the two amines from *o-*phenylenediamines **11a–f** and (b) the electrophilicity of the α-carbonyl in **12a** or the contrary regioselectivity of activated species **12c**,**d** (prepared in situ from appropriate **12** by DMAP – **12c** or HOBt/DIC additives – **12d**) (Scheme 6).

## Conclusion

Simple reaction conditions were discovered for predictable and switchable highly regioselective synthesis of 3,4-dihydroquinoxaline-2(1*H*)-ones **16** or **17** starting from monosubstituted *o*-phenylenediamines **11** and 4-chlorobenzoylpyruvates **12** in DMF at rt. These conditions were tested by cyclocondensations on six diamines **11a–f** with two pyruvates **12a**,**b** and allowed us to prepare, purify and characterise twelve (six pairs) of regioisomeric 3,4-dihydroquinoxaline-2(1*H*)-ones. Their ANTI (**17**) or SYN (**16**) structures were assigned by complex 2D NMR techniques (HSQC, NOESY and HMBC) or by a proposed simplified method (NOE interaction between higher ^1^H NMR (DMSO-*d*_6_) shifted H-N(4), bonded by intramolecular hydrogen bond, and H-C(5) or interaction of lower shifted H-N(1) and H-C(8), whereby coupling of HC(5 and 8) depends on type of the regioisomer). It was proved that observed regioselectivity of performed cyclocondensations depends on both a/ the different nucleophilicity of amine groups in diamine **11** (with two clusters of substituents: EDGs + halogens (HG) and complementary operating EWGs) and (b) the different activation of 4-benzoylpyruvates **12a**,**b** (*p-*TsOH; contrarily by DMAP or HOBt/DIC). Obtained selectivities were discussed and their mechanism proposed (Scheme 6). Our study can act as a guide for choosing the optimal reaction conditions for the synthesis of the desired regioisomer of 3,4-dihydroquinoxaline-2(1*H*)-one **16** or **17** with the best selectivity (activation of **12a**,**b** by acid or opposite selectivity obtained from activated species **12c**,**d**) (Scheme 6, Table 2).

**Table 2:** The guide for reaction conditions for obtaining the desired regioisomer of 3,4-dihydroquinoxaline-2(1*H*)one **16 (SYN)** or **17 (ANTI)** with the best selectivity.

Starting pyruvate **12** with additive/ substituted diamine **11**	Ester **12a** Acid **12b** *p*-TsOH	Acid **12b** HOBt/DIC

**11a–c** (EDG or halogen)	**17 (ANTI)** (72–91%)	**16 (SYN)** (77–86%)
**11d–f** (EWG)	**16 (SYN)** (88–95%)	**17 (ANTI)** (93–97%)

A limitation for regioselectivity is the fact that it is dependent on the character of the substituent in aryldiamine **11**. If the nucleophilicity of the two amino groups in **11** are not differentiated enough, the obtained selectivity will be less synthetically useful. In that case, a different synthesis will be required, e.g., masking amino with the nitro group. The HOBt/DIC procedure performs with contrary regioselectivity to *p-*TsOH. We believe that the other acyl activated agents like T3P (propylphosphonic anhydride) or HATU should produce similar results as well. The demonstrated findings could be applied also to differently substituted 3,4-dihydroquinoxalin-2(1*H*)-ones in general, which can expand the synthetic impact of our study.

## Experimental

Syntheses and characterisation of compounds **16d (SYN)** and **17d (ANTI)** are described below. The synthesis of all prepared compounds **12a**,**b**, **16a–f (SYN)** and **17a–f (ANTI)** together with their characterisation, spectral diagrams and spectra can be found in Supporting Information File 1.

### General procedure A

A solution of ethyl 4-chlorobenzoylpyruvate (100 mg, 0.39 mmol, 1.00 equiv) (**12a**) or 4-chlorobenzoylpyruvic acid (**12b**) (88.4 mg, 0.39 mmol, 1.00 equiv), *o*-phenylenediamine **11a–f** (1.00 equiv) with or without an additive (1.00 equiv) (*p*-TsOH or DMAP) was stirred in 3.0 mL of DMF (abs) at rt under Ar for 72 hours. A low soluble mixture of ANTI/SYN regioisomers slowly precipitated within the reaction. The precipitate was collected by filtration or centrifugation, triturated by 3 mL of Et_2_O and crystallized from DMSO (if not otherwise stated) to yield the main solid regioisomer **16** or **17**.

### General procedure B

Diisopropylcarbodiimide 82 μL (66.9 mg, 0.53 mmol, 1.20 equiv, DIC) was added to a solution of 4-chlorobenzoylpyruvic acid (100 mg, 0.44 mmol, 1.00 equiv, **12b**) and 73.8 mg (0.53 mmol, 1.20 mol equiv) of HOBt [CAS: 123333-53-9, 97% wetted with ≥14 wt % H_2_O] in 3.0 mL of DMF (abs) under Ar. The reaction mixture was stirred for 5 min. Then *o-*phenylenediamine (1.00 equiv, **11a–f**) was added and the mixture was stirred at rt under Ar for 72 h. The precipitated product mixture obtained after filtration (or centrifugation) was triturated by 3 mL of Et_2_O and crystallized from DMSO (if not otherwise stated) to yield the main solid regioisomer **16** or **17**.

### Compound **16d (SYN)**

**Figure 3:**
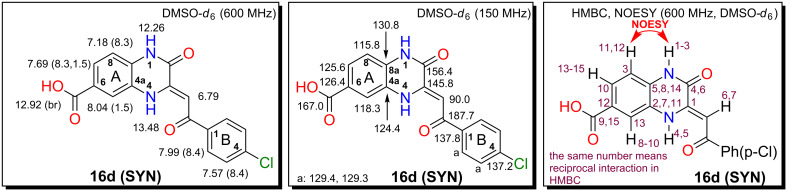
NMR assignments for compound **16d**.

The compound **16d (SYN)** (Figure 3) was prepared according the general procedure A from diamine **11d**, ester **12a** and *p-*TsOH as an additive. The crude mixture of ANTI/SYN regioisomers was purified by crystallization from DMSO to yield 64.6 mg (0.19 mmol, 48%) of **16d (SYN)**. Mp: 363.0–365.0 °C (DMSO), yellow solid compound. ^1^H NMR (600 MHz, DMSO-*d*_6_) δ 13.48 (s, 1H, H**-**N_A_(4)), 12.92 (br s, 1H, -COOH), 12.26 (s, 1H, H**-**N_A_(1)), 8.04 (d, *J*(A_5_,A_7_) = 1.5 Hz, 1H, H**-**C_A_(5)), 7.99 (d, *J*(B_2_,B_3_) = 8.4 Hz, 2H, 2 × H**-**C_B_(2)), 7.69 (dd, *J*(A_7_,A_8_) = 8.3 Hz, *J*(A_5_,A_7_) = 1.5 Hz, 1H, H**-**C_A_(7)), 7.57 (d, *J*(B_2_,B_3_) = 8.4 Hz, 2H, 2 × H**-**C_B_(3)), 7.18 (d, *J*(A_7_,A_8_) = 8.3 Hz, 1H, H**-**C_A_(8)), 6.79 (s, 1H, -COCH=); ^13^C NMR (150 MHz, DMSO-*d*_6_) δ 187.7 (C_B_(1)C=O), 167.0 (-COOH), 156.4 (C_A_(2)), 145.8 (C_A_(3)), 137.8 (C_B_(1)), 137.2 (C_B_(4)), 130.8 (C_A_(8a)), 129.4 and 129.3 (2 × C_B_(2 and 3)), 126.4 (C_A_(6)), 125.6 (C_A_(7)), 124.4 (C_A_(4a)), 118.3 (C_A_(5)), 115.8 (C_A_(8)), 90.0 (-COCH=). FTIR cm^−1^ (solid): 3184 (s, -COOH), 2925 (m), 1732 (w), 1688 (s, C=O), 1615 (s), 1586 (s), 1550 (w), 1486 (w), 1366 (m), 1247 (m), 1218 (m), 1095 (m), 1065 (w), 1011 (w), 787 (w), 750 (m) cm^−1^; MS (ESI *m*/*z*): 341.2 [M − H]^−^; anal. calcd for C_17_H_11_ClN_2_O_4_ (342.73): C, 59.57; H, 3.23; Cl, 10.34; N, 8.17; found: C, 59.75; H, 3.38; Cl, 10.23; N, 8.01.

### Compound **17d (ANTI)**

**Figure 4:**
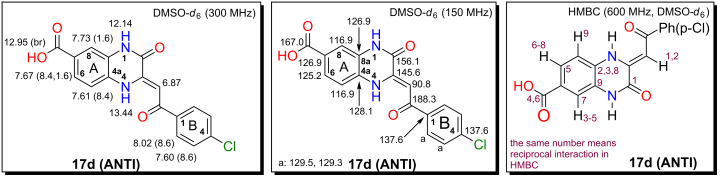
NMR assignments for compound **17d**.

Compound **17d (ANTI)** (Figure 4) was prepared according general procedure A from diamine **11d**, ester **12a** and (1.00 equiv) of DMAP as an additive. The crude product was crystallized from DMSO and obtained as salt with DMAP. To liberate the free acid **17d (ANTI)**, the salt was suspended in 1 M HCl, stirred for 24 hours, the solid material was filtered off, washed with water and dried to yield 48.4 mg (0.14 mmol, 36%) of **17d (ANTI)**.

Alternatively **17d (ANTI)** was also prepared according to general procedure B from diamine **11d**, acid **12b** and HOBt/DIC. The crude product was crystallized from DMSO to yield 105.9 mg (0.31 mmol, 70%) of **17d (ANTI)**. Mp: 391.0–392.0 °C [DMSO], yellow solid compound. ^1^H NMR (300 MHz, DMSO-*d*_6_) δ 13.44 (s, 1H, H**-**N_A_(4)), 12.95 (br s, 1H, -COOH), 12.14 (s, 1H, H**-**N_A_(1)), 8.02 (d, *J*(B_2_,B_3_) = 8.6 Hz, 2H, 2 × H**-**C_B_(2)), 7.73 (d, *J*(A_6_,A_8_) = 1.6 Hz, 1H, H**-**C_A_(8)), 7.67 (dd, *J*(A_5_,A_6_) = 8.4 Hz, *J*(A_6_,A_8_) = 1.6 Hz, 1H, H**-**C_A_(6)), 7.61 (d, *J*(A_5_,A_6_) = 8.4 Hz, 1H, H**-**C_A_(5)), 7.60 (d, *J*(B_2_,B_3_) = 8.6 Hz, 2H, 2 × H**-**C_B_(3)), 6.87 (s, 1H, -COCH=); ^13^C NMR (150 MHz, DMSO-*d*_6_) δ 188.3 (C_B_(1)C=O), 167.0 (-COOH), 156.1 (C_A_(2)=O), 145.6 (C_A_(3)), 2 × 137.6 (C_B_(1 and 4)), 129.5 and 129.3 (2 × C_B_(2 and 3)), 128.1 (C_A_(4a)), 2 × 126.9 (C_A_(7 and 8a)), 125.2 (C_A_(6)), 2 × 116.9 (C_A_(5 and 8)), 90.8 (-COCH=); FTIR cm^−1^ (solid): 3486 (m), 3206 (s, -COOH), 2634 (w), 1706 (s, C=O), 1661 (w), 1628 (m), 1586 (s, C=O), 1522 (w), 1398 (m), 1374 (w), 1291 (m), 1248 (m), 1184 (m), 1093 (w), 1056 (m), 1009 (w), 899 (w), 781 (w), 764 (w), 721 (w) cm^−1^; MS (ESI *m*/*z*): 341.0 [M − H]^−^; anal. calcd for C_17_H_11_ClN_2_O_4_ (342.73): C, 59.57; H, 3.23; N, 8.17; found: C, 59.82; H, 3.10; N, 8.32.

## Supporting Information

File 1 Additional experimental and characterisation data.
