# Supplementary material for: Switchable highly regioselective synthesis of 3,4-dihydroquinoxalin-2(1H)ones from o-phenylenediamines and aroylpyruvates
Source: Beilstein J Org Chem. 2017 Jul 10;13:1350–60. doi: 10.3762/bjoc.13.132 (PMC5530724; doi:10.3762/bjoc.13.132)
Supplement: File 1 — Additional experimental and characterisation data. [file Beilstein_J_Org_Chem-13-1350-s001.pdf]

Supporting Information

for

Switchable highly regioselective synthesis of 3,4-dihydroquinoxalin-2(1*H*)ones from *o*-phenylenediamines and aroylpyruvates

Juraj Dobiaš,[1*] Marek Ondruš,[1] Gabriela Addová,[2] and Andrej Boháč[1,3*]

Address: [1]Department of Organic Chemistry, Faculty of Natural Sciences, Comenius University in Bratislava, Mlynská dolina, Ilkovičova 6, 842 15 Bratislava, Slovakia, [2] Institute of Chemistry, Faculty of Natural Sciences, Comenius University in Bratislava, Mlynská dolina, Ilkovičova 6, 842 15 Bratislava, Slovakia and [3] Biomagi, Ltd., Mamateyova 26, 851 04 Bratislava, Slovakia

Email: Juraj Dobiaš* - jur.dobias@gmail.com

*Corresponding author

Additional experimental and characterisation data

Table of Contents

General information S2
Synthesis of compounds S3
Graphical abstract for Supporting Information S3
General procedures S3
General procedure A: S3
General procedure B: S3
Ethyl 4-chlorobenzoylpyruvate (**12a**) S4
4-Chlorobenzoylpyruvic acid (**12b**) S6
(*Z*)-3-(2-(4-Chlorophenyl)-2-oxoethylidene)-6-methoxy-3,4-dihydroquinoxalin-2(1*H*)-one (**16a (SYN)**) S8
(*Z*)-3-(2-(4-Chlorophenyl)-2-oxoethylidene)-7-methoxy-3,4-dihydroquinoxalin-2(1*H*)-one (**17a (ANTI)**) S11
(*Z*)-3-(2-(4-Chlorophenyl)-2-oxoethylidene)-6-fluoro-3,4-dihydroquinoxalin-2(1*H*)-one (**16b (SYN)**) S15
(*Z*)-3-(2-(4-Chlorophenyl)-2-oxoethylidene)-7-fluoro-3,4-dihydroquinoxalin-2(1*H*)-one (**17b (ANTI)**) S18
(*Z*)-6-Chloro-3-(2-(4-chlorophenyl)-2-oxoethylidene)-3,4-dihydroquinoxalin-2(1*H*)-one (**16c (SYN)**) S20
(*Z*)-7-Chloro-3-(2-(4-chlorophenyl)-2-oxoethylidene)-3,4-dihydroquinoxalin-2(1*H*)-one (**17c (ANTI)**) S23

(*Z*)-3-(2-(4-Chlorophenyl)-2-oxoethylidene)-2-oxo-1,2,3,4-tetrahydroquinoxaline-6-carboxylic acid (**16d (SYN)**) S26
(*Z*)-2-(2-(4-Chlorophenyl)-2-oxoethylidene)-3-oxo-1,2,3,4-tetrahydroquinoxaline-6-carboxylic acid (**17d (ANTI)**) S30
(*Z*)-3-(2-(4-Chlorophenyl)-2-oxoethylidene)-2-oxo-1,2,3,4-tetrahydroquinoxaline-6-carbonitrile (**16e (SYN)**) S34
(*Z*)-2-(2-(4-Chlorophenyl)-2-oxoethylidene)-3-oxo-1,2,3,4-tetrahydroquinoxaline-6-carbonitrile (**17e (ANTI)**) S38
(*Z*)-3-(2-(4-Chlorophenyl)-2-oxoethylidene)-6-nitro-3,4-dihydroquinoxalin-2(1*H*)-one (**16f (SYN)**) S41
(*Z*)-3-(2-(4-Chlorophenyl)-2-oxoethylidene)-7-nitro-3,4-dihydroquinoxalin-2(1*H*)-one (**17f (ANTI)**) S44

General information

Melting points were measured by Barnstead Electrothermal IA9200 and are uncorrected. ^{1}H and ^{13}C NMR spectra were recorded on Varian Gemini (300 / 600 MHz), chemical shifts are given in parts per million (ppm), tetramethylsilane was used as an internal standard $CDCl_3$ and DMSO-d_6 as the solvent, unless otherwise specified. IR spectra were acquired on FTIR-ATR REACT IR 1000 (ASI Applied Systems) with a diamond probe and MTS detector. Mass spectra were performed on a LC-MS apparatus (Agilent Technologies 1200 Series equipped with Mass spectrometer Agilent Technologies 6100 Quadrupole LC-MS). The course of the reactions was followed by TLC analysis (Merck Silica gel 60 F254). UV lamp (254 nm) and iodine vapours were used for the visualization of TLC spots. Starting chemicals not mentioned in the experimental part were purchased from Sigma-Aldrich, Fluorochem, Alfa Aesar or Acros vendors. Explanations: Ar - argon atmosphere, brine (saturated NaCl solution in water), d - day, EA - ethyl acetate, FLC - flash liquid chromatography, H (Hexol or Petroleum ether) is commercial fraction of hexanes, HV - high vacuum (<0.1 Torr), KGR Büchi - Kugelrohr Glass Oven, RVO - Rotary Vacuum Evaporator.

All prepared compounds are characterized by their M.p., NMR diagrams, NMR and IR textual solutions, their spectra and Elemental analysis. NMR diagrams represent compendious and condensed information about assigned ^{1}H and ^{13}C NMR data to a particular structure. ^{1}H NMR diagrams allow smart check of both chemical shifts and coupling constants for their completeness and correctness. The numbers in the diagrams mean chemical shift in δ ppm and number(s) in parenthesis are coupling constant(s) in Hz. The reason to use NMR diagrams is to read and compare the NMR data more conveniently.

Synthesis of compounds

Graphical abstract for Supporting Information

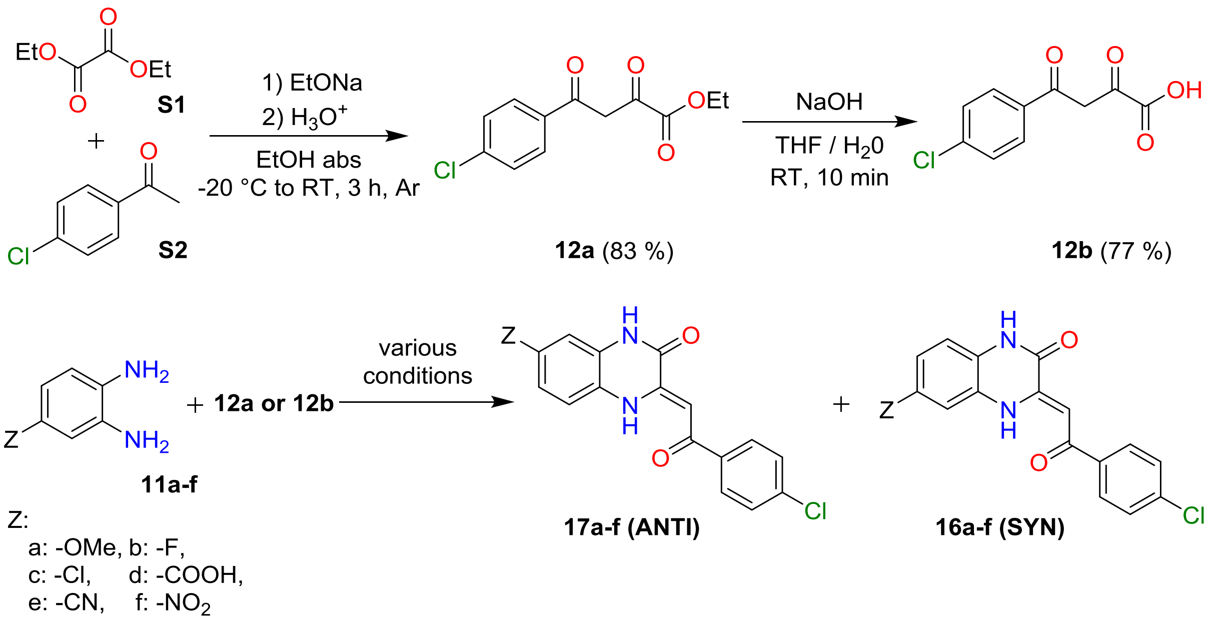

General procedures

General procedure A:

A solution of ethyl 4-chlorobenzoylpyruvate 100 mg (0.39 mmol, 1.00 equiv) **12a**, *o*-phenylenediamine (1.00 equiv) from **11a–f** with or without an additive (1.00 equiv) (*p*-TsOH or DMAP) was stirred in 3.0 ml of DMF (abs) at rt under Ar for 72 h. A low soluble mixture of ANTI/SYN regioisomers slowly precipitated within the reaction. The precipitate was collected by filtration or centrifugation, triturated by 3 ml of Et_2O and crystallized from DMSO (if not otherwise stated) to yield the main solid regioisomer **16** or **17**.

General procedure B:

Diisopropylcarbodiimide 82 µl (66.9 mg, 0.53 mmol, 1.20 equiv) **DIC** was added to a solution of 4-chlorobenzoylpyruvic acid 100 mg (0.44 mmol, 1.00 equiv) **12b** and 73.8 mg (0.53 mmol, 1.20 equiv) of **HOBt** [CAS: 123333-53-9, 97% wetted with ≥ 14 wt % H_2O] in 3.0 ml of DMF (abs) under Ar. The reaction mixture was stirred for 5 min. Then *o*-phenylenediamine (1.00 mol equiv) from **11a–f** was added and the mixture was stirred at rt

under Ar for 72 h. The precipitated product mixture obtained after filtration (or centrifugation) was triturated by 3 ml of Et_2O and crystallized from DMSO (if not otherwise stated) to yield the main solid regioisomer **16** or **17**.

Ethyl 4-chlorobenzoylpyruvate (12a)

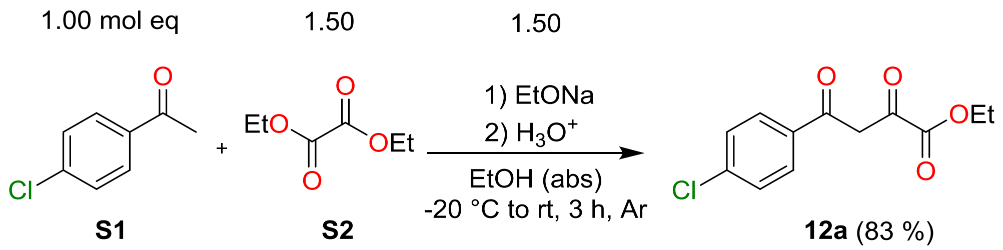

The ester **12a** was prepared according to the procedure described in the literature[1] with 71% yield.

Novelty: Compound **12a** was previously described in the literature with its M.p., ^{1}H NMR and ^{13}C NMR spectrum.[1]

M.p.: 62.0 - 63.0 °C [EtOH], yellow solid compound (lit. 62 - 63 °C [EtOH]).[1]

NMR diagrams:

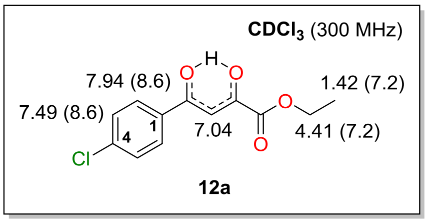

^{1}H-NMR (300 MHz, $CDCl_3$): δ 7.94 (d, 2H, J(2,3) = 8.6 Hz, 2 x H-C(2)), 7.49 (d, 2H, J(2,3) = 8.6 Hz, 2 x H-C(3)), 7.04 (s, 1H, -CH=), 4.41 (q, 2H, $J(CH_2,CH_3)$ = 7.2 Hz, $-CH_2-$), 1.42 (t,

[1] Geffken, D.; Soliman, R.; Soliman, F.S.D.; Abdel-Khalek, M.M.; Issa, A.E. *Med. Chem. Res.* **2011**, *20*, 408-420.

3H, J(CH_2,CH_3) = 7.2 Hz, -CH_3). Enolic hydroxy group has chemical shift out of measured range.

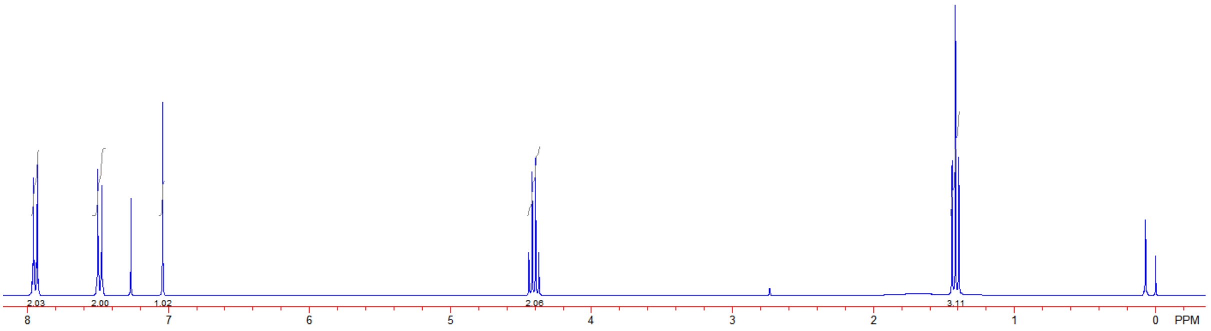

Figure S1. ^{1}H-NMR (300 MHz, $CDCl_3$), spectrum of compound **12a**.

FT IR (solid, cm^{-1}): 3413 (s, OH), 2986 (m), 1727 (m, C=O), 1718 (m, C=O), 1588 (s, C=O), 1479 (m), 1447 (w), 1397 (w), 1366 (m), 1265 (s), 1175 (m), 1135 (w), 1106 (m), 1088 (s), 1007 (s), 935 (w), 857 (m), 831 (m), 778 (m), 766 (s), 667 (m), 628 (m).

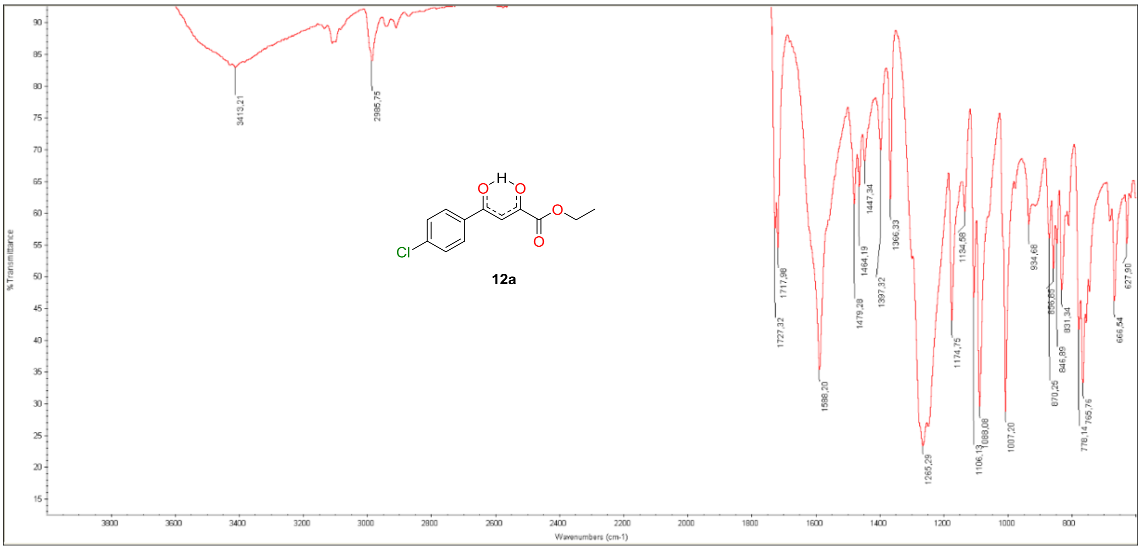

Figure S2. IR spectrum of compound **12a**.

MS (ESI m/z): 253.2 $[M-H]^-$

Anal. calcd for $C_{12}H_{11}ClO_4$ (254.67): C, 56.59; H, 4.35; Cl, 13.92. Found: C, 56.78; H, 4.55; Cl, 13.74.

4-Chlorobenzoylpyruvic acid (12b)

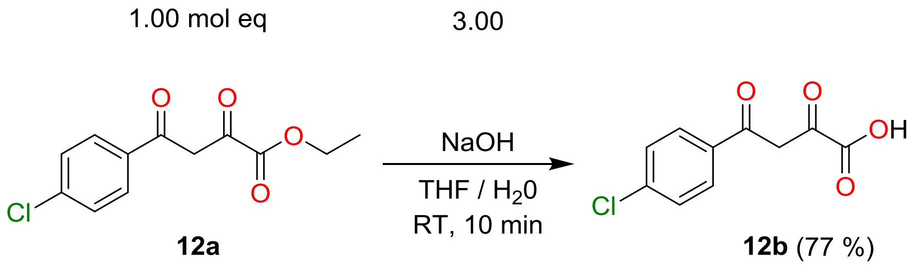

The acid **12b** was prepared according to the procedure described in the literature.[2] The reaction time was shortened to 10 minutes due to observed 4-chloroacetophenone formation via retro-claisen reaction.

Novelty: Compound **12b** was described in the literature by M.p.[3] and ^{1}H-NMR[4] spectrum.

M.p.: 163.0 - 165.0 °C [H_2O], white solid compound (lit. 163 - 165 °C [H_2O]).[3]

NMR diagrams:

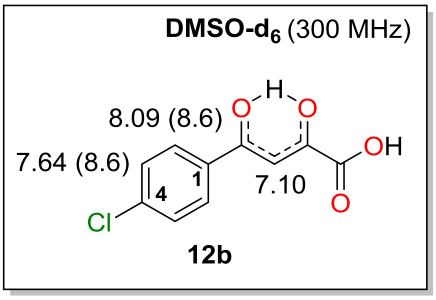

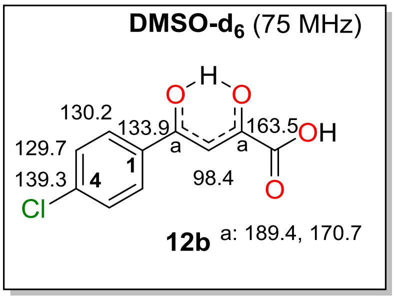

---

[2] Tumey, L.N.; Huck, B.; Gleason, E.; Wang, J.; Silver, D.; Brunden, K.; Boozer, S.; Rundlett, S.; Sherf, B.; Murphy, S.; Bailey, A.; Dent, T.; Leventhal, Ch.; Harrington, J.; Bennani, Y.L. *Bioorg. Med. Chem. Lett.* **2004**, *14*, 4915-4918.
[3] Andreichikov et al. *Zh. Org. Khim.* **1978**, *14*, 338-371.
[4] Sofina, O. A.; Igidov, N. M.; Kozminykh, E. N.; Trapeznikova, N. N.; Kasatkina, Yu. S.; Kozminykh, V. O. *Russ. J. Org. Chem.* **2001**, *37*, 1017-1025.

^{1}H NMR (300 MHz, DMSO-d_6): δ 8.09 (d, 2H, J(2,3) = 8.6 Hz, 2 x H-C(2)), 7.64 (d, 2H, J(2,3) = 8.6 Hz, 2 x H-C(3)), 7.10 (s, 1H, -CH=), -OH and -COOH not seen.

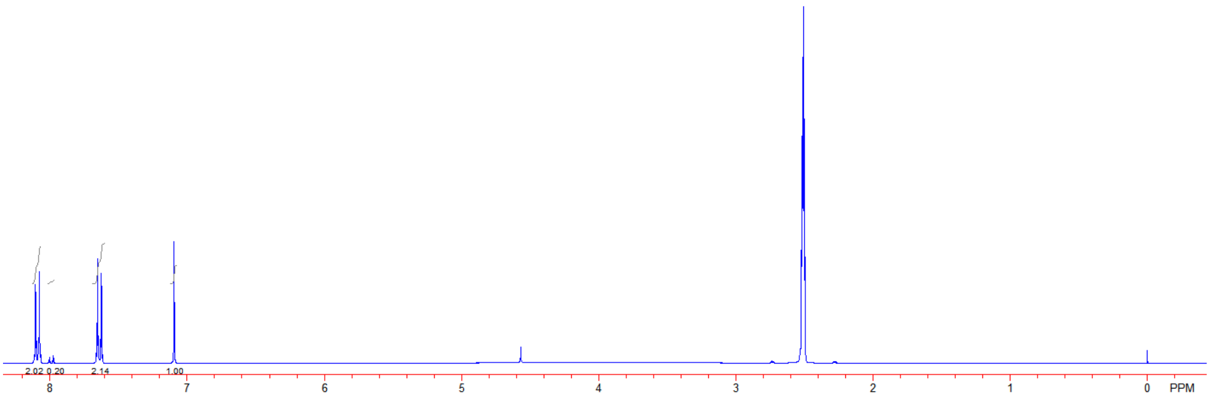

Figure S3 ^{1}H NMR (300 MHz, DMSO-d_6), spectrum of compound **12b**.

^{13}C NMR (75 MHz, DMSO-d_6): δ 189.4 and 170.7 (β-diketo carbonyls), 163.5 (-COOH), 139.3 (C(4)), 133.9 (C(1)), 130.2 (2 x C(2)), 129.7 (2 x C(3)), 98.4 (-CH=).

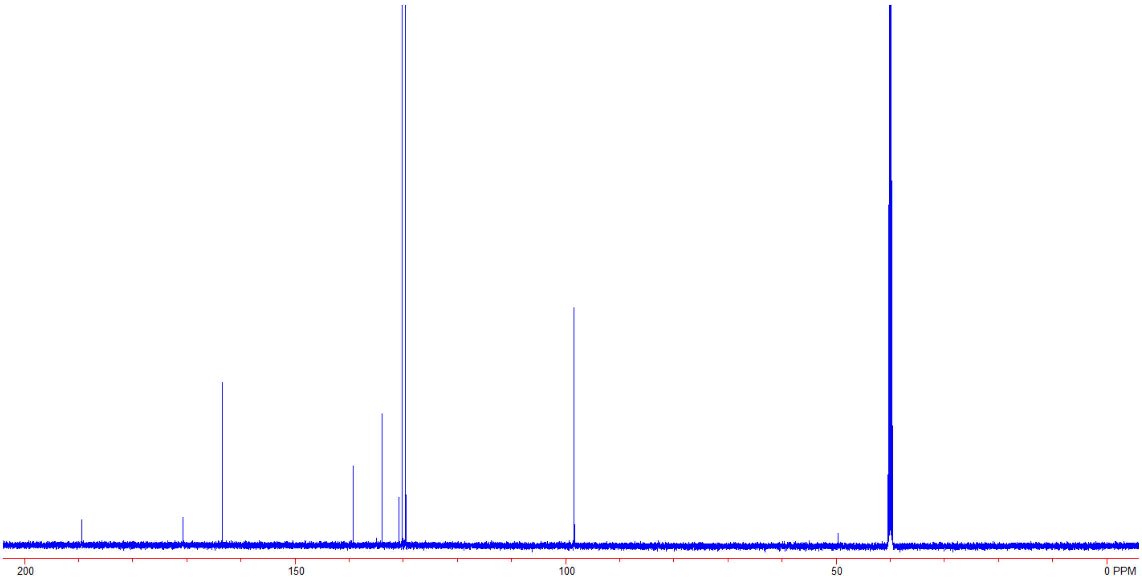

Figure S4. ^{13}C-NMR (75 MHz, DMSO-d_6), spectrum of compound **12b**.

FTIR (solid, cm^{-1}): 3501 (s, OH), 1923 (w), 1624 (s, C=O), 1582 (s, C=O), 1492 (m), 1455 (m), 1402 (m), 1319 (m), 1283 (m), 1234 (s), 1187 (m), 1142 (s), 1112 (m), 1095 (s), 1056 (m), 1012 (m), 923 (w), 850 (m), 829 (m), 815 (m), 777 (s), 743 (m), 667 (m), 627 (m).

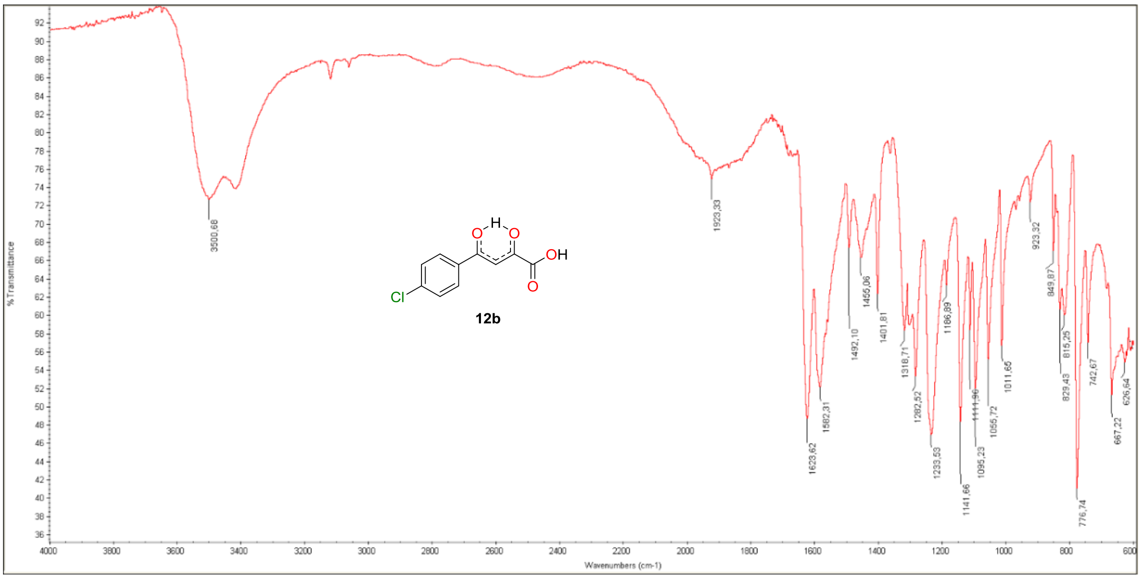

Figure S5. IR spectrum of compound **12b**

MS (ESI m/z): 225.0 [M-H]$^{-}$

Anal. calcd for $C_{10}H_7ClO_4$ (226.61): C, 53.00; H, 3.11. Found: C, 53.09; H, 3.12.

(*Z*)-3-(2-(4-Chlorophenyl)-2-oxoethylidene)-6-methoxy-3,4-dihydroquinoxalin-2(1*H*)-one (16a (SYN))

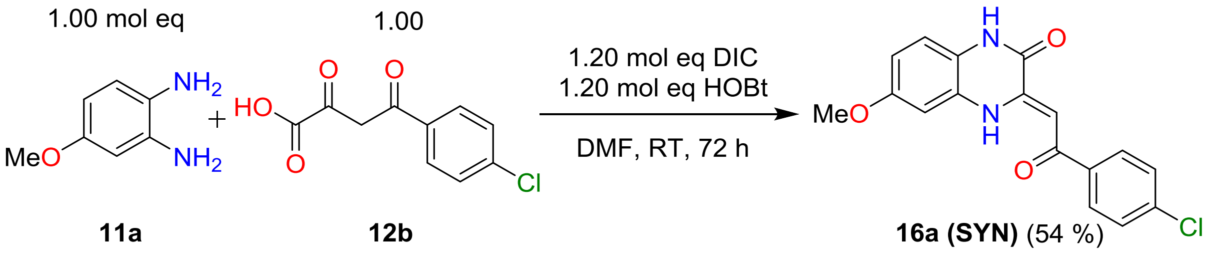

The 3,4-dihydroquinoxaline-2(1*H*)-one **16a (SYN)** was prepared according to the general procedure B from acid **12b** diamine **11a**. The crude mixture of ANTI / SYN regioisomers was purified by trituration with boiling ethyl acetate yielding 78.4 mg (0.24 mmol, 54%) of **16a (SYN)**.

Novelty: Compound **16a (SYN)** was not described in the literature.

M.p.: 269.0 - 272.0 °C [EA], brown solid compound.

NMR diagrams:

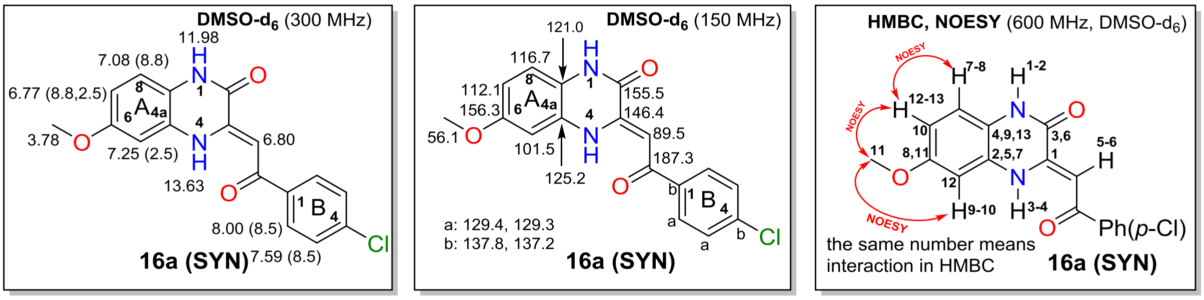

^{1}H NMR (300 MHz, DMSO-*d*$_6$): δ 13.63 (s, 1H, H-N$_A$(4)), 11.98 (s, 1H, H-N$_A$(1)), 8.00 (d, 2H, J(B$_2$,B$_3$) = 8.5 Hz, 2 x H-C$_B$(2)), 7.59 (d, 2H, J(B$_2$,B$_3$) = 8.5 Hz, 2 x H-C$_B$(3)), 7.25 (d, 1H, J(A$_5$,A$_7$) = 2.5 Hz, H-C$_A$(5)), 7.08 (d, 1H, J(A$_7$,A$_8$) = 8.8 Hz, H-C$_A$(8)), 6.80 (s, 1H, -COCH=), 6.77 (dd, 1H, J(A$_7$,A$_8$) = 8.8 Hz, J(A$_5$,A$_7$) = 2.5 Hz, H-C$_A$(7)), 3.78 (s, 3H, -OMe) .

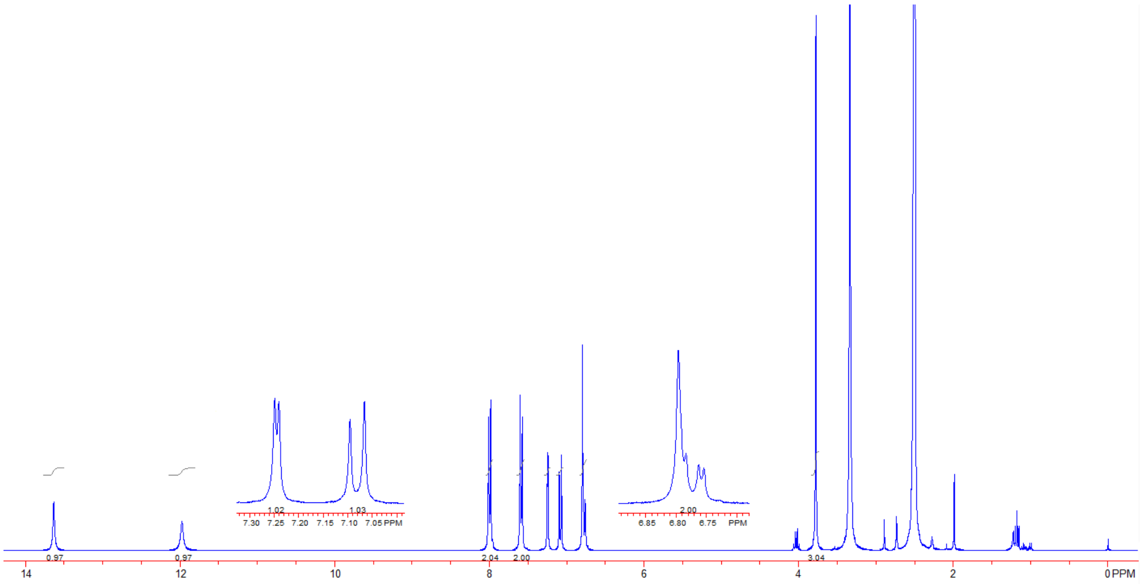

Figure S6. ^{1}H-NMR (300 MHz, DMSO-*d*$_6$) spectrum of compound **16a (SYN)**.

^{13}C NMR (150 MHz, DMSO-d_6): δ 187.3 (C_B(1)<u>C</u>=O), 156.3 (C_A(6)), 155.5 (C_A(2)=O), 146.4 (C_A(3)), 137.8 and 137.2 (C_B(1 and 4)), 129.4 and 129.3 (2 x C_B(2 and 3)), 125.2 (C_A(4a)), 121.0 (C_A(8a)), 116.7 (C_A(8)), 112.1 (C_A(7)), 101.5 (C_A(5)), 89.5 (-CO<u>C</u>H=), 56.1 (-OCH_3).

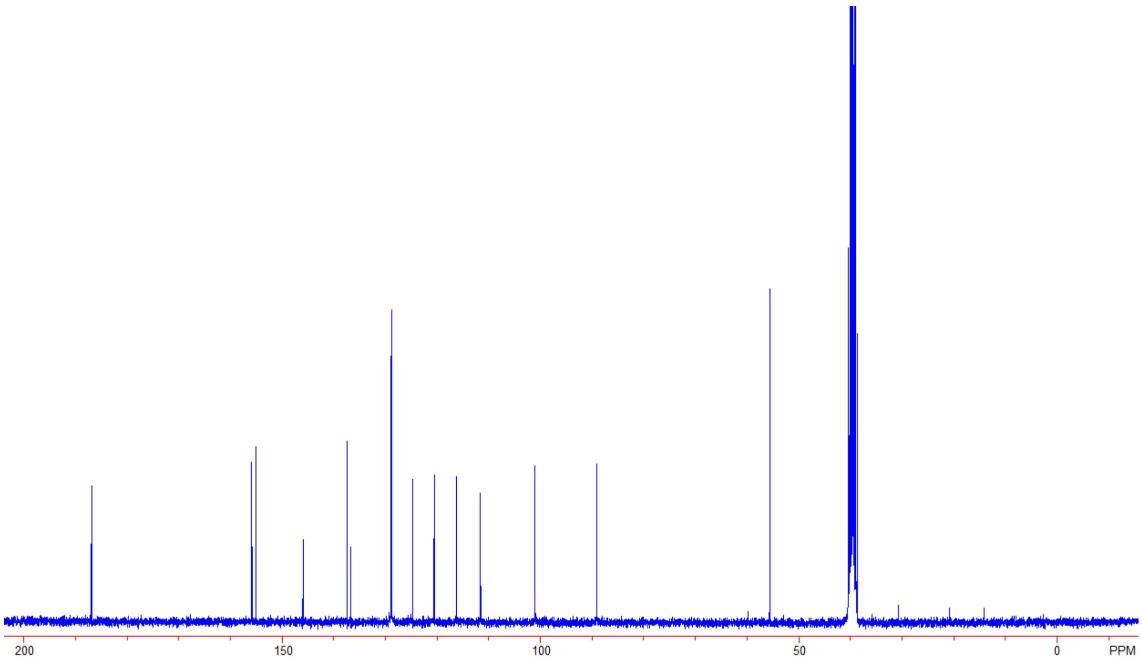

Figure S7. ^{13}C NMR (150 MHz, DMSO-d_6) spectrum of compound **16a (SYN)**.

FTIR (solid, cm^{-1}): 3063 (w, NH), 1741 (w), 1674 (s, C=O), 1600 (s, C=O), 1576 (s), 1526 (s), 1500 (s), 1488 (m), 1456 (m), 1413 (m), 1361 (s), 1304 (m), 1254 (s), 1182 (m), 1161 (m), 1093 (m), 1036 (m), 1012 (m), 973 (w), 867 (m), 790 (s), 751 (s), 657 (m), 621 (w).

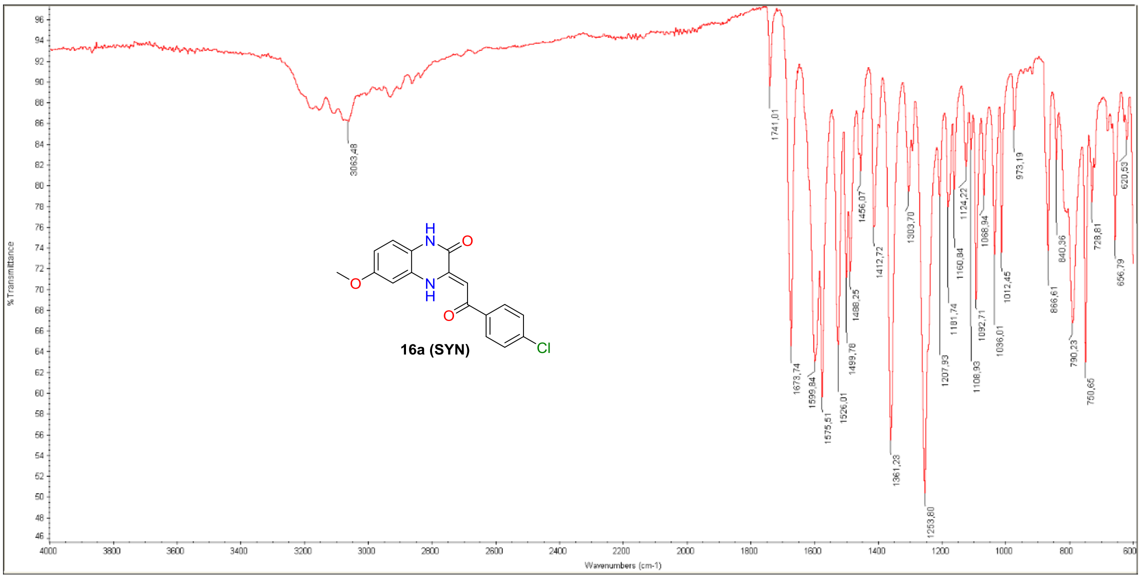

Figure S8. IR spectrum of compound **16a (SYN)**.

MS (ESI m/z): 327.1 [M-H]$^-$.

Anal. calcd for $C_{17}H_{13}ClN_2O_3$ (328.75): C, 62.11; H, 3.99; N, 8.52. Found: C, 62.05; H, 4.07; N, 8.48.

(*Z*)-3-(2-(4-Chlorophenyl)-2-oxoethylidene)-7-methoxy-3,4-dihydroquinoxalin-2(1*H*)-one (17a (ANTI))

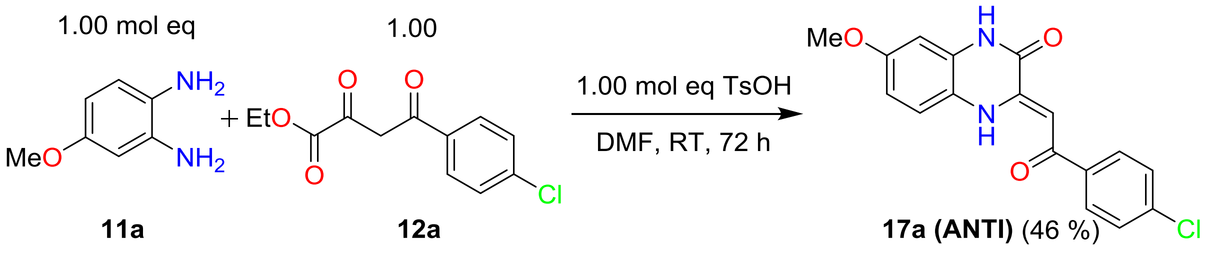

The 3,4-Dihydroquinoxaline-2(1*H*)-one **17a (ANTI)** was prepared according to the general procedure A from ester **12a** and diamine **11a** with *p*-TsOH as additive. The crude mixture of ANTI / SYN regioisomers was purified by trituration with acetone and crystalized from EA yielding 59.2 mg (0.18 mmol, 46%) of **17a (ANTI)**.

Novelty: Compound **17a (ANTI)** was not described in the literature.

M.p.: 288.0 - 291.0 °C [EA], yellow solid compound.

NMR diagrams:

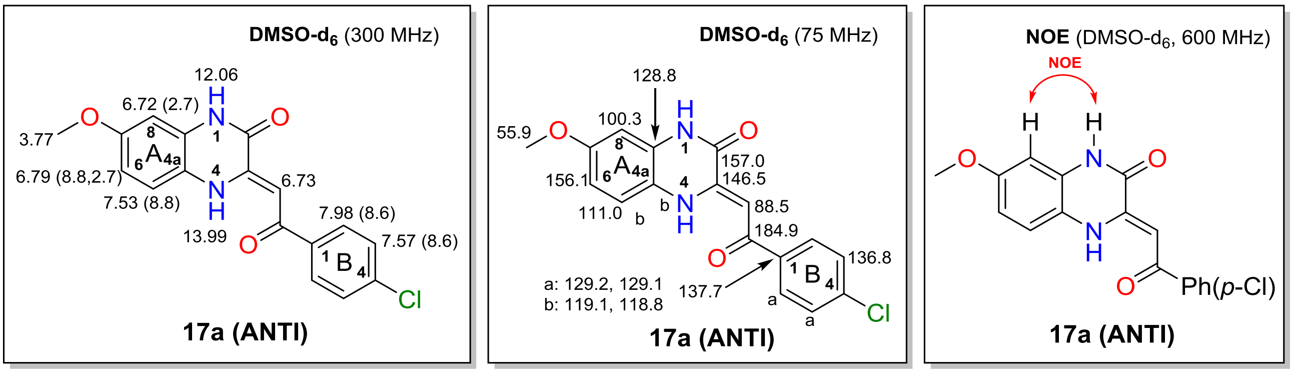

¹H-NMR (300 MHz, DMSO-d$_6$): δ 13.99 (s, 1H, H-N$_A$(4)), 12.06 (s, 1H, H-N$_A$(1)), 7.98 (d, 2H, J(B$_2$,B$_3$) = 8.6 Hz, 2 x H-C$_B$(2)), 7.57 (d, 2H, J(B$_2$,B$_3$) = 8.6 Hz, 2 x H-C$_B$(3)), 7.53 (d, 1H, J(A$_5$,A$_6$) = 8.8 Hz, H-C$_A$(5)), 6.79 (dd, 1H, J(A$_5$,A$_6$) = 8.8 Hz, J(A$_6$,A$_8$) = 2.7 Hz, Hz, H-C$_A$(6)), 6.73 (s, 1H, -COCH=), 6.72 (d, 1H, J(A$_6$,A$_8$) = 2.7 Hz, H-C$_A$(8)), 3.77 (s, 3H, -OCH$_3$).

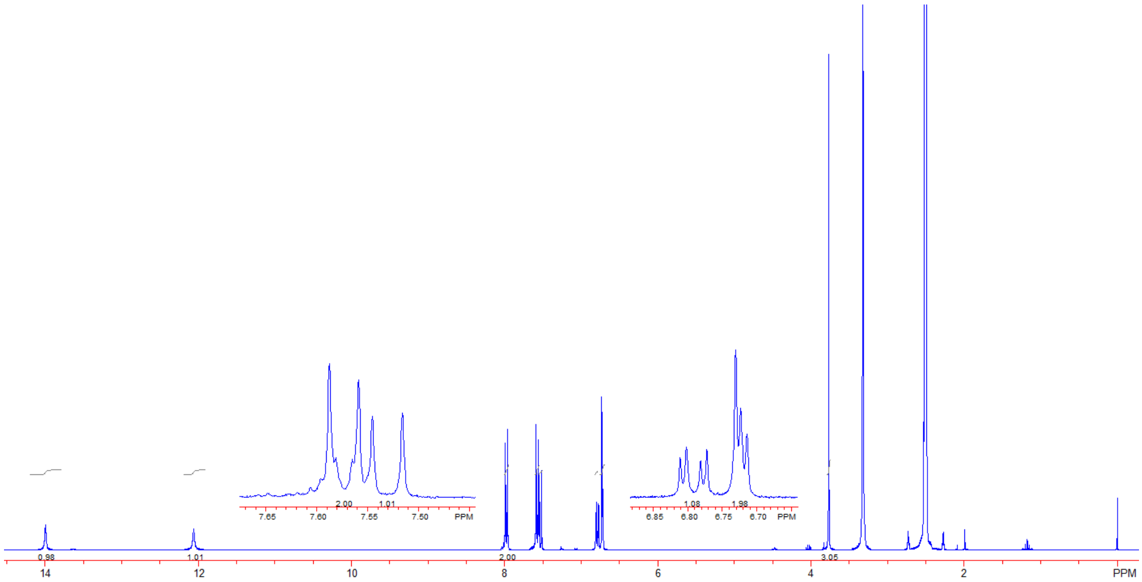

Figure S9. ^{1}H-NMR (300 MHz, DMSO-d_6) spectrum of compound **17a (ANTI)**.

^{13}C-NMR (75 MHz, DMSO-d_6): δ 184.9 ($C_B(1)\underline{C}$=O), 157.0 ($C_A(2)$=O), 156.1 ($C_A(7)$), 146.5 ($C_A(3)$), 137.7 ($C_B(1)$), 136.8 ($C_B(4)$), 129.2 and 129.1 (2 x C_B(2 and 3)), 128.8 ($C_A(8a)$), 119.1 and 118.8 ($C_A(4a)$ and $C_A(5)$), 111.0 ($C_A(6)$), 100.3 ($C_A(8)$), 88.5 (-CO$\underline{C}$H=), 55.9 (-O$\underline{C}H_3$).

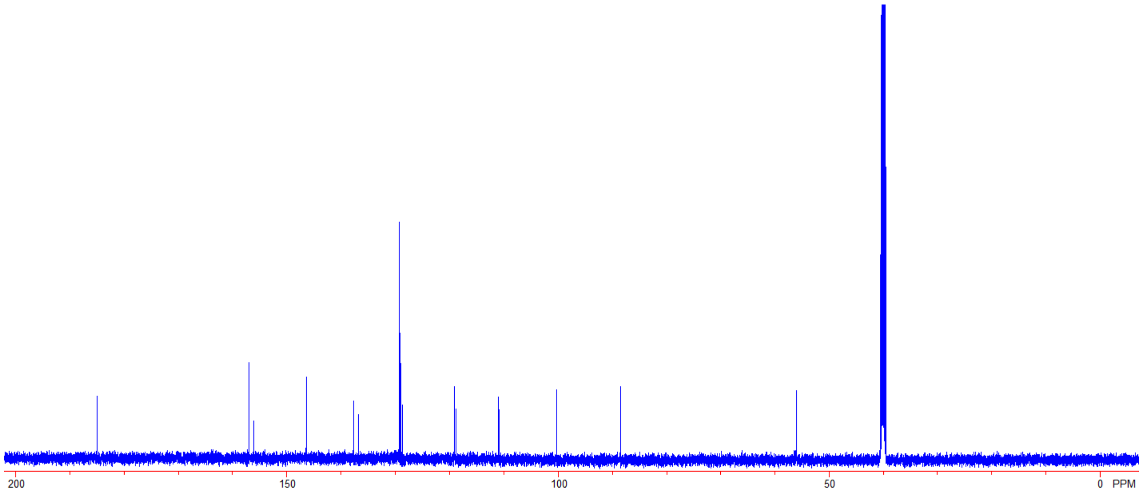

Figure S10. ^{13}C-NMR (75 MHz, DMSO-d_6) spectrum of compound **17a (ANTI)**.

FTIR (solid, cm^{-1}): 3001 (w, NH), 2837 (w, NH), 1668 (s, C=O), 1632 (s, C=O), 1623 (s), 1524 (m), 1460 (m), 1399 (w), 1358 (m), 1267 (m), 1204 (m), 1170 (m), 1152 (m), 1087 (m), 961 (w), 842 (m), 797 (s), 787 (s), 718 (s), 753 (s), 688 (m), 667 (m), 613 (m).

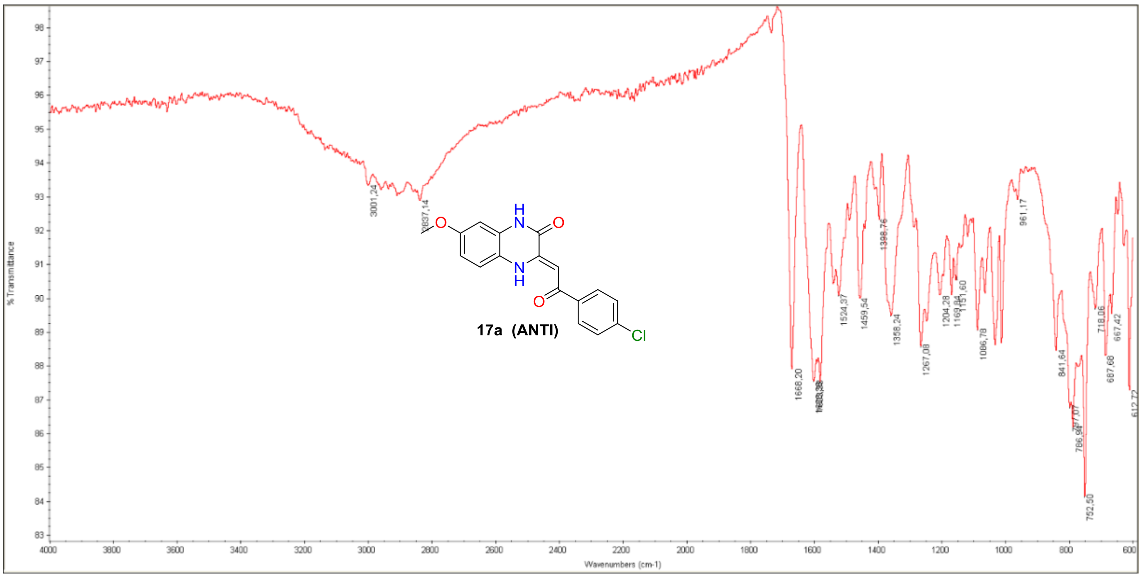

Figure S11. IR spectrum of compound **17a (ANTI)**.

MS (ESI m/z): 327.1 [M-H]$^{-}$.

Anal. calcd for $C_{17}H_{13}ClN_2O_3$ (328.75): C, 62.11; H, 3.99; N, 8.52. Found: C, 62.07; H, 4.06; N, 8.36.

(*Z*)-3-(2-(4-Chlorophenyl)-2-oxoethylidene)-6-fluoro-3,4-dihydroquinoxalin-2(1*H*)-one (16b (SYN))

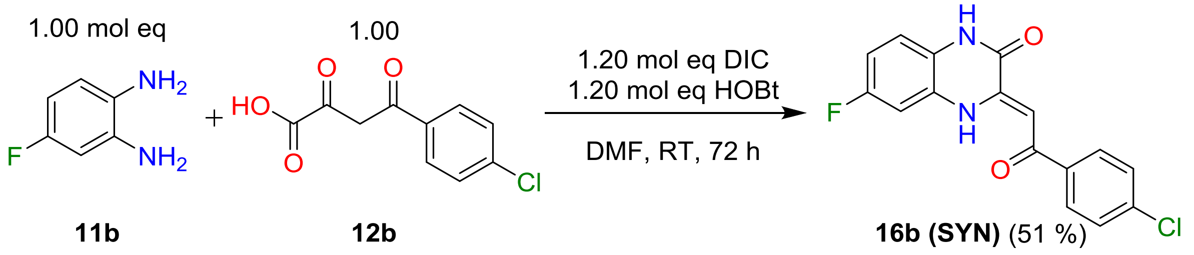

The 3,4-Dihydroquinoxaline-2(1*H*)-one **16b (SYN)** was prepared according to the general procedure B from acid **12b** diamine **11b**. The crude mixture of ANTI / SYN regioisomers was purified by crystallization from DMSO yielding 71.3 mg (0.23 mmol, 51%) of **16b (SYN)**.

Novelty: Compound **16b (SYN)** was not described in the literature.

M.p.: 310.0 - 314.0 °C [DMSO], yellow solid compound.

NMR diagrams:

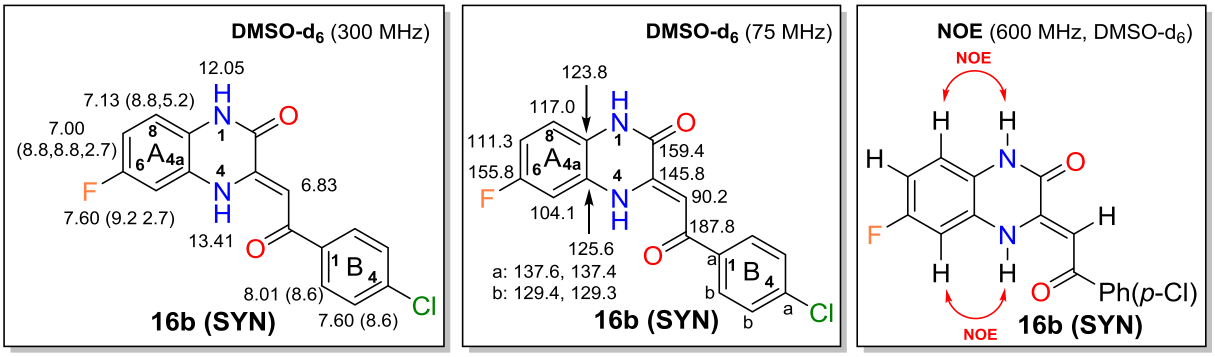

^{1}H NMR (300 MHz, DMSO-d_6): δ 13.41 (s, 1H, H-N$_A$(4)), 12.05 (s, 1H, H-N$_A$(1)), 8.01 (d, 2H, J(B$_2$,B$_3$) = 8.6 Hz, 2 x H-C$_B$(2)), 7.60 (d, 2H, J(B$_2$,B$_3$) = 8.6 Hz, 2 x H-C$_B$(3)), 7.60 (dd, 1H, J(A$_5$,F) = 9.2 Hz, J(A$_5$,A$_7$) = 2.7 Hz, H-C$_A$(5)), 7.13 (dd, 1H, J(A$_7$,A$_8$) = 8.8 Hz, J(A$_8$,F)

= 5.2 Hz, H-$C_A(8)$), 7.00 (ddd, 1H, $J(A_7,A_8)$ = 8.8 Hz, $J(A_7,F)$ = 8.8 Hz, $J(A_5,A_7)$ = 2.7 Hz, H-$C_A(7)$), 6.83 (s, 1H, -COCH=).

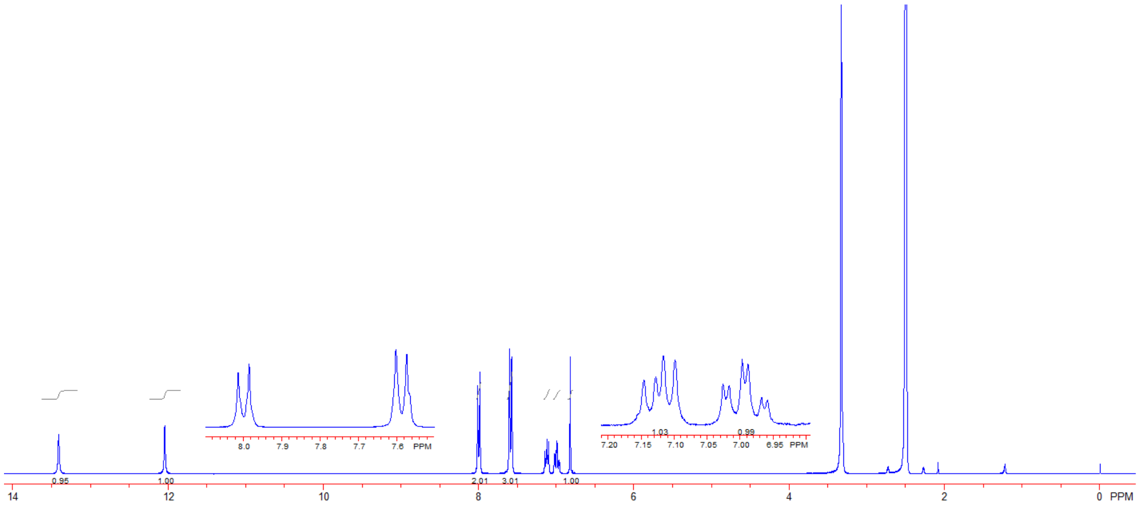

Figure S12. ^{1}H NMR (300 MHz, DMSO-d_6) spectrum of compound **16b (SYN)**.

^{13}C NMR (75 MHz, DMSO-d_6): δ 187.8 ($C_B(1)\underline{C}$=O), 159.4 ($C_A(2)$=O), 155.8 ($C_A(6)$), 145.8 ($C_A(3)$), 137.6 and 137.4 ($C_B(1)$ and $C_B(4)$), 129.4 and 129.3 (2 x C_B(2 and 3)), 125.6 ($C_A(4a)$), 123.8 ($C_A(8a)$), 117.0 ($C_A(8)$), 111.3 ($C_A(7)$), 90.2 (-CO$\underline{C}$H=).

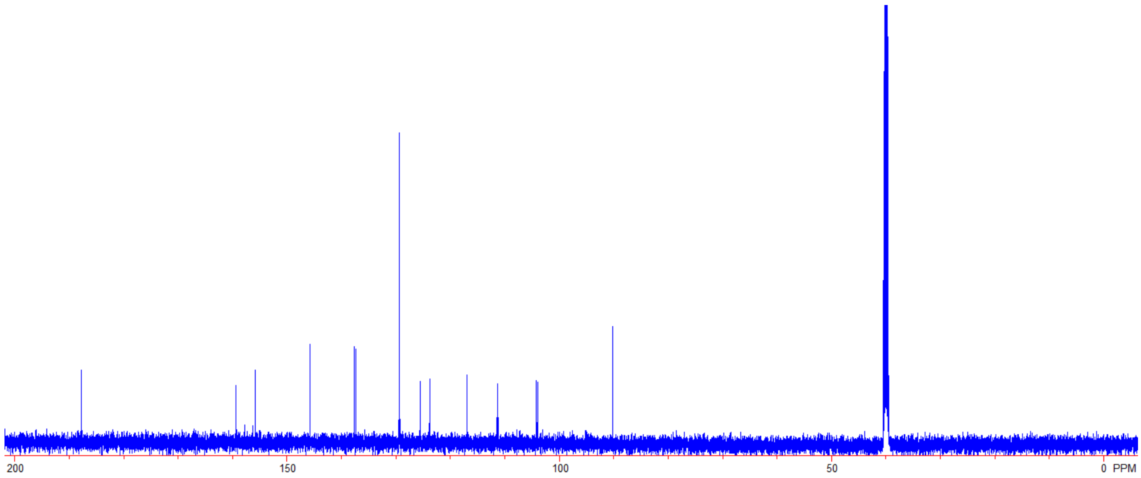

Figure S13. ^{13}C NMR (75 MHz, DMSO-d_6) spectrum of compound **16b (SYN)**.

FTIR (solid, cm^{-1}): 3080 (s, NH), 1684 (s, C=O), 1605 (m, C=O), 1588 (m), 1540 (m), 1523 (s), 1500 (m), 1486 (m), 1456 (m), 1424 (m), 1397 (m), 1362 (m), 1322 (w), 1278 (w), 1251 (s), 1238 (s), 1175 (m), 1151 (m), 1115 (m), 1091 (s), 1070 (m), 1012 (m), 984 (w), 915 (w), 880 (m), 870 (m), 842 (m), 794 (s), 780 (s), 751 (m), 719 (m), 668 (s), 629 (m).

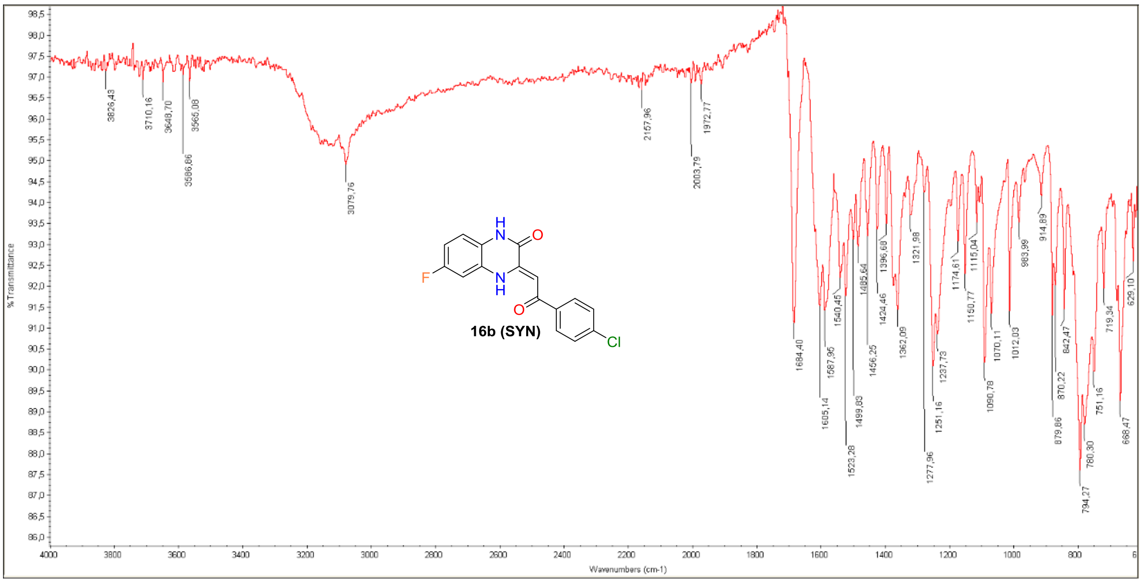

Figure S14. IR spectrum of compound **16b (SYN)**.

MS (ESI m/z): 315.1 $[M-H]^-$.

Anal. calcd for $C_{16}H_{10}ClFN_2O_2$ (316.71): C, 60.68; H, 3.18; N, 8.85. Found: C, 60.89; H, 3.33; N, 8.90.

(*Z*)-3-(2-(4-Chlorophenyl)-2-oxoethylidene)-7-fluoro-3,4-dihydroquinoxalin-2(1*H*)-one (17b (ANTI))

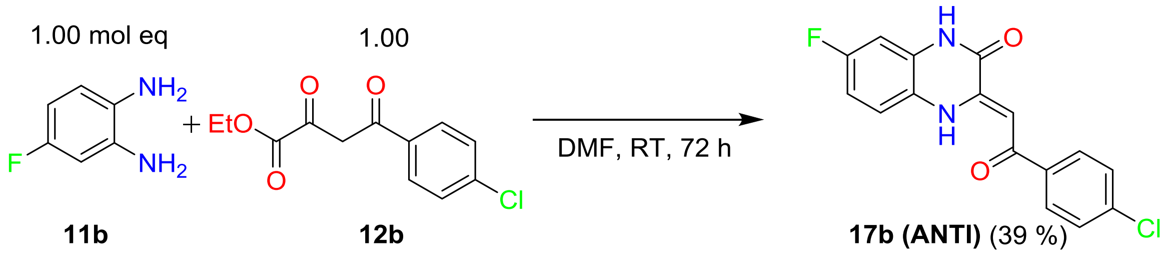

The 3,4-dihydroquinoxaline-2(1*H*)-one **17b (ANTI)** was prepared according to the general procedure A from ester **12a** and diamine **11b** without any additive. The crude mixture of ANTI / SYN regioisomers was purified by FLC (EA / H, 1 / 3) yielding 48.5 mg (0.15 mmol, 39%) of **17b (ANTI)**.

Novelty: Compound **17b (ANTI)** was not described in the literature.

M.p.: 301.0 - 305.0 °C [EA / H], yellow solid compound.

NMR diagrams:

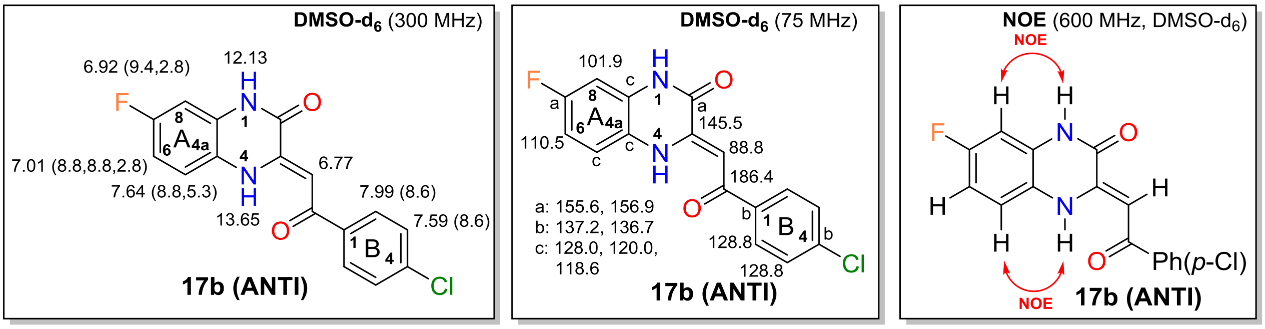

^{1}**H NMR** (300 MHz, DMSO-d_6): δ 13.65 (s, 1H, H-N$_A$(4)), 12.13 (s, 1H, H-N$_A$(1)), 7.99 (d, 2H, J(B$_2$,B$_3$) = 8.6 Hz, 2 x H-C$_B$(2)), 7.64 (dd, 1H, J(A$_5$,A$_6$) = 8.8 Hz, J(A$_5$,F) = 5.3 Hz, H-C$_A$(5)), 7.59 (d, 2H, J(B$_2$,B$_3$) = 8.6 Hz, 2 x H-C$_B$(3)), 7.01 (ddd, 1H, J(A$_5$,A$_6$) = 8.8 Hz,

J(A$_6$,F) = 8.8 Hz, J(A$_6$,A$_8$) = 2.8 Hz, H-C$_A$(6)), 6.92 (dd, 1H, J(A$_8$,F)) = 9.4 Hz, J(A$_6$,A$_8$) = 2.8 Hz, H-C$_A$(8)), 6.77 (s, 1H, -COCH=).

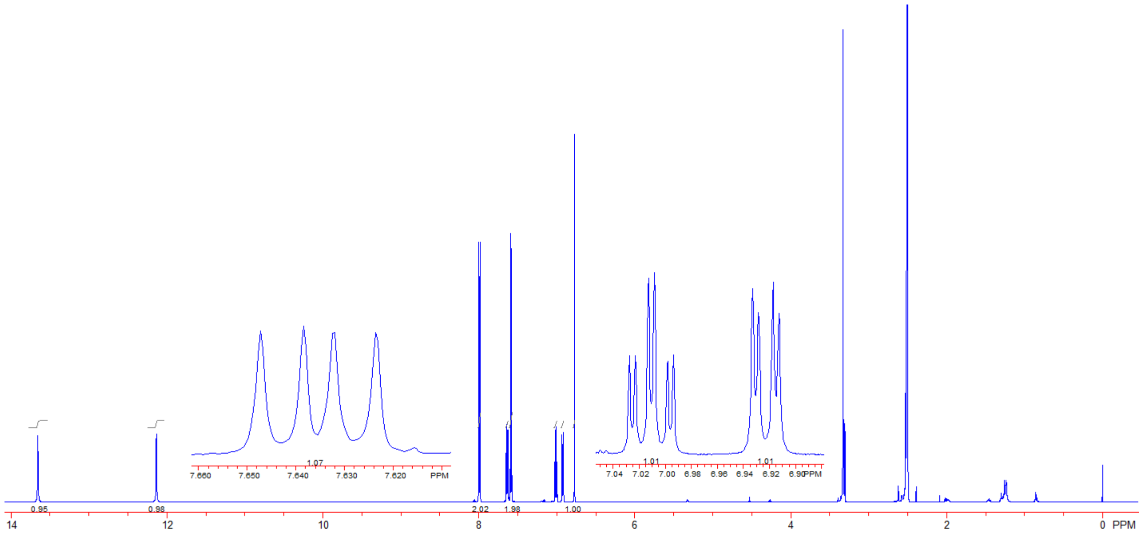

Figure S15. ^{1}H NMR (300 MHz, DMSO-d_6) spectrum of compound **17b (ANTI)**.

^{13}C NMR (75 MHz, DMSO-d_6): δ 186.4 (C$_B$(1)<u>C</u>=O), 156.9 and 155.6 (C$_A$(2)=O and C$_A$(7)), 145.5 (C$_A$(3)), 137.2 and 136.7 (C$_B$(1 and 4)), 2 x 128.8 (2 x C$_B$(2 and 3)), 128.0, 120.0 and 118.6 (C$_A$(5, 4a and 8a)), 110.5 (C$_A$(6)), 101.9 (C$_A$(8)), 88.8 (-CO<u>C</u>H=).

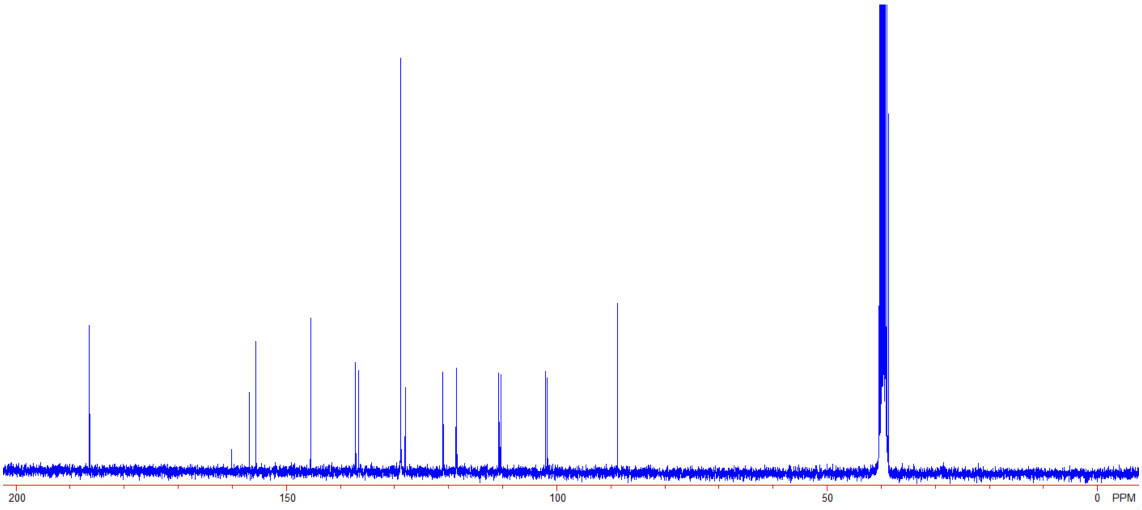

Figure S16. ^{13}C NMR (75 MHz, DMSO-d_6) spectrum of compound **17b (ANTI)**.

FTIR (solid, cm^{-1}): 2962 (s, NH), 1684 (w, C=O), 1617 (w, C=O), 1515 (w), 1368 (w), 1257 (m), 1010 (s), 788 (s), 752 (m), 680 (m).

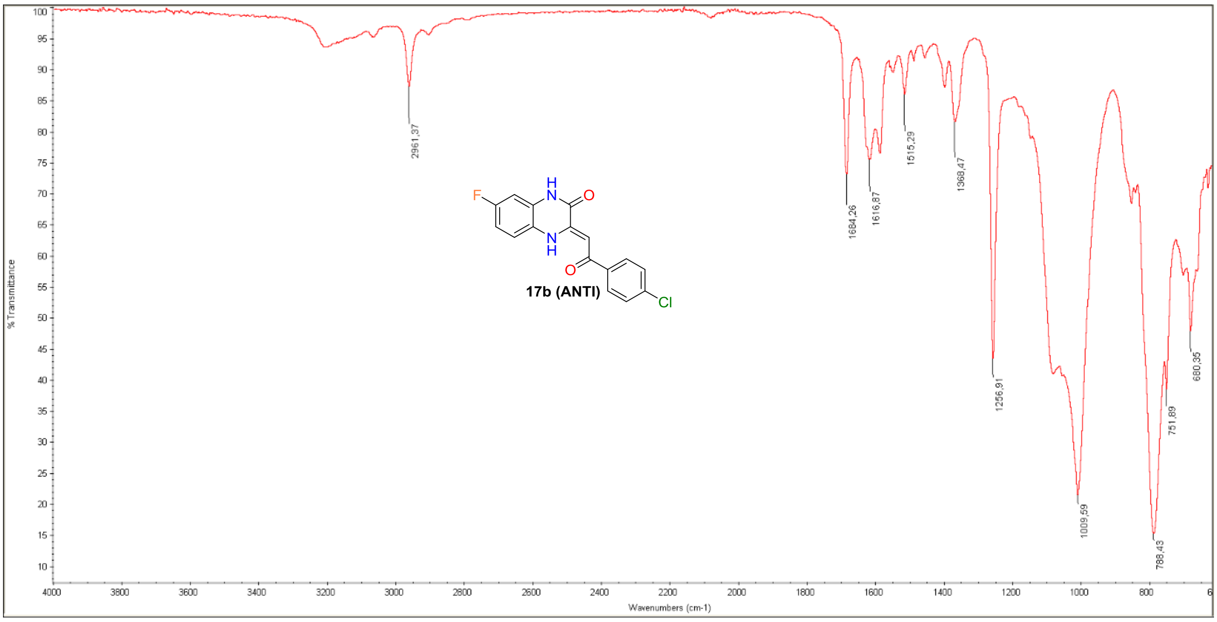

Figure S17. IR spectrum of compound **17b (ANTI)**.

MS (ESI m/z): 315.0 [M-H]$^-$.

Anal. calcd for $C_{16}H_{10}ClFN_2O_2$ (316.71): C, 60.68; H, 3.18; N, 8.85. Found: C, 60.50; H, 3.22; N, 8.71.

(*Z*)-6-Chloro-3-(2-(4-chlorophenyl)-2-oxoethylidene)-3,4-dihydroquinoxalin-2(1*H*)-one (16c (SYN))

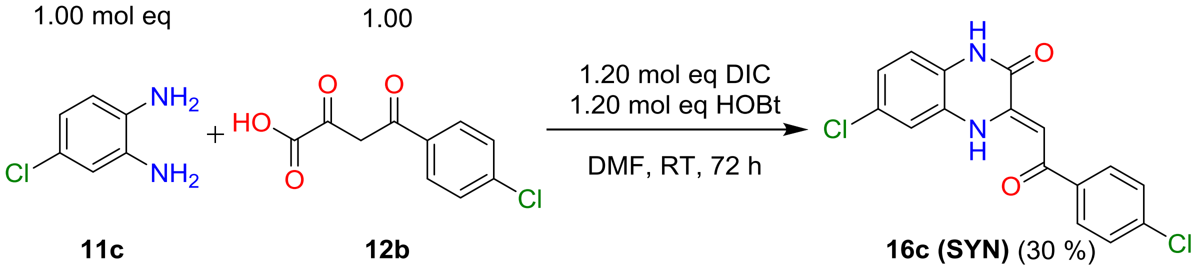

The 3,4-dihydroquinoxaline-2(1*H*)-one **16c (SYN)** was prepared according to the general procedure B from acid **12b** diamine **11c**. The crude mixture of ANTI / SYN regioisomers was purified by precipitation from DMSO by H_2O yielding 44.1 mg (0.13 mmol, 30%) of **16c (SYN)**.

Novelty: Compound **16c (SYN)** was not described in the literature.

M.p.: 285.4 – 286.8 °C [DMSO], yellow solid compound.

NMR diagrams:

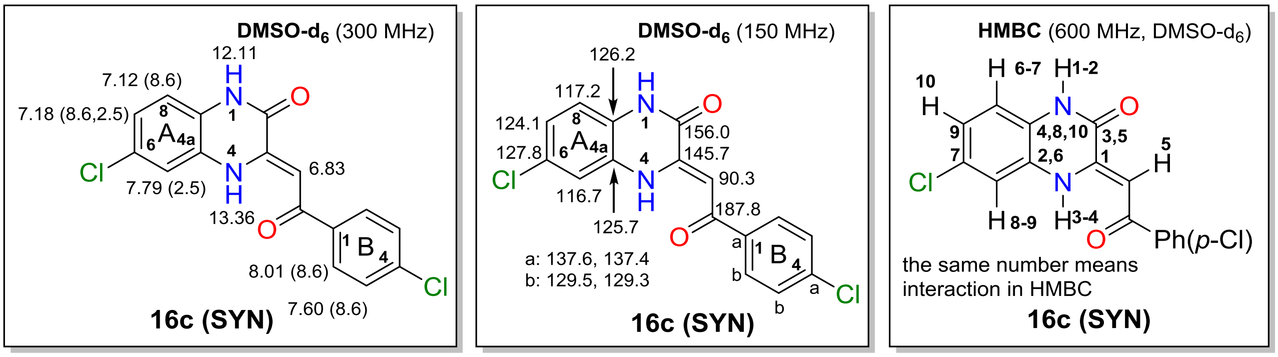

^{1}H NMR (300 MHz, DMSO-*d*$_6$): δ 13.36 (s, 1H, H-N$_A$(4)), 12.11 (s, 1H, H-N$_A$(1)), 8.01 (d, 2H, J(B$_2$,B$_3$) = 8.6 Hz, 2 x H-C$_B$(2)), 7.79 (d, 1H, J(A$_5$,A$_7$) = 2.5 Hz, H-C$_A$(5)), 7.60 (d, 2H, J(B$_2$,B$_3$) = 8.6 Hz, 2 x H-C$_B$(3)), 7.18 (dd, 1H, J(A$_7$,A$_8$) = 8.6 Hz, J(A$_5$,A$_7$) = 2.5 Hz, H-C$_A$(7)), 7.12 (d, 1H, J(A$_7$,A$_8$) = 8.6 Hz, H-C$_A$(8)), 6.83 (s, 1H, -COCH=).

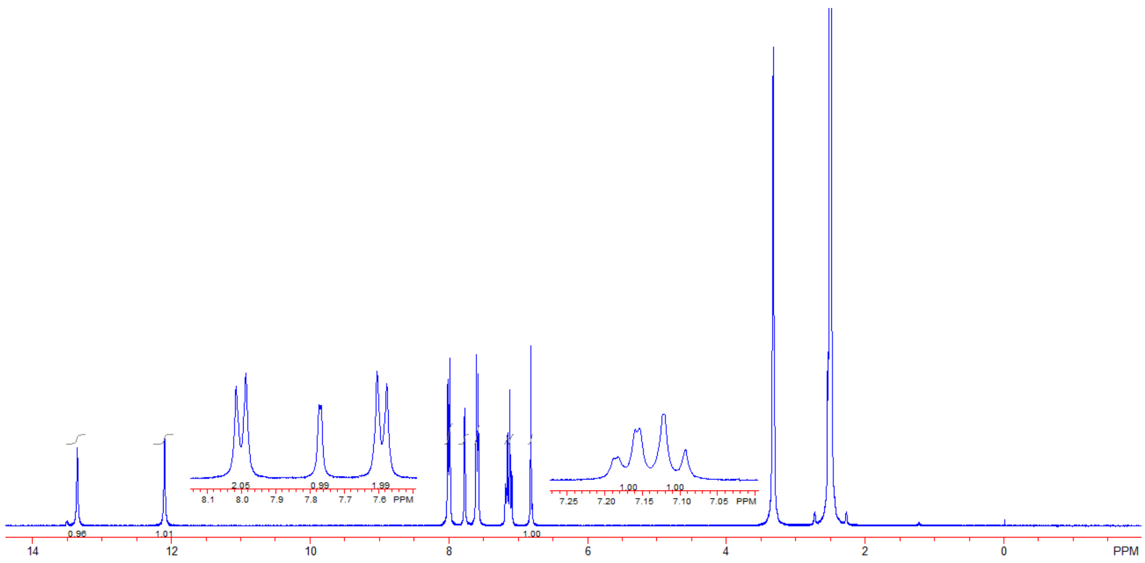

Figure S18. ^{1}H NMR (300 MHz, DMSO-d_6) spectrum of compound **16c (SYN)**.

^{13}C NMR (150 MHz, DMSO-d_6): δ 187.8 (C_B(1)<u>C</u>=O), 156.0 (C_A(2)=O), 145.7 (C_A(3)), 137.6 and 137.4 (C_B(1 and 4)), 129.5 and 129.3 (2 x C_B(2 and 3)), 127.8 (C_A(6)), 126.1 (C_A(8a)), 125.8 (C_A(4a)), 124.1 (C_A(7)), 117.1 (C_A(8)), 116.7 (C_A(5)), 90.3 (-CO<u>C</u>H=).

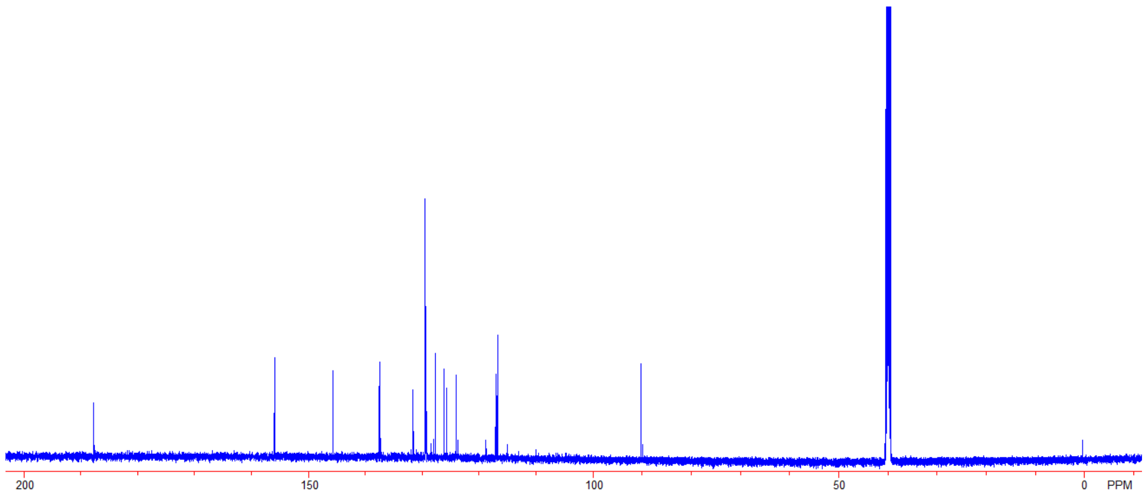

Figure S19. ^{13}C NMR (150 MHz, DMSO-d_6) spectrum of compound **16c (SYN)**.

FTIR (solid, cm^{-1}): 3055 (m, NH), 2961 (s), 2918 (s, NH), 2850 (m), 1690 (s, C=O), 1605 (m), 1578 (m), 1536 (m), 1489 (w), 1459 (m), 1400 (w), 1349 (m), 1256 (m), 1086 (s), 1012 (s), 949 (w), 862 (w), 838 (m), 789 (s), 752 (s), 660 (w).

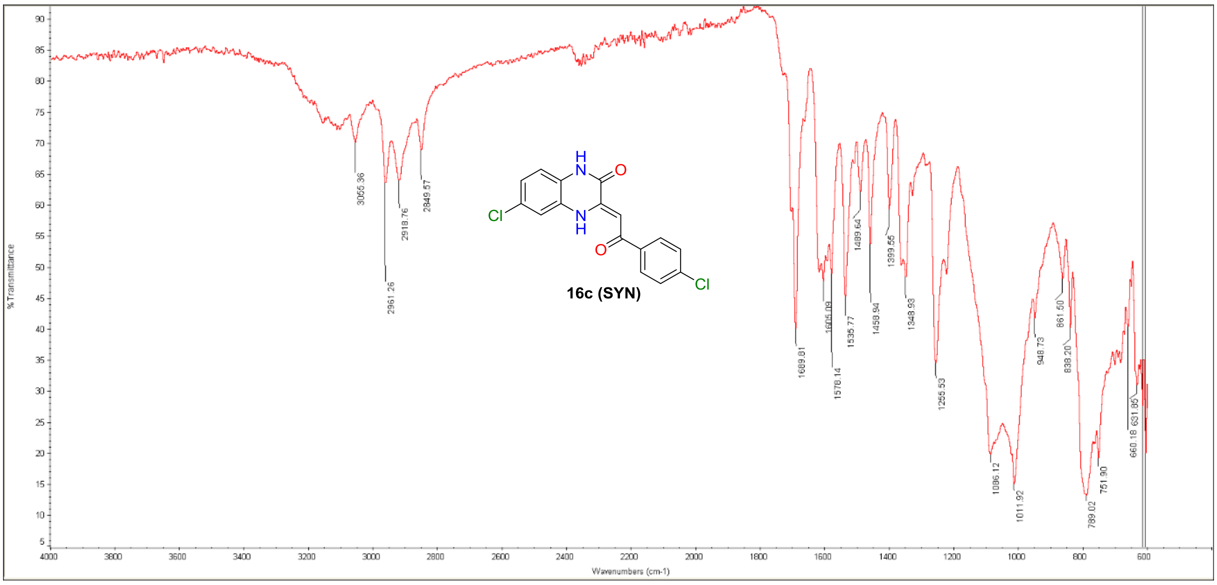

Figure S20. IR spectrum of compound **16c (SYN)**.

MS (ESI m/z): 331.0 [M-H]$^{-}$.

Anal. calcd for $C_{16}H_{10}Cl_2N_2O_2$ (333.17): C, 57.68; H, 3.03; N, 8.41. Found: C, 57.35; H, 3.10; N, 8.43.

(*Z*)-7-Chloro-3-(2-(4-chlorophenyl)-2-oxoethylidene)-3,4-dihydroquinoxalin-2(1*H*)-one (17c (ANTI))

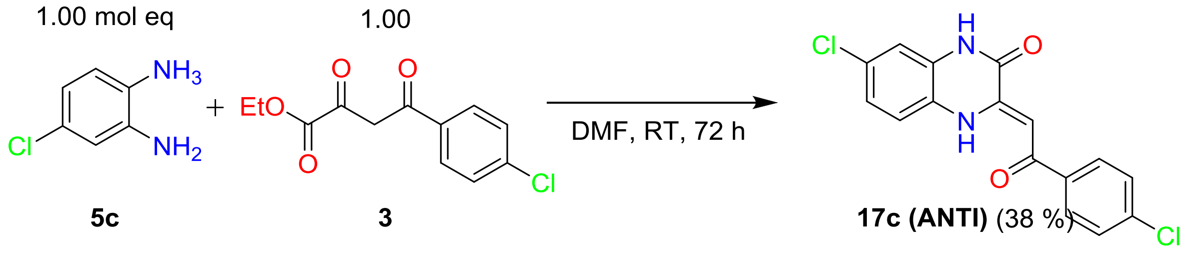

The 3,4-dihydroquinoxaline-2(1*H*)-one **17c (ANTI)** was prepared according to the general procedure A from ester **12a** and diamine **11c** without any additive. The crude mixture of ANTI / SYN regioisomers was purified by FLC (EA / H, 1 / 5) yielding 49.7 mg (0.15 mmol, 38 %) of **17c (ANTI)**.

Novelty: Compound **17c (ANTI)** was not described in the literature.

M.p.: 297.0 - 299.0 °C [EA / H], yellow solid compound.

NMR diagrams:

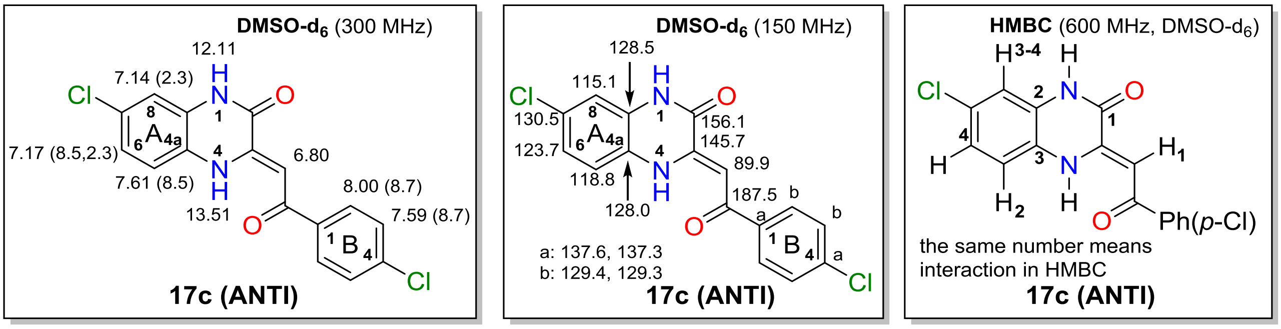

^{1}H NMR (300 MHz, DMSO-*d*$_6$): δ 13.51 (s, 1H, H-N$_A$(4)), 12.11 (s, 1H, H-N$_A$(1)), 8.00 (d, 2H, J(B$_2$,B$_3$) = 8.7 Hz, 2 x H-C$_B$(2)), 7.61 (d, 1H, J(A$_5$,A$_6$) = 8.5 Hz, H-C$_A$(5)), 7.59 (d, 2H, J(B$_2$,B$_3$) = 8.7 Hz, 2 x H-C$_B$(3)), 7.17 (dd, 1H, J(A$_5$,A$_6$) = 8.5 Hz, J(A$_6$,A$_8$) = 2.3 Hz, H-C$_A$(6)), 7.14 (d, 1H, J(A$_6$,A$_8$) = 2.3 Hz, H-C$_A$(8)), 6.80 (s, 1H, -COCH=).

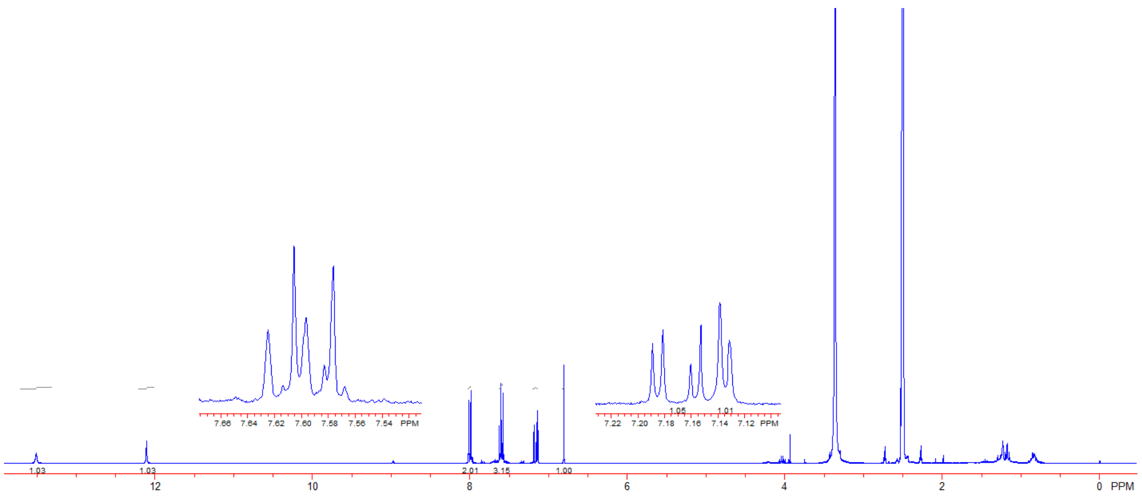

Figure S21. ^{1}H NMR (300 MHz, DMSO-d_6) spectrum of compound **17c (ANTI)**.

^{13}C NMR (150 MHz, DMSO-d_6): δ 187.5 (C_B(1)C=O), 156.1 (C_A(2)=O), 145.7 (C_A(3)), 137.6 and 137.3 (C_B(1 and 4)), 130.5 (C_A(7)), 129.4 and 129.3 (2 x C_B(2 and 3)), 128.5 (C_A(8a)), 128.0 (C_A(4a)), 123.7 (C_A(6)), 118.8 (C_A(5)), 115.1 (C_A(8)), 89.9 (-COCH=).

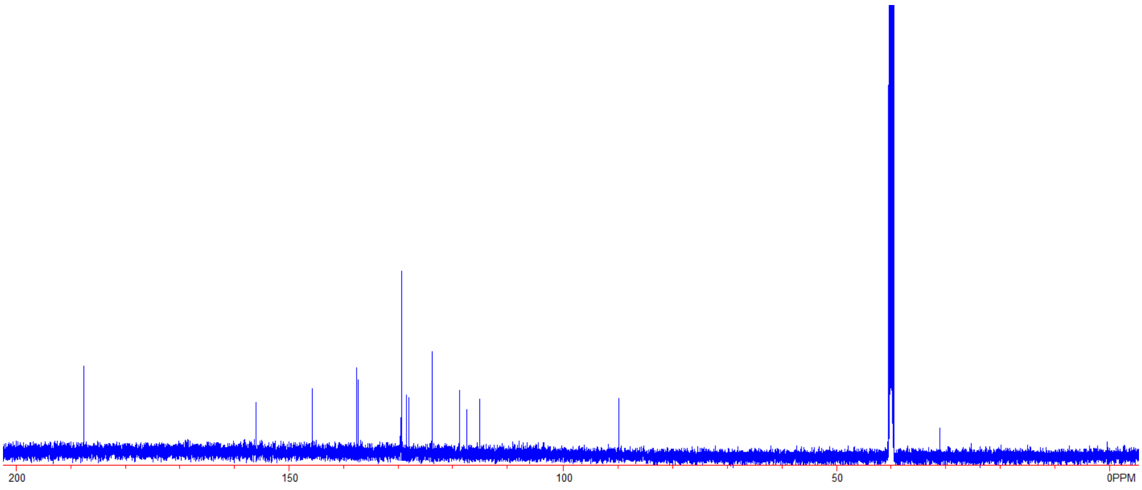

Figure S22. ^{13}C NMR (150 MHz, DMSO-d_6) spectrum of compound **17c (ANTI)**.

FTIR (solid, cm^{-1}): 3057 (m, NH), 2920 (s, NH), 2850 (m), 1680 (s, C=O), 1605 (m), 1578 (m), 1536 (m), 1489 (w), 1459 (m), 1399 (w), 1349 (m), 1251 (m), 1223 (m), 1089 (s), 1013 (s), 949 (w), 861 (w), 838 (m), 805 (s), 753 (s), 682 (w), 632 (m).

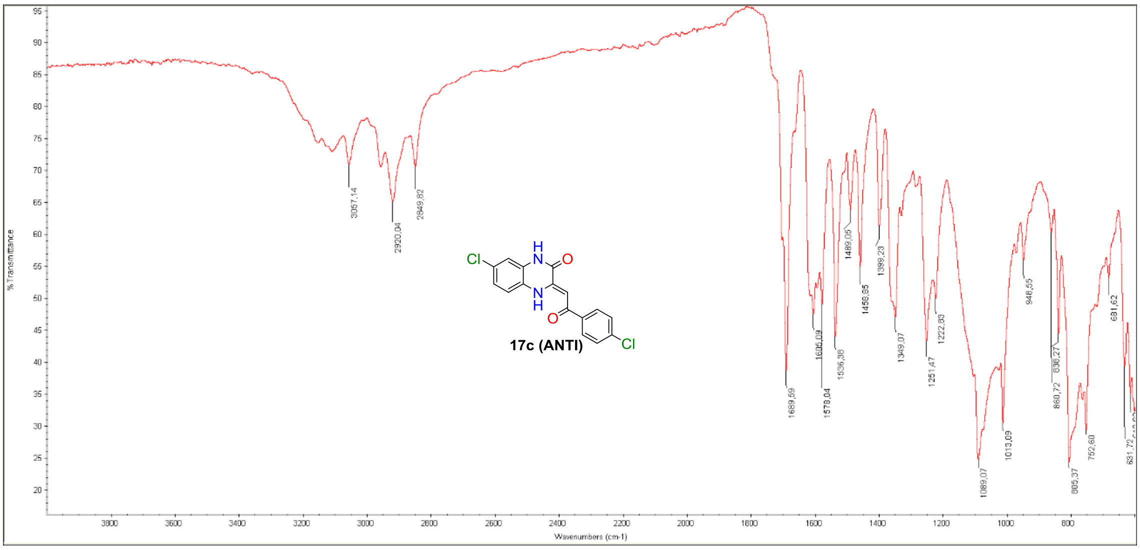

Figure S23. IR spectrum of compound **17c (ANTI)**.

MS (ESI m/z): 331.0 [M-H]$^{-}$.

Anal. calcd for $C_{16}H_{10}Cl_2N_2O_2$ (333.17): C, 57.68; H, 3.03; N, 8.41. Found: C, 57.88; H, 3.14; N, 8.35.

(*Z*)-3-(2-(4-Chlorophenyl)-2-oxoethylidene)-2-oxo-1,2,3,4-tetrahydroquinoxaline-6-carboxylic acid (16d (SYN))

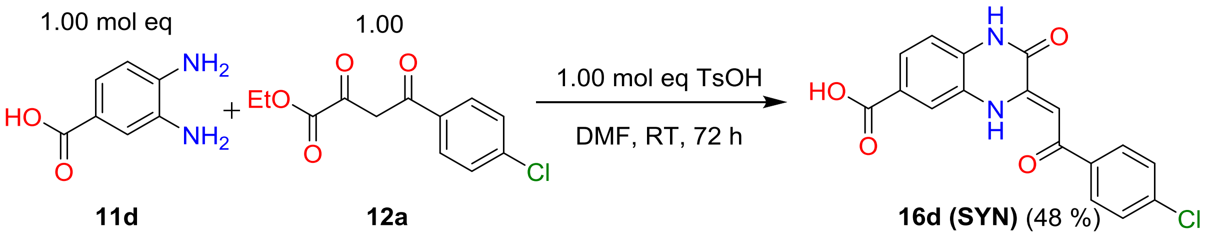

The 3,4-dihydroquinoxaline-2(1*H*)-one **16d (SYN)** was prepared according to the general procedure A from ester **12a** diamine **11d** and *p*-TsOH as additive. The crude mixture of ANTI

/ SYN regioisomers was purified by crystallization from DMSO yielding 64.6 mg (0.19 mmol, 48 %) **16d (SYN)**.

Novelty: Compound **16d (SYN)** was not described in the literature.

M.p.: 363.0 - 365.0 °C [DMSO], yellow solid compound.

NMR diagrams:

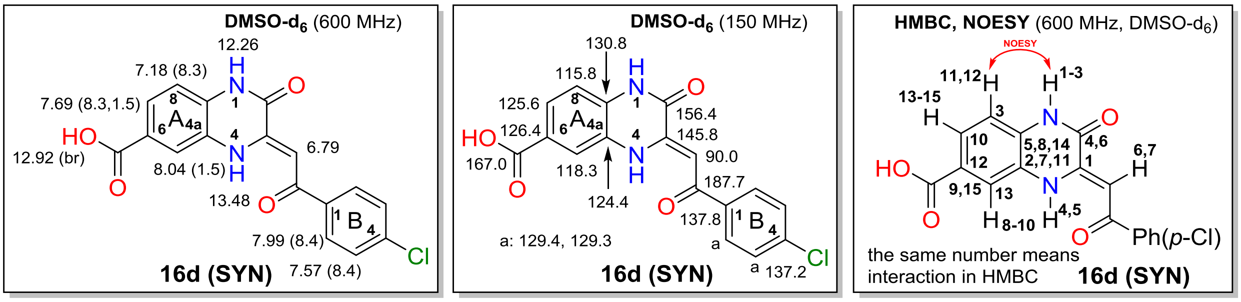

^{1}H NMR (600 MHz, DMSO-d_6): δ 13.48 (s, 1H, H-N_A(4)), 12.92 (br s, 1H, -COOH), 12.26 (s, 1H, H-N_A(1)), 8.04 (d, 1H, $J(A_5,A_7)$ = 1.5 Hz, H-C_A(5)), 7.99 (d, 2H, $J(B_2,B_3)$ = 8.4 Hz, 2 x H-C_B(2)), 7.69 (dd, 1H, $J(A_7,A_8)$ = 8.3 Hz, $J(A_5,A_7)$ = 1.5 Hz, H-C_A(7)), 7.57 (d, 2H, $J(B_2,B_3)$ = 8.4 Hz, 2 x H-C_B(3)), 7.18 (d, 1H, $J(A_7,A_8)$ = 8.3 Hz, H-C_A(8)), 6.79 (s, 1H, -COCH=).

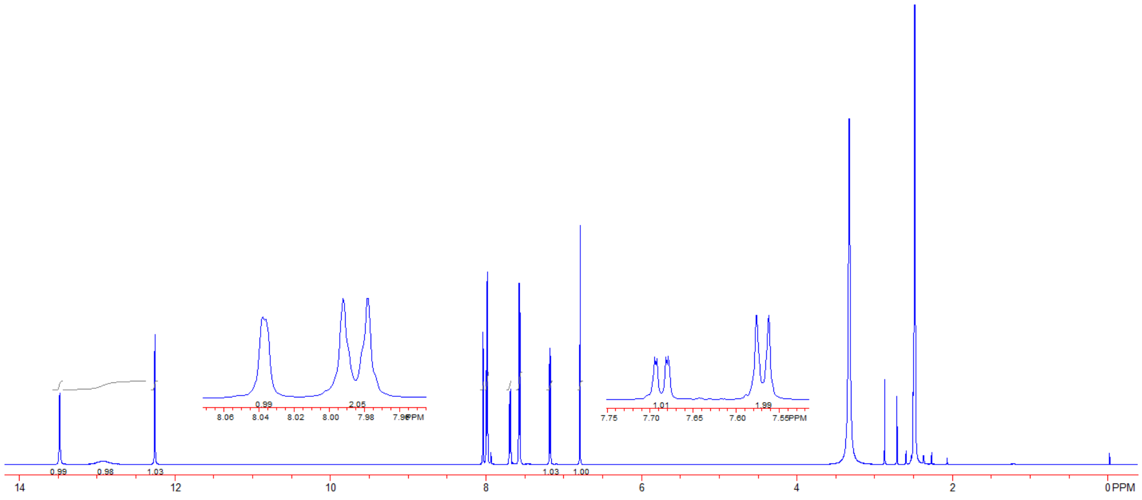

Figure S24. ^{1}H NMR (600 MHz, DMSO-d_6) spectrum of compound **16d (SYN)**.

^{13}C NMR (150 MHz, DMSO-d_6): δ 187.7 (C_B(1)$\underline{C}$=O), 167.0 (-COOH), 156.4 (C_A(2)), 145.8 (C_A(3)), 137.8 (C_B(1)), 137.2 (C_B(4)), 130.8 (C_A(8a)), 129.4 and 129.3 (2 x C_B(2 and 3)), 126.4 (C_A(6)), 125.6 (C_A(7)), 124.4 (C_A(4a)), 118.3 (C_A(5)), 115.8 (C_A(8)), 90.0 (-CO$\underline{C}$H=).

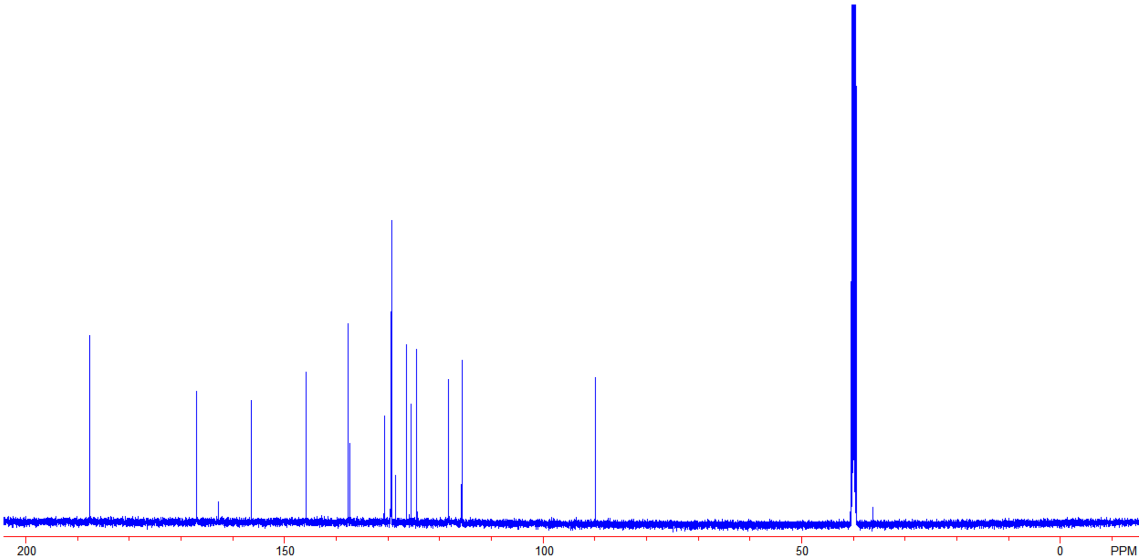

Figure S25. ^{13}C NMR (150 MHz, DMSO-d_6) spectrum of compound **16d (SYN)**.

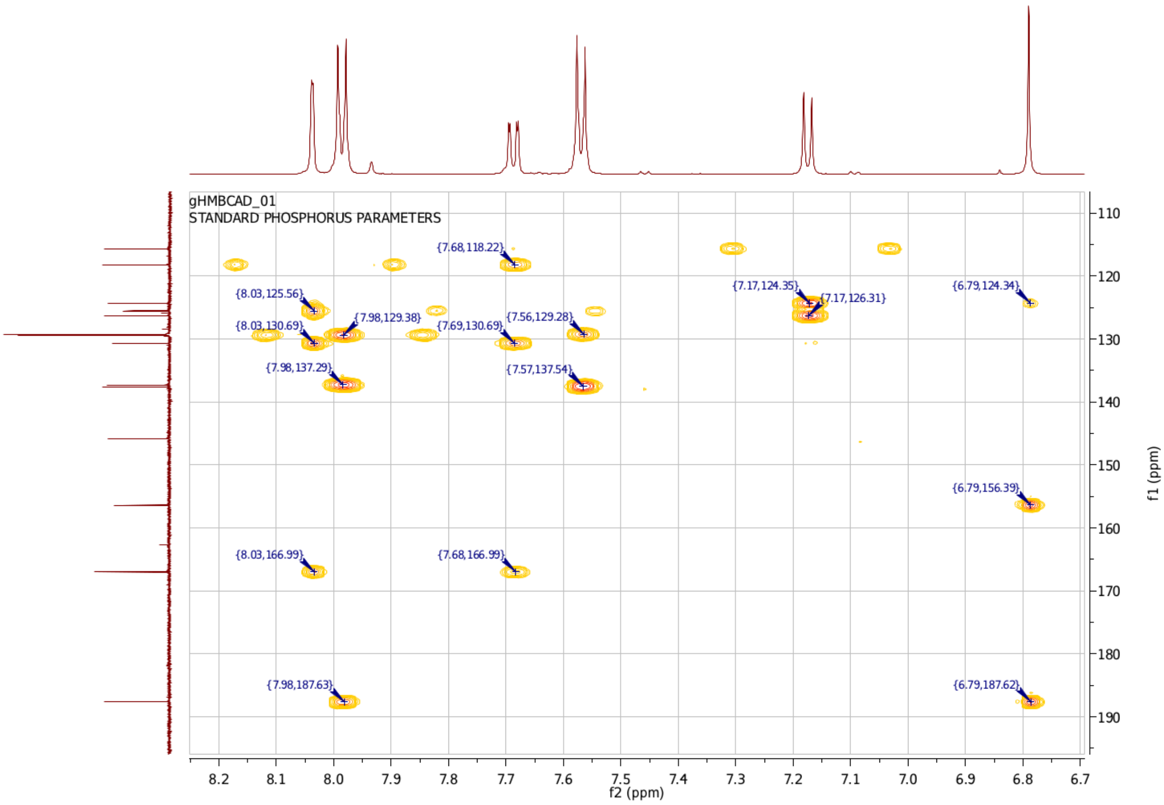

Figure S26. Part of HMBC NMR spectra of compound **16d (SYN)** with peak (6.79, 124.34) that confirms regioisomerism.

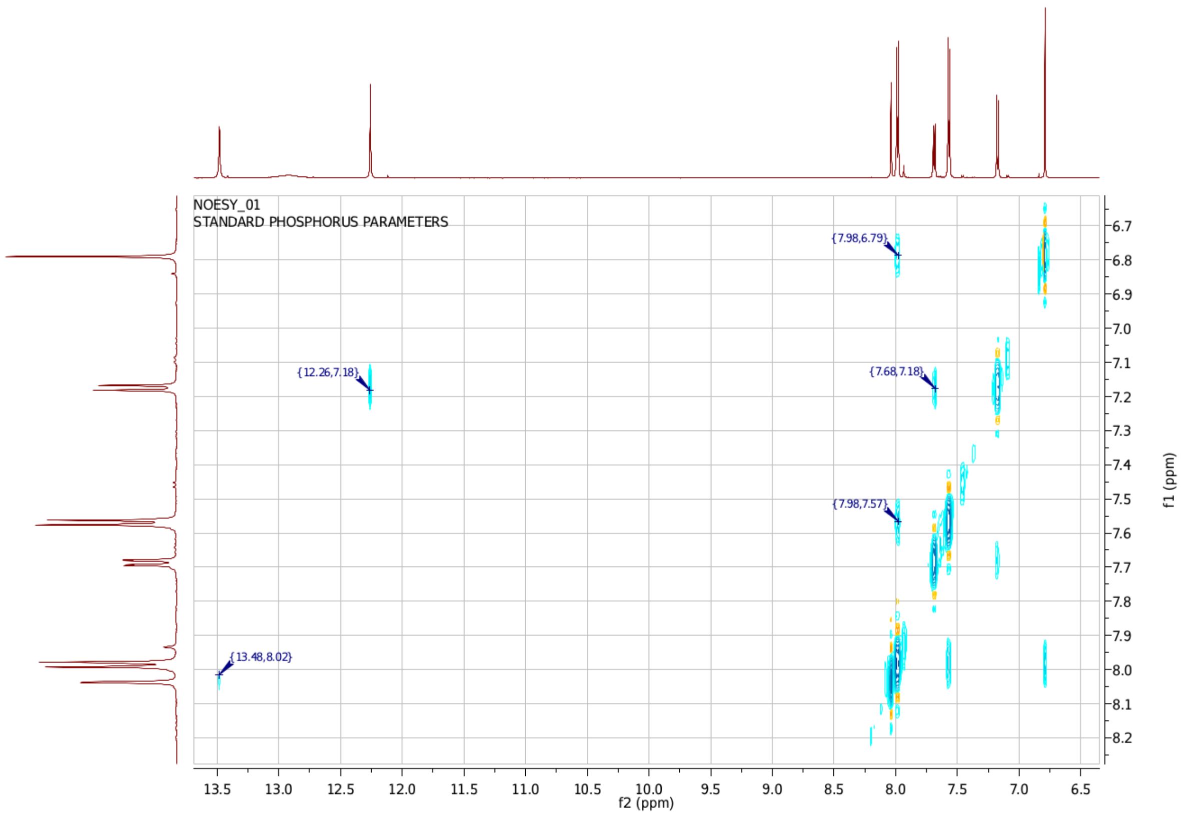

Figure S27. Part of NOESY NMR spectra of compound **16d (SYN)** with peaks (12.26, 7.18; 13.48, 8.02) that confirm regioisomerism.

FTIR (solid, cm^{-1}): 3184 (s, -OH), 2925 (m), 1732 (w), 1688 (s, C=O), 1615 (s), 1586 (s), 1550 (w), 1486 (w), 1366 (m), 1247 (m), 1218 (m), 1095 (m), 1065 (w), 1011 (w), 787 (w), 750 (m).

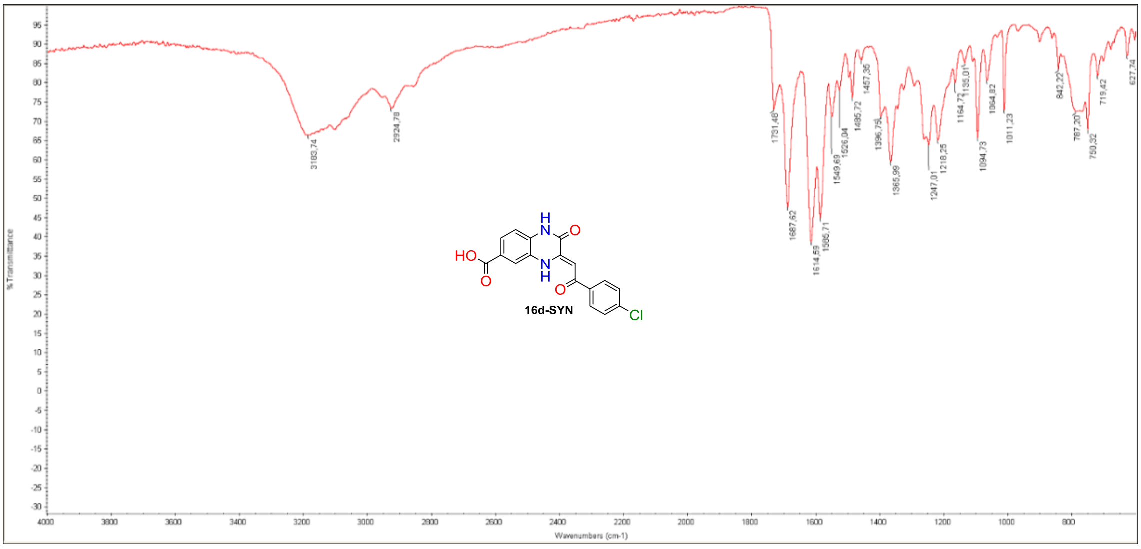

Figure S28. IR spectrum of compound **16d**.

MS (ESI m/z): 341.2 [M-H]$^-$.

Anal. calcd for $C_{17}H_{11}ClN_2O_4$ (342.73): C, 59.57; H, 3.23; Cl, 10.34; N, 8.17. Found: C, 59.40; H, 3.27; Cl, 10.38; N, 8.04.

(*Z*)-2-(2-(4-Chlorophenyl)-2-oxoethylidene)-3-oxo-1,2,3,4-tetrahydroquinoxaline-6-carboxylic acid (17d (ANTI))

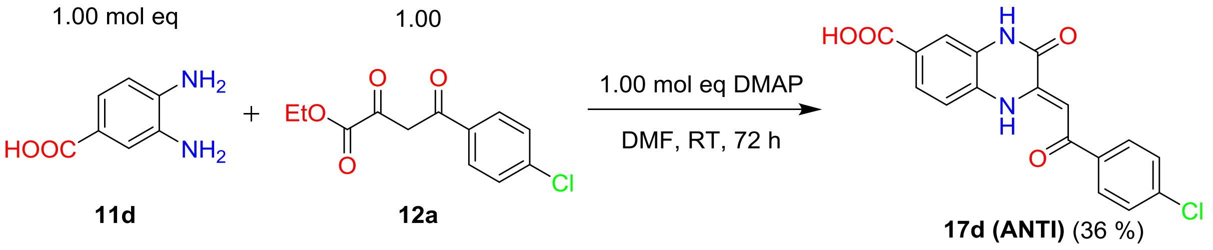

The 3,4-dihydroquinoxaline-2(1*H*)-one **17d (ANTI)** was prepared according to the general procedure A from diamine **11d**, ester **12a** and (1.00 equiv) of DMAP as additive. The crude product was crystallized from DMSO and obtained as salt with DMAP. To liberate free acid

17d (ANTI), the salt was suspended in 1 M HCl, stirred for 24 h, solid material filtered off, washed with water and dried yielding 48.4 mg (0.14 mmol, 36%) of **17d (ANTI)**.

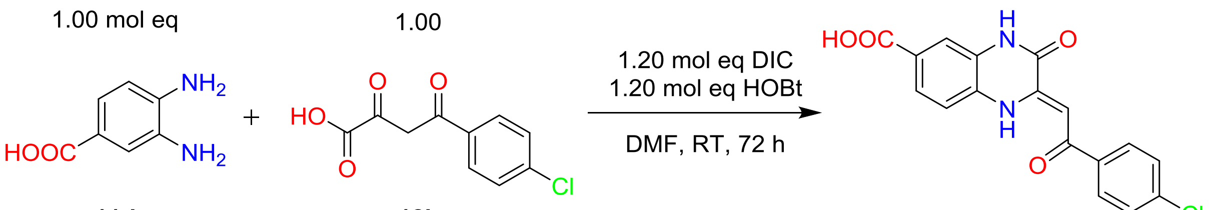

11d **12b** **17d (ANTI)** (70 %)

Alternatively **17d (ANTI)** was prepared also by the general **procedure B** from diamine **11d** and acid **12b**. The crude product crystallized from DMSO to yield 105.9 mg (0.31 mmol, 70 %) of **17d (ANTI)**.

Novelty: Compound **17d (ANTI)** was not described in the literature.

M.p.: 391.0 - 392.0 °C [DMSO], yellow solid compound.

NMR diagrams:

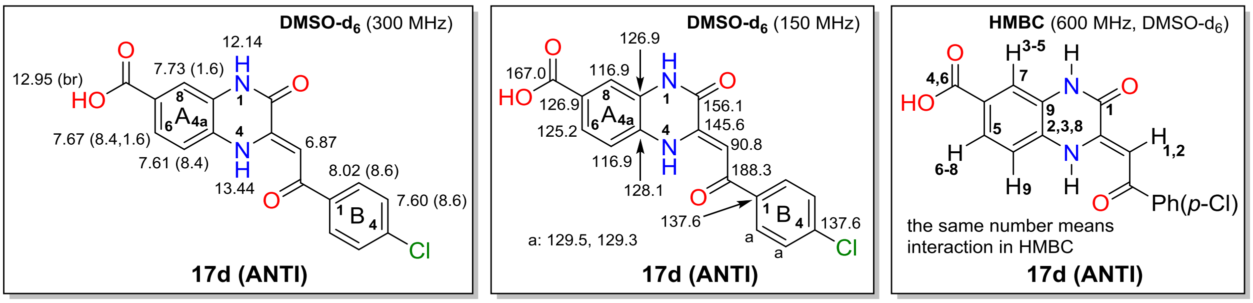

17d (ANTI) **17d (ANTI)** **17d (ANTI)**

^{1}H NMR (300 MHz, DMSO-d_6): δ 13.44 (s, 1H, H-N_A(4)), 12.95 (br s, 1H, -COOH), 12.14 (s, 1H, H-N_A(1)), 8.02 (d, 2H, J(B_2,B_3) = 8.6 Hz, 2 x H-C_B(2)), 7.73 (d, 1H, J(A_6,A_8) = 1.6 Hz, H-C_A(8)), 7.67 (dd, 1H, J(A_5,A_6) = 8.4 Hz, J(A_6,A_8) = 1.6 Hz, H-C_A(6)), 7.61 (d, 1H, J(A_5,A_6) = 8.4 Hz, H-C_A(5)), 7.60 (d, 2H, J(B_2,B_3) = 8.6 Hz, 2 x H-C_B(3)), 6.87 (s, 1H, -COCH=).

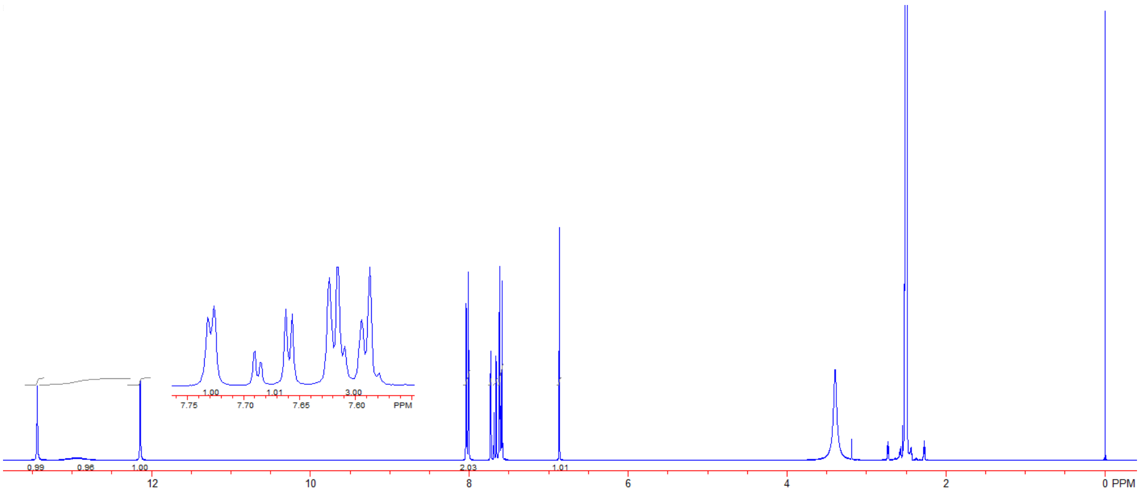

Figure S29. ^{1}H NMR (300 MHz, DMSO-d_6) spectrum of compound **17d (ANTI)**.

^{13}**C NMR** (150 MHz, DMSO-d_6): δ 188.3 ($C_B(1)\underline{C}$=O), 167.0 (-COOH), 156.1 (C_A(2)=O), 145.6 (C_A(3)), 2 x 137.6 (C_B(1 and 4)), 129.5 and 129.3 (2 x C_B(2 and 3)), 128.1 (C_A(4a)), 2 x 126.9 (C_A(8a and 7)), 125.2 (C_A(6)), 2 x 116.9 (C_A(5 and 8)), 90.8 (-CO$\underline{C}$H=).

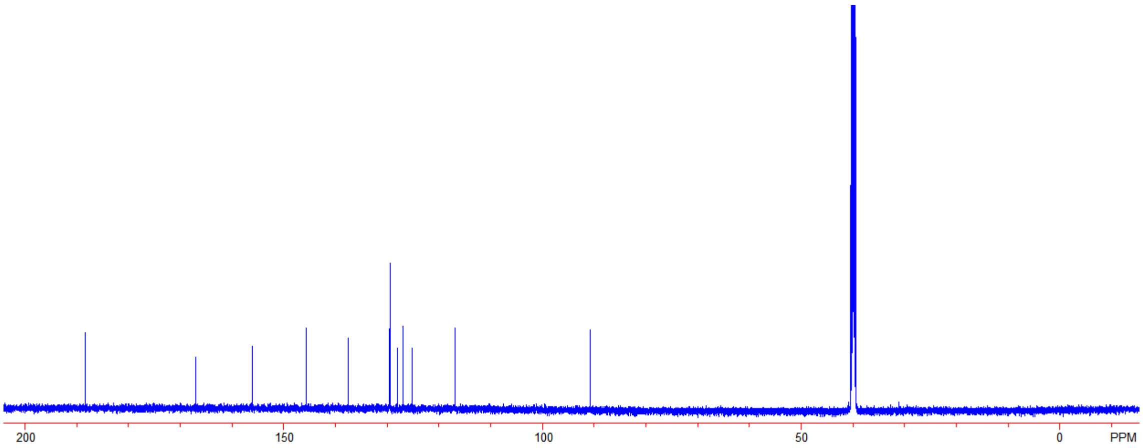

Figure S30. ^{13}C NMR (150 MHz, DMSO-d_6) spectrum of compound **17d (ANTI)**.

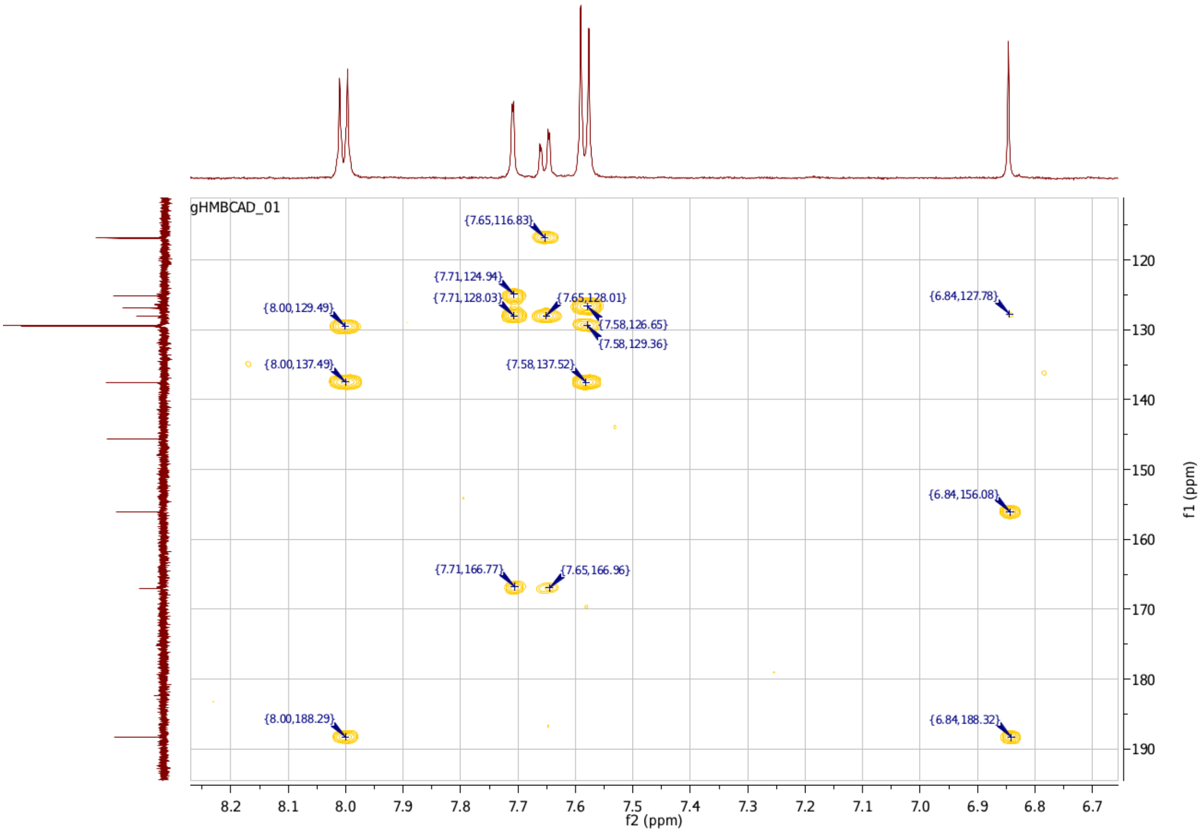

Figure S31. Part of HMBC NMR spectra of **17d (ANTI)** with peak (6.84, 127.78) that confirms regioisomerism.

FTIR (solid, cm^{-1}): 3486 (m), 3206 (s, -OH), 2634 (w), 1706 (s, C=O), 1661 (w), 1628 (m), 1586 (s, C=O), 1522 (w), 1398 (m), 1374 (w), 1291 (m), 1248 (m), 1184 (m), 1093 (w), 1056 (m), 1009 (w), 899 (w), 781 (w), 764 (w), 721 (w).

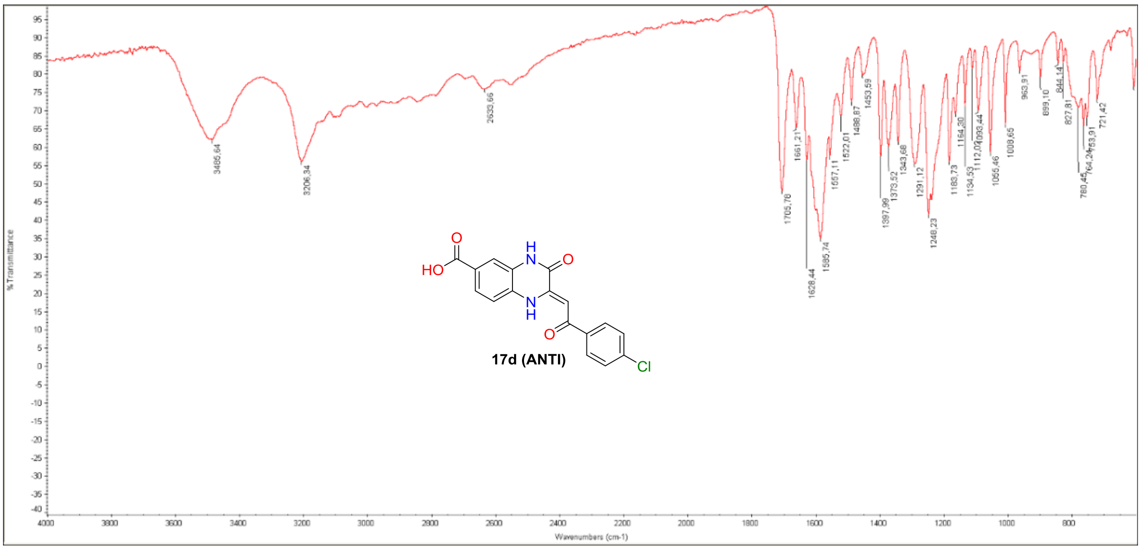

Figure S32. IR spectrum of compound **17d (ANTI)**.

MS (ESI m/z): 341.0 [M-H]$^-$.

Anal. calcd for $C_{17}H_{11}ClN_2O_4$ (342.73): C, 59.57; H, 3.23; N, 8.17. Found: C, 59.50; H, 3.20; N, 8.20.

(*Z*)-3-(2-(4-Chlorophenyl)-2-oxoethylidene)-2-oxo-1,2,3,4-tetrahydroquinoxaline-6-carbonitrile (16e (SYN))

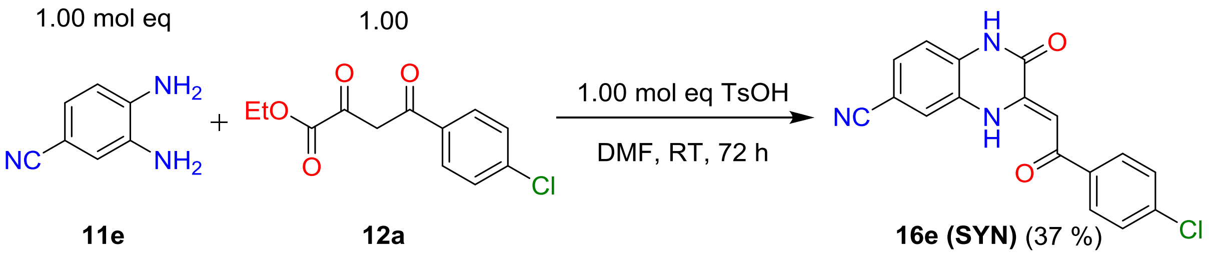

The 3,4-dihydroquinoxaline-2(1*H*)-one **16e (SYN)** was prepared according to the general procedure A from ester **12a** diamine **11e** and ***p*-TsOH** as additive. The crude mixture of ANTI

/ SYN regioisomers was purified by precipitation from DMSO by H_2O yielding 52.8 mg (0.16 mmol, 37%) **16e (SYN)**.

Novelty: Compound **16e (SYN)** was described in the literature by M.p.[5]

M.p.: 317.6 – 319.4 °C [DMSO], yellow solid compound (lit. 295 - 296 °C [EtOH]).[5]

NMR diagrams:

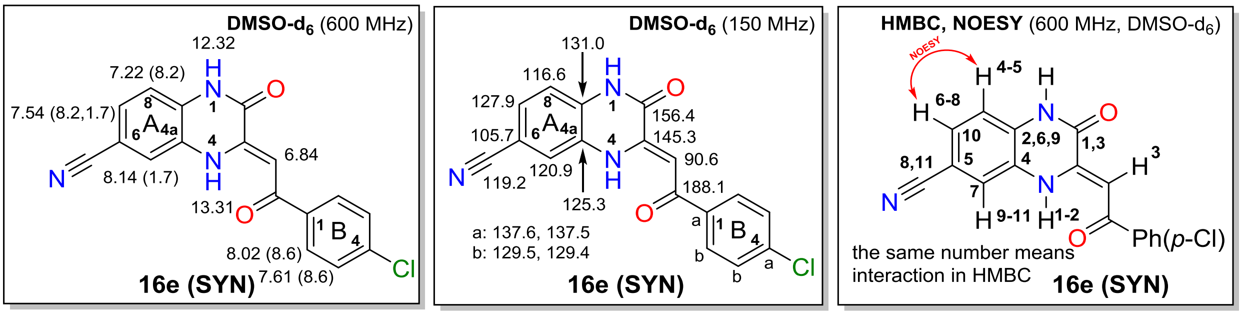

^{1}H NMR (600 MHz, DMSO-d_6): δ 13.31 (s, 1H, H-N_A(4)), 12.32 (s, 1H, H-N_A(1)), 8.14 (d, 1H, $J(A_5,A_7)$ = 1.7 Hz, H-C_A(5)), 8.02 (d, 2H, $J(B_2,B_3)$ = 8.6 Hz, 2 x H-C_B(2)), 7.61 (d, 2H, $J(B_2,B_3)$ = 8.6 Hz, 2 x H-C_B(3)), 7.54 (dd, 1H, $J(A_7,A_8)$ = 8.2 Hz, $J(A_5,A_7)$ = 1.7 Hz, H-C_A(7)), 7.22 (d, 1H, $J(A_7,A_8)$ = 8.2 Hz, H-C_A(8)), 6.84 (s, 1H, -COCH=).

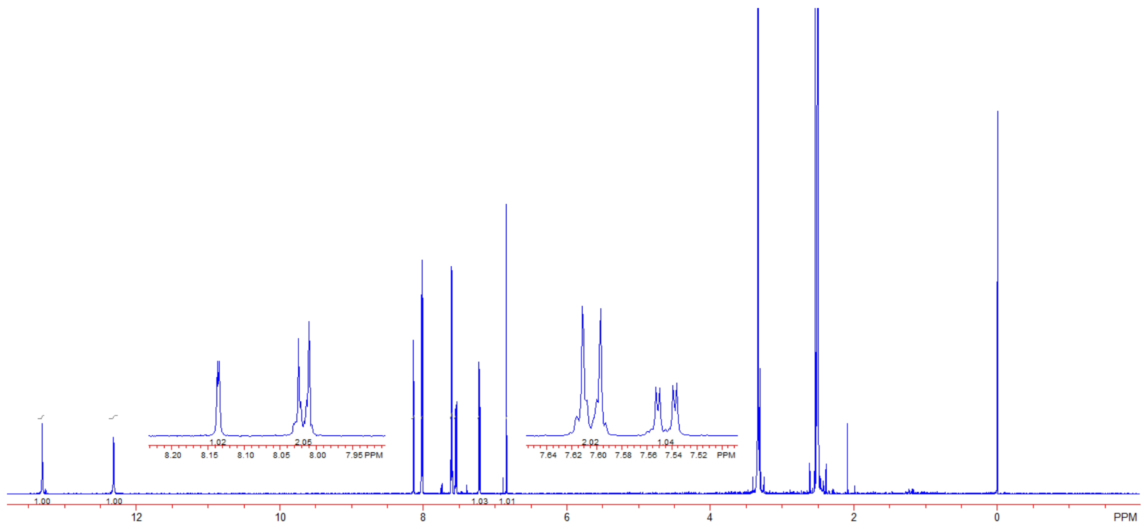

Figure S33. ^{1}H NMR (600 MHz, DMSO-d_6) spectrum of compound **16e (SYN)**.

^{13}C NMR (150 MHz, DMSO-d_6): δ 188.1 (C_B(1)$\underline{C}$=O), 156.4 (C_A(2)), 145.3 (C_A(3)), 137.6 and 137.5 (C_B(1 and 4)), 131.0 (C_A(8a)), 129.5 and 129.4 (2 x C_B(2 and 3)), 127.9 (C_A(7)), 125.3 (C_A(4a)), 120.9 (C_A(5)), 119.2 (CN), 116.6 (C_A(8)), 105.7 (C_A(6)), 90.6 (-CO$\underline{C}$H=).

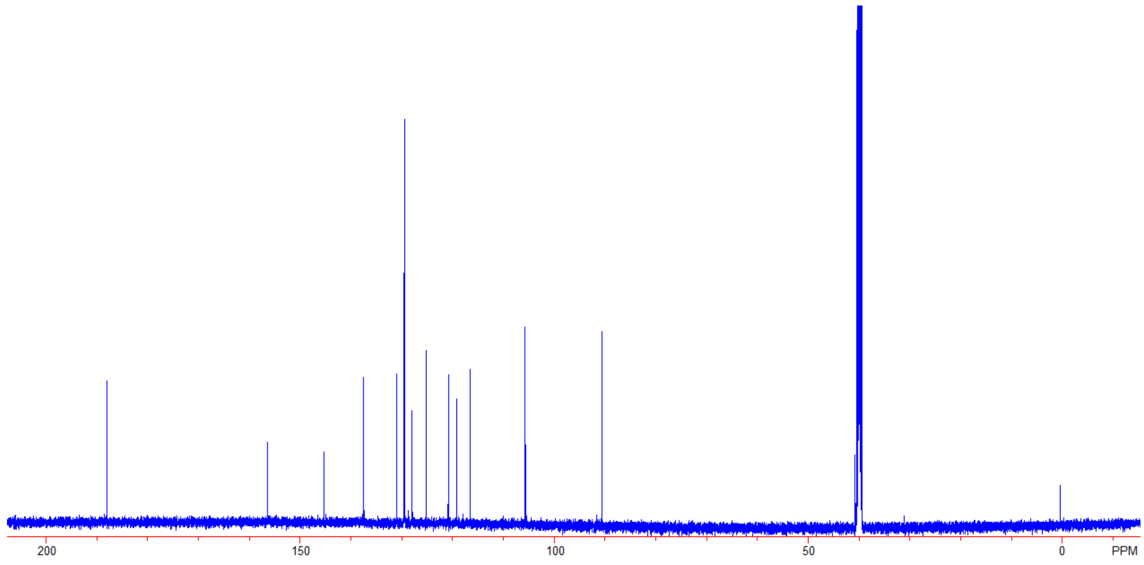

Figure S34. ^{13}C NMR (150 MHz, DMSO-d_6) spectrum of compound **16e (SYN)**.

FT IR (solid, cm^{-1}): 3072 (s), 2231 (s, C≡N), 1688 (s, C=O), 1610 (s, C=O), 1575 (s), 1533 (s), 1495 (m), 1456 (w), 1397 (w), 1352 (s), 1260 (m), 1235 (m), 1170 (m), 1139 (w), 1088 (m), 1057 (m), 1014 (m), 973 (m), 906 (m), 782 (s), 753 (s), 680 (m), 655 (m), 615 (s).

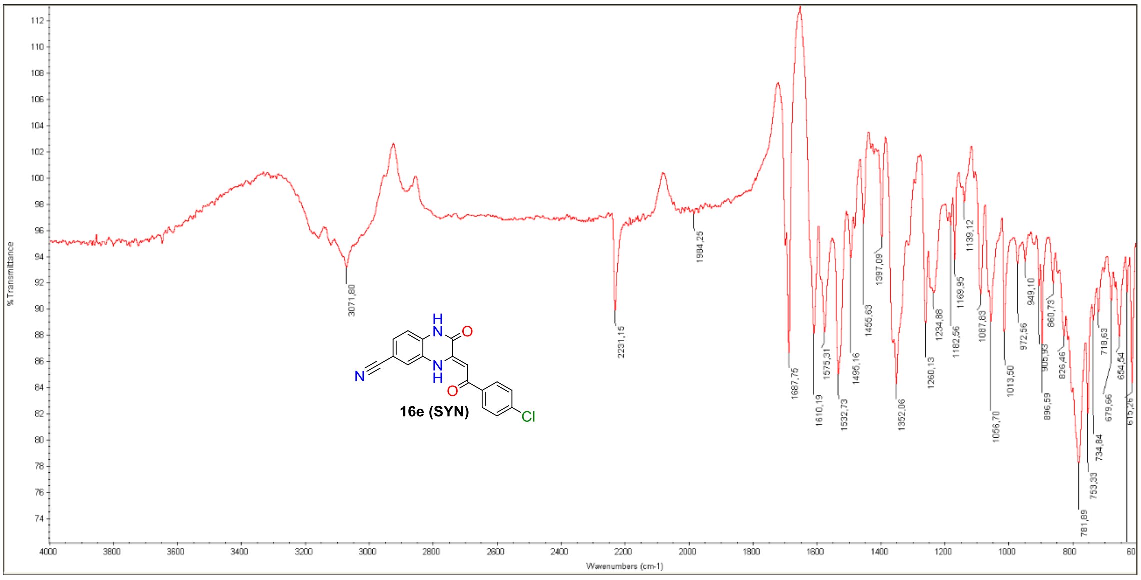

Figure S35. IR spectrum of compound **16e (SYN)**.

MS (ESI m/z): 322.0 [M-H]$^{-}$.

Anal. calcd for $C_{17}H_{10}ClN_3O_2$ (323.73): C, 63.07; H, 3.11; N, 12.98. Found: C, 63.31; H, 3.19; N, 13.12.

(*Z*)-2-(2-(4-Chlorophenyl)-2-oxoethylidene)-3-oxo-1,2,3,4-tetrahydroquinoxaline-6-carbonitrile (17e (ANTI))

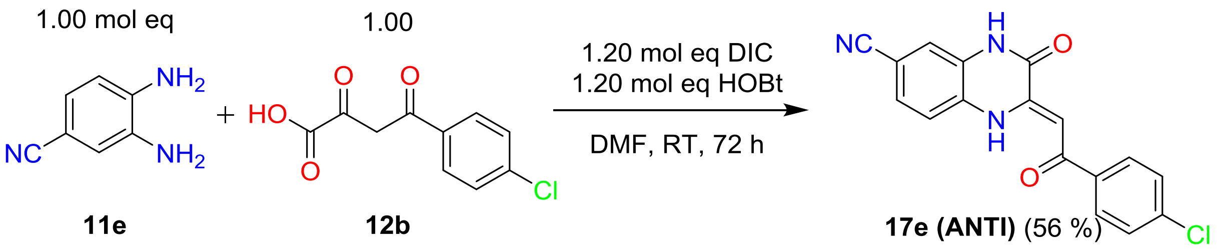

The 3,4-dihydroquinoxaline-2(1*H*)-one **17e (ANTI)** was prepared according to the general procedure B from acid **12b** diamine **11e**. The crude mixture of ANTI / SYN regioisomers was purified by FLC (EA / H, 1 / 2) yielding 80.0 mg (0.25 mmol, 56%) of **17e (ANTI)**.

Novelty: Compound **17e (ANTI)** was described in the literature by its M.p.[5]

M.p.: 354.0 – 355.0 °C [MeOH], yellow solid compound (lit. 311 - 312 °C [EtOH]).[5]

NMR diagrams:

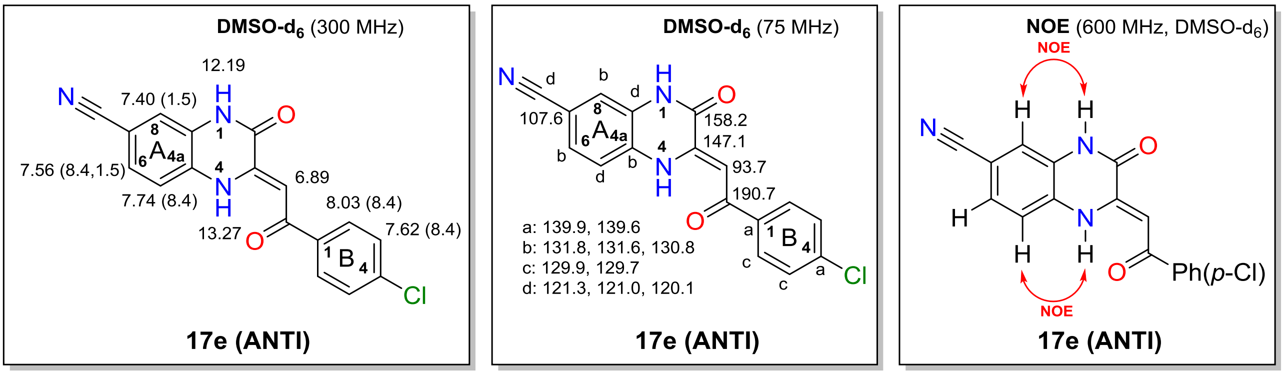

---

[5] Andreichikov, Yu. S.; Nekrasov, D. D.; Pitirimova, S. G.; Zaks, A. S.; Korsheninnikova, M. I.; Plaksina, P. N.; Semenova, Z. N.; Kopeikin, V. A. *Khim Farm Zh* **1989**, *23*, 946-949.

^{1}H NMR (300 MHz, DMSO-*d*$_6$): *δ* 13.27 (s, 1H, H-N_A(4)), 12.19 (s, 1H, H-N_A(1)), 8.03 (d, 2H, *J*(B_2,B_3) = 8.4 Hz, 2 x H-C_B(2)), 7.74 (d, 1H, *J*(A_5,A_6) = 8.4 Hz, H-C_A(5)), 7.62 (d, 2H, *J*(B_2,B_3) = 8.4 Hz, 2 x H-C_B(3)), 7.56 (dd, 1H, *J*(A_5,A_6) = 8.4 Hz, *J*(A_6,A_8) = 1.5 Hz, H-C_A(6)), 7.40 (d, 1H, *J*(A_6,A_8) = 1.5 Hz, H-C_A(8)), 6.89 (s, 1H, -COCH=).

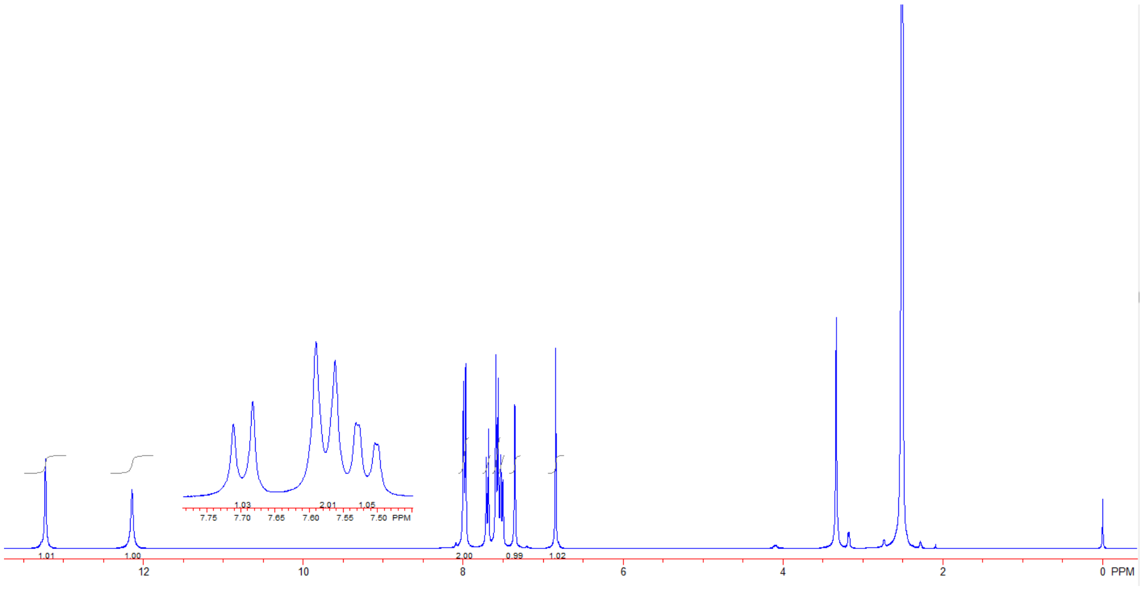

Figure S36. ^{1}H NMR (300 MHz, DMSO-*d*$_6$) spectrum of compound **17e (ANTI)**.

^{13}C NMR (75 MHz, DMSO-*d*$_6$): δ 190.7 (C_B(1)C=O), 158.2 (C_A(2)), 147.1 (C_A(3)), 139.9 and 139.6 (C_B(1 and 4)), 131.8, 131.6 and 130.8 (C_A(4a, 6 and 8)), 129.9 and 129.7 (2 x C_B(2 and 3)), 121.3, 121.0 and 120.1 (C_A(5 and 8a) and -CN), 107.6 (C_A(7)), 93.7 (-COCH=).

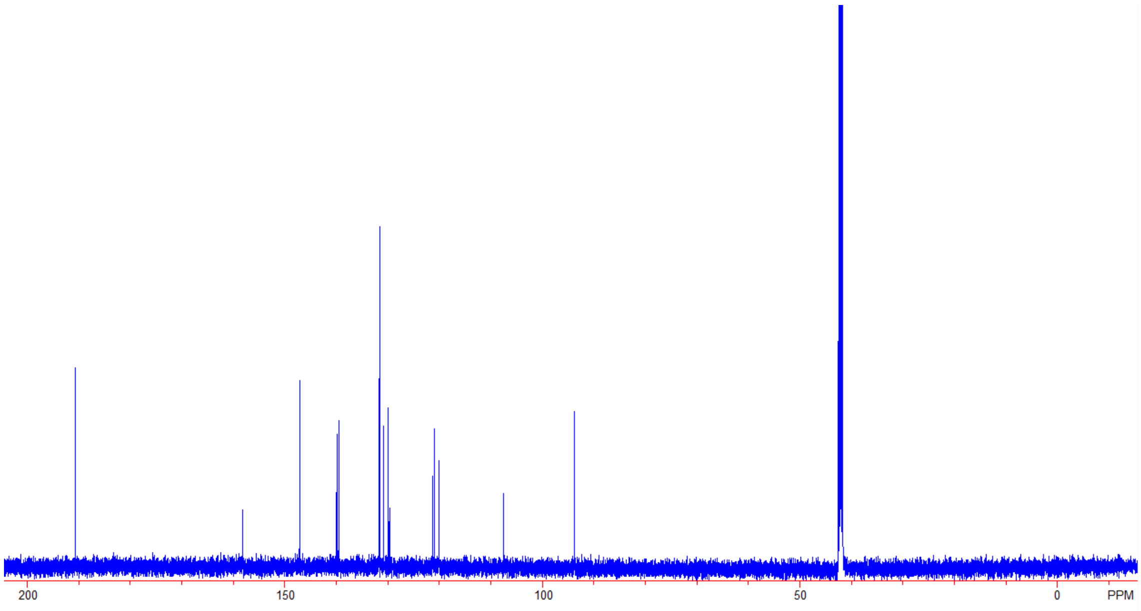

Figure S37. ^{13}C NMR (75 MHz, DMSO-d_6) spectrum of compound **17e (ANTI)**.

FTIR (solid, cm^{-1}): 3094 (w, NH), 2912 (m, NH), 2225 (s, CN), 1682 (m, C=O), 1577 (s, C=O), 1549 (s), 1526 (m), 1456 (m), 1399 (m), 1354 (s), 1344 (s), 1269 (m), 1247 (s), 1166 (m), 1085 (m), 1058 (s), 1012 (s), 876 (m), 842 (s), 819 (s), 799 (s), 757 (s), 661 (s), 611 (s).

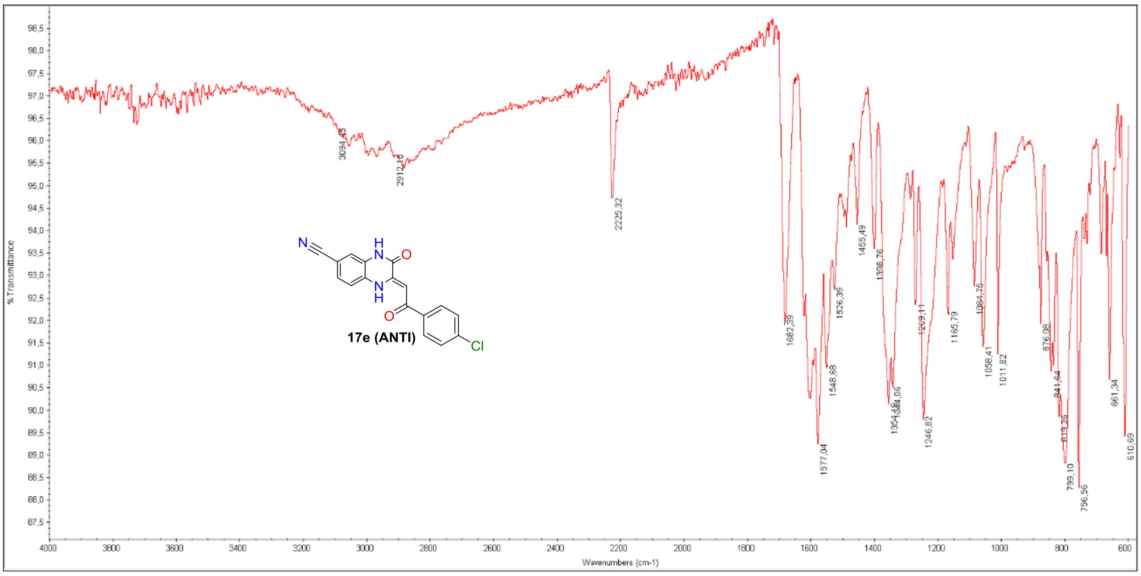

Figure S38. IR spectrum of compound **17e (ANTI)**.

MS (ESI m/z): 322.1 [M-H]⁻.

Anal. calcd for $C_{17}H_{10}ClN_3O_2$ (323.73): C, 63.07; H, 3.11; N, 12.98. Found: C, 63.11; H, 3.18; N, 12.90.

(*Z*)-3-(2-(4-Chlorophenyl)-2-oxoethylidene)-6-nitro-3,4-dihydroquinoxalin-2(1*H*)-one (16f (SYN))

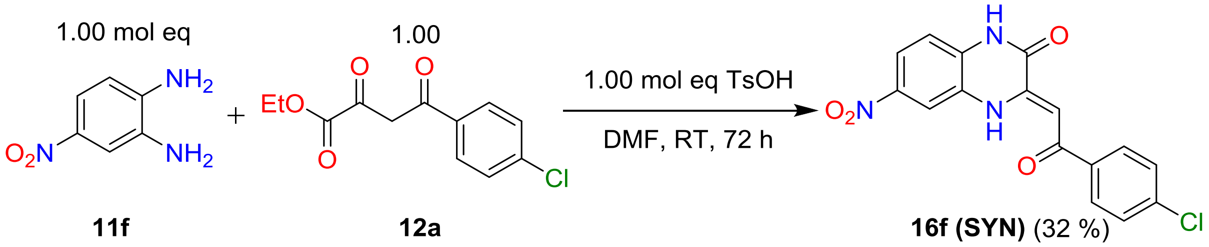

The 3,4-dihydroquinoxaline-2(1*H*)-one **16f (SYN)** was prepared according to the general procedure A from ester **12a** diamine **11f** and *p*-TsOH as additive. The crude mixture of ANTI / SYN regioisomers was purified by trituration with EA and crystallization from DMSO yielding 48.5 mg (0.14 mmol, 32 %) **16f (SYN)**.

Novelty: Compound **16f (SYN)** was not described in the literature.

M.p.: 325.0 - 328.0 °C [DMSO], yellow solid compound.

NMR diagrams:

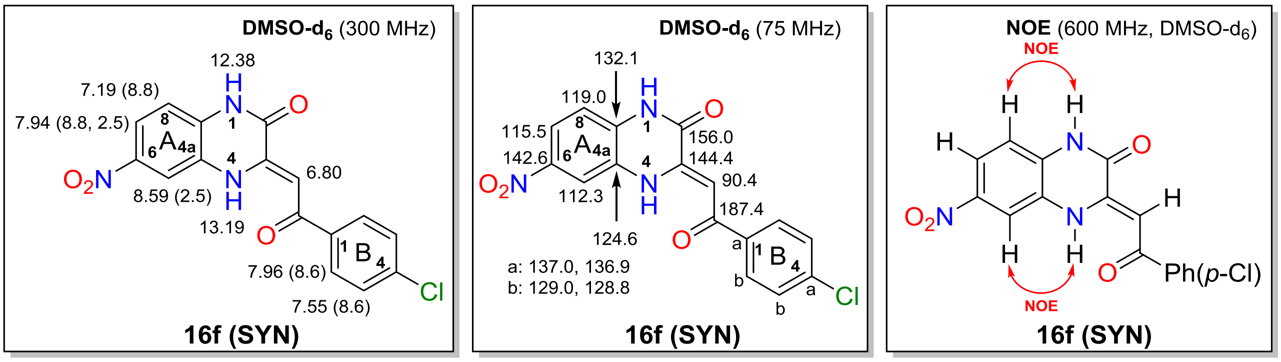

^{1}H NMR (300 MHz, DMSO-d_6): δ 13.19 (s, 1H, H-N$_A$(4)), 12.38 (s, 1H, H-N$_A$(1)), 8.59 (d, 1H, J(A$_5$,A$_7$) = 2.5 Hz, H-C$_A$(5)), 7.96 (d, 2H, J(B$_2$,B$_3$) = 8.6 Hz, 2 x H-C$_B$(2)), 7.94 (dd, 1H, J(A$_7$,A$_8$) = 8.8, J(A$_5$,A$_7$) = 2.5 Hz, H-C$_A$(7)), 7.55 (d, 2H, J(B$_2$,B$_3$) = 8.6 Hz, 2 x H-C$_B$(3)), 7.19 (d, 1H, J(A$_7$,A$_8$) = 8.8 Hz, H-C$_A$(8)), 6.80 (s, 1H, -COCH=).

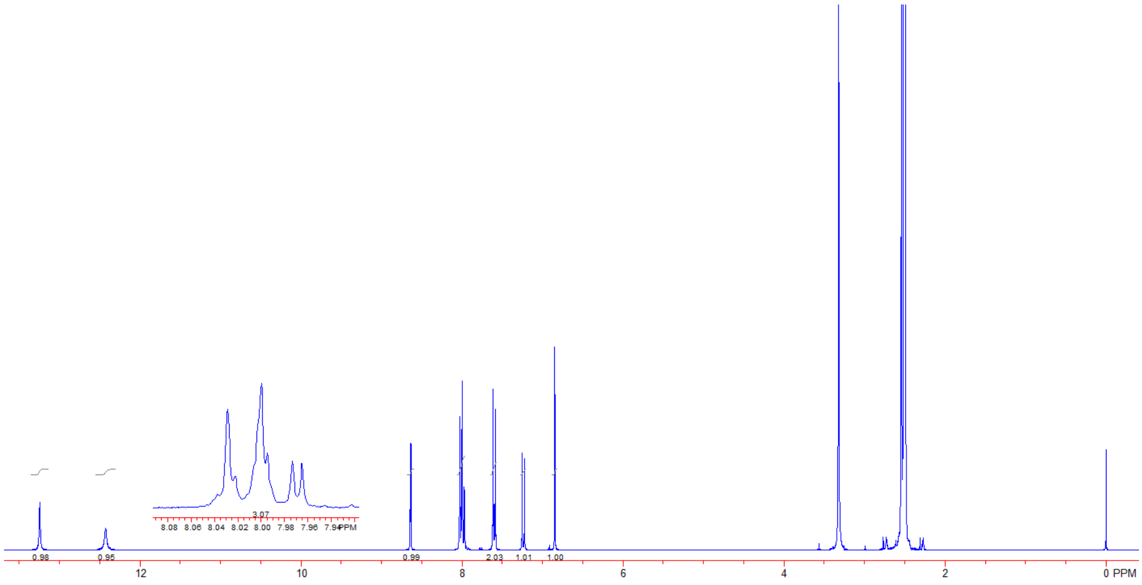

Figure S39. ^{1}H NMR (300 MHz, DMSO-d_6) spectrum of compound **16f (SYN)**.

^{13}C NMR (75 MHz, DMSO-d_6): δ 187.4 (C$_B$(1)<u>C</u>=O), 156.0 (C$_A$(2)), 144.4 (C$_A$(3)), 142.6 (C$_A$(6)), 137.0 and 136.9 (C$_B$(1 and 4)), 132.1 (C$_A$(8a)), 129.0 and 128.8 (2 x C$_B$(2 and 3)), 124.6 (C$_A$(4a)), 119.0 (C$_A$(8)), 115.5 (C$_A$(7)), 112.3 (C$_A$(5)), 90.4 (-CO<u>C</u>H=).

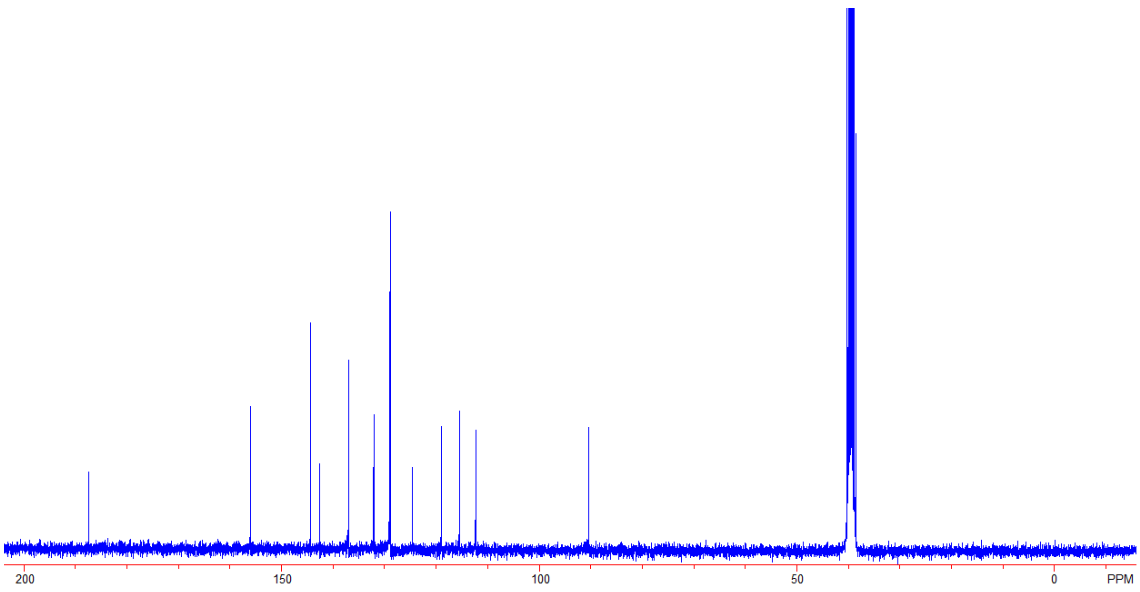

Figure S40. ^{13}C NMR (75 MHz, DMSO-d_6) spectrum of compound **16f (SYN)**.

FTIR (solid, cm^{-1}): 3090 (m, NH), 2855 (m, NH), 1682 (m, C=O), 1601 (s, C=O), 1579 (s), 1541 (m), 1482 (m, NO_2), 1433 (w), 1411 (w), 1352 (m), 1316 (s), 1279 (s), 1265 (m), 1245 (m), 1172 (m), 1133 (m), 1087 (m), 1032 (s), 1008 (s), 953 (m), 937 (m), 874 (s), 832 (m), 807 (s), 767 (s), 742 (s), 722 (m), 710 (m), 637 (m).

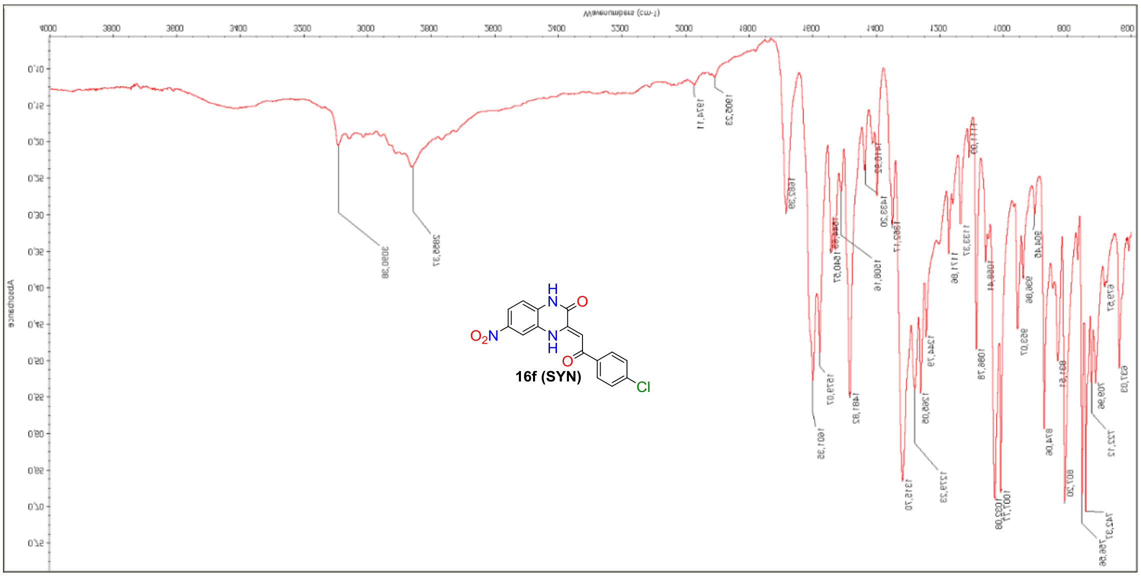

Figure S41. IR spectrum of compound **16f (SYN)**.

MS (ESI m/z): 342.0 [M-H]$^-$.

Anal. calcd for $C_{16}H_{10}ClN_3O_4$ (343.72): C, 55.98; H, 2.93; N, 12.23. Found: C, 56.14; H, 2.90; N, 12.05.

(*Z*)-3-(2-(4-Chlorophenyl)-2-oxoethylidene)-7-nitro-3,4-dihydroquinoxalin-2(1*H*)-one (17f (ANTI))

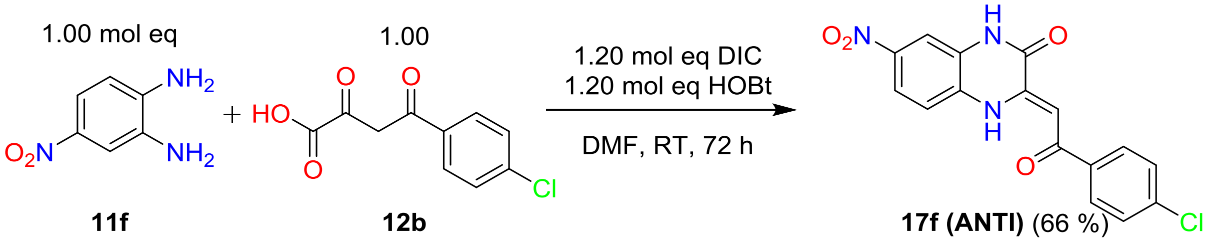

The 3,4-dihydroquinoxaline-2(1*H*)-one **17f (ANTI)** was prepared according to the general procedure B from acid **12b** diamine **11f**. The crude mixture of ANTI / SYN regioisomers was purified by trituration with boiling $CHCl_3$ yielding 100.0 mg (0.29 mmol, 66%) of **17f (ANTI)**.

Novelty: Compound **17f (ANTI)** was not described in the literature.

M.p.: 319.0 - 321.0 °C [$CHCl_3$], yellow solid compound.

NMR diagrams:

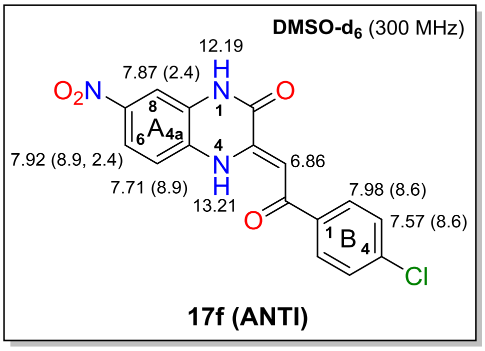

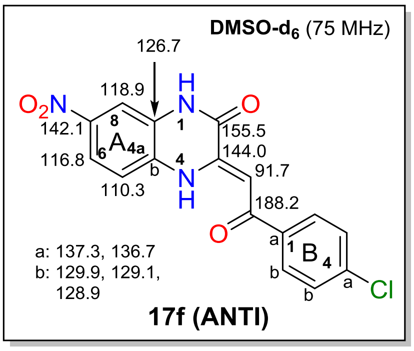

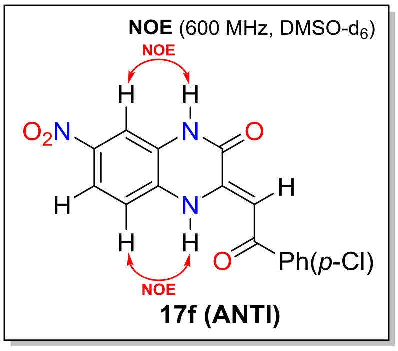

^{1}H NMR (300 MHz, DMSO-d_6): δ 13.21 (s, 1H, H-N_A(4)), 12.19 (s, 1H, H-N_A(1)), 7.98 (d, 2H, $J(B_2,B_3)$ = 8.6 Hz, 2 x H-C_B(2)), 7.92 (dd, 1H, $J(A_5,A_6)$ = 8.9 Hz, $J(A_6,A_8)$ = 2.4 Hz, H-C_A(6)), 7.87 (d, 1H, $J(A_6,A_8)$ = 2.4 Hz, H-C_A(8)), 7.71 (d, 1H, $J(A_5,A_6)$ = 8.9 Hz, H-C_A(5)), 7.57 (d, 2H, $J(B_2,B_3)$ = 8.6 Hz, 2 x H-C_B(3)), 6.86 (s, 1H, -COCH=).

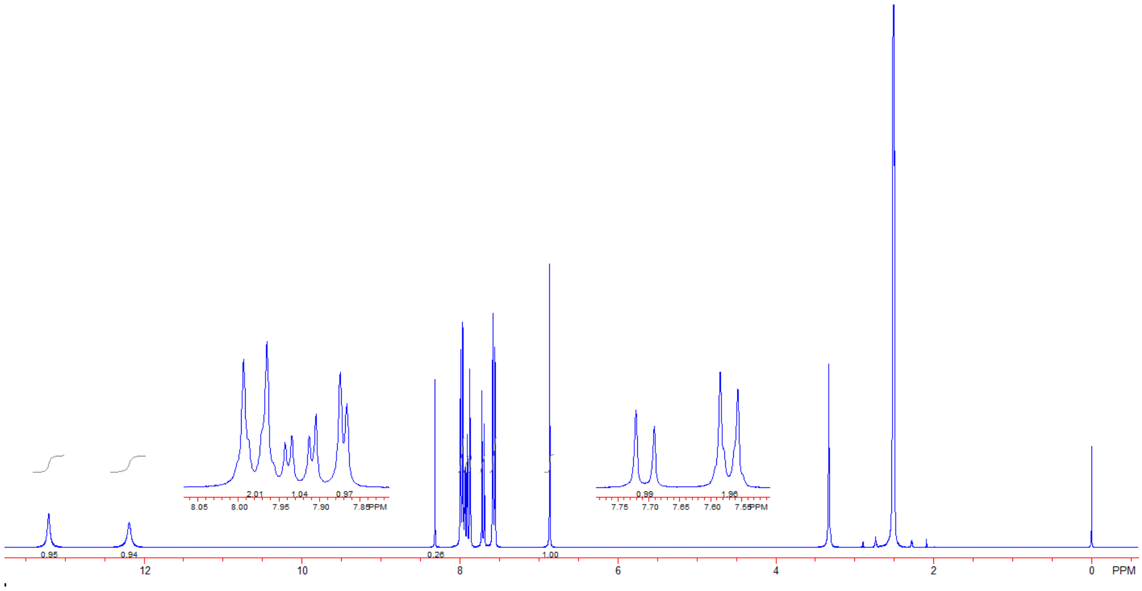

Figure S42. ^{1}H NMR (300 MHz, DMSO-d_6) spectrum of compound **17f (ANTI)**.

^{13}C NMR (75 MHz, DMSO-d_6): δ 188.2 (C_B(1)<u>C</u>=O), 155.5 (C_A(2)=O), 144.0 (C_A(3)), 142.1 (C_A(7)), 137.3 and 136.7 (C_B(1 and 4)), 129.9, 129.1 and 128.9 (C_A(4a) and 2 x C_B(2 and 3)), 126.7 (C_A(8a)), 118.9 (C_A(8)), 116.8 (C_A(6)), 110.3 (C_A(5)), 91.7 (-CO<u>C</u>H=).

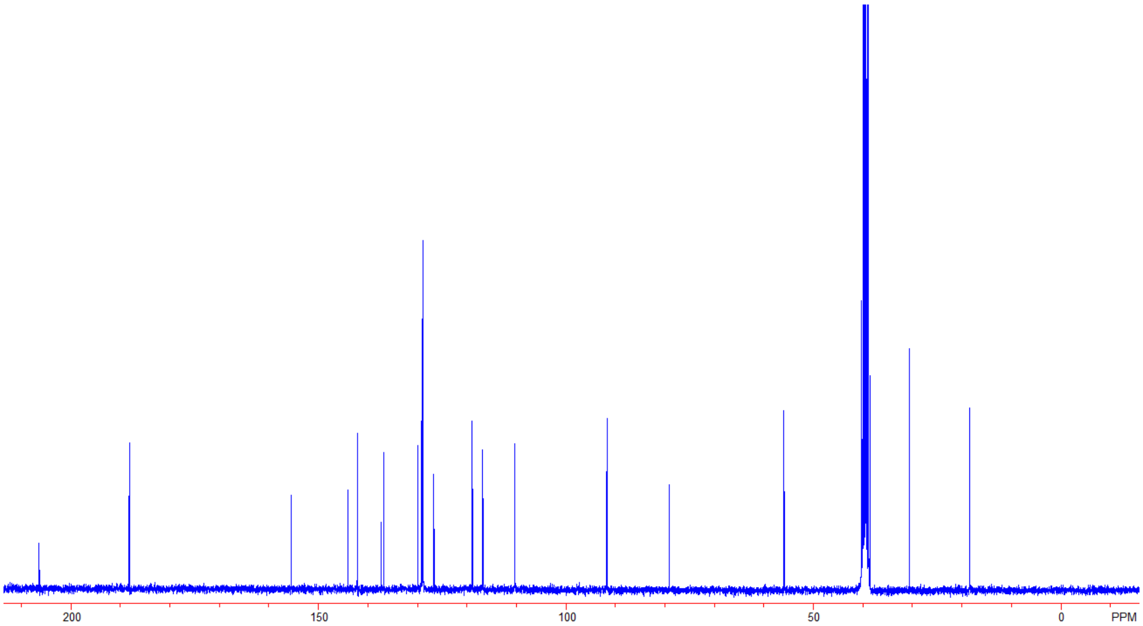

Figure S43. ^{13}C NMR (75 MHz, DMSO-d_6) spectrum of compound **17f (ANTI)**.

FTIR (solid, cm^{-1}): 3036 (m, NH), 2892 (m, NH), 2849 (m), 1693 (s, C=O), 1628 (m), 1605 (m), 1579 (s, NO_2), 1551 (m), 1528 (m), 1490 (w), 1458 (w), 1399 (m), 1332 (m), 1281 (m), 1255 (m), 1243 (m), 1182 (w), 1133 (w), 1089 (m), 1056 (m), 1010 (m), 963 (w), 886 (m), 846 (m), 809 (s), 740 (s) 669 (m).

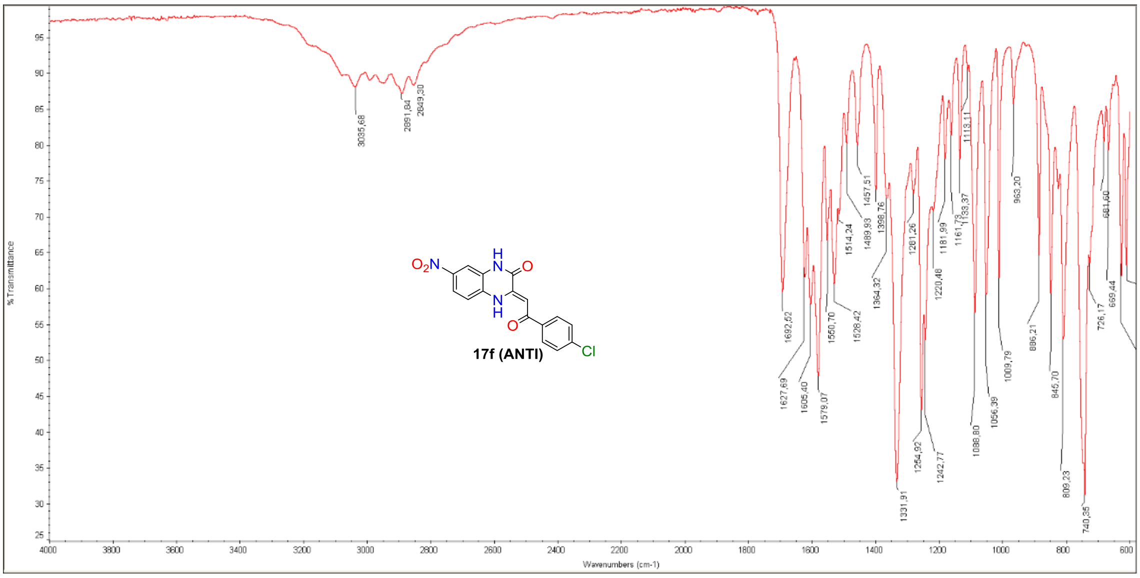

Figure S44. IR spectrum of compound **17f (ANTI)**.

MS (ESI m/z): 342.1 $[M-H]^-$.

Anal. calcd for $C_{16}H_{10}ClN_3O_4$ (343.72): C, 55.91; H, 2.93; N, 12.23. Found: C, 55.99; H, 3.05; N, 12.10.
